# Coding-Sequence Identification and Transcriptional Profiling of Nine *AMTs* and Four *NRTs* From Tobacco Revealed Their Differential Regulation by Developmental Stages, Nitrogen Nutrition, and Photoperiod

**DOI:** 10.3389/fpls.2018.00210

**Published:** 2018-03-05

**Authors:** Lai-Hua Liu, Teng-Fei Fan, Dong-Xue Shi, Chang-Jun Li, Ming-Jie He, Yi-Yin Chen, Lei Zhang, Chao Yang, Xiao-Yuan Cheng, Xu Chen, Di-Qin Li, Yi-Chen Sun

**Affiliations:** ^1^Department of Crop Breeding, College of Agriculture Sciences Hunan Agricultural University, Changsha, China; ^2^Department of Plant Nutrition, College of Resources and Environmental Sciences China Agricultural University, Beijing, China; ^3^Institute of Tobacco Research of Chongqing Tobacco Company China Tobacco Corporation, Chongqing, China

**Keywords:** tobacco, nitrate, ammonium, *AMT* and *NRT*, gene expression regulation, heterologous complementation, nitrogen and carbon balance

## Abstract

Although many members encoding different ammonium- and nitrate-transporters (AMTs, NRTs) were identified and functionally characterized from several plant species, little is known about molecular components for ${\text{NH}}_{4}^{+}$- and ${\text{NO}}_{3}^{-}$ acquisition/transport in tobacco, which is often used as a plant model for biological studies besides its agricultural and industrial interest. We reported here the first molecular identification in tobacco (*Nicotiana tabacum*) of nine *AMTs* and four *NRTs*, which are respectively divided into four (*AMT1/2/3/4*) and two (*NRT1/2*) clusters and whose functionalities were preliminarily evidenced by heterologous functional-complementation in yeast or Arabidopsis. Tissue-specific transcriptional profiling by qPCR revealed that *NtAMT1.1*/*NRT1.1* mRNA occurred widely in leaves, flower organs and roots; only *NtAMT1.1/1.3/2.1NRT1.2/2.2* were strongly transcribed in the aged leaves, implying their dominant roles in N-remobilization from source/senescent tissues. N-dependent expression analysis showed a marked upregulation of *NtAMT1.1* in the roots by N-starvation and resupply with N including ${\text{NH}}_{4}^{+}$, suggesting a predominant action of NtAMT1.1 in ${\text{NH}}_{4}^{+}$ uptake/transport whenever required. The obvious leaf-expression of other *NtAMTs* e.g., *AMT1.2* responsive to N indicates a major place, where they may play transport roles associated with plant N-status and (${\text{NH}}_{4}^{+}$-)N movement within aerial-parts. The preferentially root-specific transcription of *NtNRT1.1/1.2/2.1* responsive to N argues their importance for root ${\text{NO}}_{3}^{-}$ uptake and even sensing in root systems. Moreover, of all *NtAMT*s/*NRTs*, only *NtAMT1.1*/*NRT1.1/1.2* showed their root-expression alteration in a typical diurnal-oscillation pattern, reflecting likely their significant roles in root N-acquisition regulated by internal N-demand influenced by diurnal-dependent assimilation and translocation of carbohydrates from shoots. This suggestion could be supported at least in part by sucrose- and MSX-affected transcriptional-regulation of *NtNRT1.1/1.2*. Thus, present data provide valuable molecular bases for the existence of *AMTs/NRT*s in tobacco, promoting a deeper understanding of their biological functions.

## Introduction

Ammonium (${\text{NH}}_{4}^{+}$) and nitrate (${\text{NO}}_{3}^{-}$) are principal soil nitrogen (N) sources available to plants. To date, great research achievements have been made for our understanding of mechanisms related to N acquisition, translocation, utilization, and signaling throughout the plant (Schroeder et al., 2013; Vidal et al., 2014). As for the movement of N into and within the plant, the activity of different transport systems for ${\text{NO}}_{3}^{-}$ and ${\text{NH}}_{4}^{+}$ was extensively investigated (Nacry et al., 2013). Physiologically, certain transport systems were identified by the assay of ${\text{NO}}_{3}^{-}$/${\text{NH}}_{4}^{+}$ root-uptake kinetics, which can be categorized into two types: high-affinity transport systems (HATs) required for mediating most of the uptake activity at low external concentrations (up to 0.5 mM), and low-affinity transport pathways (LATs) responsible for a significant proportion of the N-uptake at concentrations normally above 0.5–1 mM (Nacry et al., 2013). Thus, most processes of ${\text{NO}}_{3}^{-}$/${\text{NH}}_{4}^{+}$ uptake/transport and assimilation were proved to be tightly controlled by the concentration of their substrates and/or whole-plant signal(s) of N status (Nacry et al., 2013). Besides, the acquisition of ${\text{NO}}_{3}^{-}$/${\text{NH}}_{4}^{+}$ by the plant can also be impacted by the photosynthesis, and displays a diurnal-rhythm pattern that is attributed to the regulation by shoot-to-root transport of carbohydrates (O'Brien et al., 2016). A recent study demonstrated that a transcription factor HY5 many serve as a shoot-to-root signal to induce AtNRT2.1 function in roots in response to light irradiation, enabling homeostatic maintenance of carbon (C)-N balance in varied light environments (Chen et al., 2016).

Molecularly, several families of genes encoding putative ${\text{NO}}_{3}^{-}$ and ${\text{NH}}_{4}^{+}$ permeases were cloned and characterized in many plant species. These permeases could be separated into two distinct groups i.e., ${\text{NH}}_{4}^{+}$ transporters (AMTs) and ${\text{NO}}_{3}^{-}$ transporters (NRTs). Furthermore, it is evident that spatiotemporal orchestration of multiple AMTs and NRTs may be key mechanisms underlying plant response, sensing, uptake and transport of N (Alvarez et al., 2012; Krapp, 2015). More recently, N sensors, transcription factors and further regulatory components were identified, showing a big puzzle that represents the efficient use of N by plants (Krapp, 2015).

The ${\text{NH}}_{4}^{+}$ transport gene family contains three main clades i.e., AMT, MEP (methylammonium permease-like subfamily), and Rh (rhesus-like proteins subfamily) (McDonald and Ward, 2016). In non-legume plants, the AMT family can be generally separated into two subgroups i.e., AMT1 and AMT2 (AMT2/3/4 cluster) (Koegel et al., 2013). Although AMT1 and AMT2 proteins might share a distant but common evolutionary origin, AMT1s seem to be more closely related to prokaryotic ${\text{NH}}_{4}^{+}$ transporters, whereas AMT2s more resemble some fungal proteins from leotiomyceta (von Wittgenstein et al., 2014). Both AMT1s and AMT2s show a high affinity (*K*_m_ in a micromolar range) and strong selectivity for ${\text{NH}}_{4}^{+}$, but those from the AMT2 group are unable to permeate ${\text{NH}}_{4}^{+}$-analog methylammonium. In higher plants, most members of the *AMT1* clade are preferentially expressed in roots, while a higher expression of most *AMT2* genes occur in shoots (Couturier et al., 2007). Arabidopsis *AMT1* or *AMT2* family comprises respectively five or single member(s), and these six *AMTs* with regulations at transcriptional, post-transcriptional and -translational levels were characterized in relatively more details (Neuhäuser et al., 2007; Lanquar et al., 2009). Importantly, AtAMT1.1 and AtAMT1.3 account for 30–35% of the capacity for ${\text{NH}}_{4}^{+}$ uptake in N-deficient roots and AtAMT1.2 for 18–26% (Yuan et al., 2007; Lanquar et al., 2009).

Regarding NRT families, 72 members were supposed to involve ${\text{NO}}_{3}^{-}$ uptake and translocation in Arabidopsis: NRT1/PTR (NPF, nitrate transporter 1/peptide transport family, 53 members), NRT2 (7 members), CLC (chloride channels, 7 members) and SLAC1/SLAH (slow anion channel-associated 1 homologs, 5 members) (Léran et al., 2014). Based on experimental evidence, many members of NRT1s and NRT2s were shown to mediate a proton-coupled active transport of ${\text{NO}}_{3}^{-}$ (Chen et al., 2008). Moreover, multiple ${\text{NO}}_{3}^{-}$-uptake transporters of the NRT1s and NRT2s could function together to enable an effective acquisition of N by plants, depending on organs/tissues, developmental stages and environmental conditions (Wang Y. Y. et al., 2012) at a transcriptional level, some NRTs (e.g., AtNRT1.1, 1.4, 1.6, 2.1) were either co-ordinately or differentially regulated by nitrate, N metabolites, N-starvation, circadian clock, sucrose and pH (Lejay et al., 1999; Krouk et al., 2010; Medici and Krouk, 2014). Molecularly, most of the NRT1s characterized so far are low affinity ${\text{NO}}_{3}^{-}$ permeases except for AtNRT1.1/MtNRT1.3, which are dual-affinity ${\text{NO}}_{3}^{-}$ transporters (Liu et al., 1999; Liu and Tsay, 2003; Morère-Le Paven et al., 2011). Interestingly, AtNRT1.1 was demonstrated to be a ${\text{NO}}_{3}^{-}$ sensor to monitor changes in external ${\text{NO}}_{3}^{-}$ concentrations to promote proper metabolic acclimation, thus termed “transceptor” (Ho et al., 2009). So far, plant NPF families were shown to incorporate transporters not only for ${\text{NO}}_{3}^{-}$ but other substrates e.g., peptides, amino acids and hormones (O'Brien et al., 2016). Unlike NRT1s, most known NRT2s displayed a much stronger specificity for ${\text{NO}}_{3}^{-}$ with a high-affinity; however, most NRT2 proteins alone did not show any ${\text{NO}}_{3}^{-}$ transport activity when lacking interaction with a membrane protein NAR2 (Wang Y. Y. et al., 2012).

Tobacco is used as one of good model species in the plant biological study and also an industrially interested crop (Sierro et al., 2014). Its agricultural production consumes large amounts of N-fertilizers (with c. 150,000 tons net N annually, http://www.fao.org/faostat/), but over a half of applied N lost into environments due to at least in part inefficient N capture and utilization by plants (Sisson et al., 1991). Additionally, N nutrition strongly impacts the content and composition of N-containing compounds e.g., nicotine and aromatic heterocyclic substances in tobacco products, whose biological activities related to their molecular formation and degradation are fairly interesting for (bio-)chemists (Schmeltz and Hoffmann, 1977). However, besides limited early studies showing sequence identification, regulation and functionality of NRT2.1 from Nicotiana plumbaginifolia (Quesada et al., 1997; Krapp et al., 1998; Fraisier et al., 2000), possible mechanisms responsible for N-acquisition by roots and its translocation between tissues/organs of tobacco are little known at molecular and physiological levels. In this work, we performed a homologous sequence search to identify putative coding-sequences of *AMT* and *NRT* genes from database of *Nicotiana tobacum* L. cv. K326 (a worldwide cultivated tobacco variety); and their transport activities were preliminarily conformed by gene-cloning and heterologous functional-complementation of yeast and Arabidopsis. Furthermore, we conducted phylogenetic and gene-structure analyses across representative monocots and dicots to assign a nomenclature to individual tobacco AMTs and NRTs identified, and whose transport activity was preliminarily evaluated using yeast and Arabidopsis expression systems. Moreover, the quantitative RT-PCR was applied to examine expression patterns of all *AMT*s and *NRT*s in the variety K326 in a manner of tissue-specificity, nitrogen- and diurnal-dependency. Thus, the present study provides not only experimental evidence for the existence of different tobacco *AMTs* and *NRT*s, but also valuable genetic information for further comprehensively understanding of processes related to N transport and utilization in tobacco.

## Materials and methods

### Homologs sequence search

Sequences of tobacco putative *AMT* and *NRT* genes and their corresponding expressed sequence tag(s) (EST) were obtained from NCBI GenBank and *Nicotiana tabacum* L. cv. K326 genome database “SOL Genomics Network” (http://solgenomics.net/) via BLAST search, for which amino acid-sequence of some functionally characterized *AMT* and *NRT* proteins of Arabidopsis, rice and tomato were used as templates (e.g., *AtAMT1.1/2.1, OsAMT3.1, LeAMT1.1, PtrAMT4.1, AtNRT1.1/2.1*). The genomic localization and exon-intron structure of tobacco *AMTs* and *NRTs* were identified by using a gene-prediction web server (http://genes.mit.edu/GENSCAN.html; Burge and Karlin, 1997) as well as an alignment of *AMTs* and *NRTs* to the genomic sequence of K326.

### Phylogenetic analysis and protein topology prediction

Homologous sequences of NRTs and AMTs from different plant species were obtained from database (Aramemnon, NCBI) and certain publications (Orsel et al., 2002; Koegel et al., 2013). ClustalW method in DNASTAR Lasergene 8 was used to perform a multiple protein-sequence alignment, with parameters: gap opening penalty 15, gap extension penalty 0.3, 25% of delay divergent sequences, and Gonnet series as the protein weight matrix. The phylogenetic tree of AMTs or NRTs was constructed by using neighbor-joining algorithm in MEGA 6.0 software. Bootstrap analysis was conducted with 1,000 replicates, and branch lengths are proportional to phylogenetic distances. Peptide hydropathy or protein topology was examined using TMHMM v2. (http://www.cbs.dtu.dk/services/TMHMM/) in “CBS prediction servers.”

### Plant growth condition

For obtaining different plant aerial tissues/organs, seeds of tobacco (K326) were sowed and cultivated for 3 months in pots (0.30 m diameter, 0.35 m height) filled with 9 kg soil (80% peat, 20% vermiculite, soil moisture 60–70%) in an outside glasshouse (without additional lighting, 25–28/18–21°C in a natural darklight period, 55–58% relative humidity). Flower tissues including petal, calyx, pedicel and ovary, young leaves (the 3th leaf counted down from the top first fully-expended leaf), mature leaves (the 10th leaf) and old leaf (the 18th leaf) were collected, frozen quickly in liquid N and stored at −80°C for later total RNA isolation and expression study.

For N- and diurnal-dependent gene expression studies, seeds were surface sterilized (with 70% ethanol for 2 min and further with 2% sodium hypochlorite solution for 20 min, rinsing five times with sterile water) and germinated on a moistened paper for 10 d in a growth chamber [15 h light/ 9 h dark circle, about 250 μE m^−2^ s^−1^ light intensity, 25–27/22–23°C in a light/dark period (light period 08:00-23:00), and 60% relative humidity]. Seedlings were transferred first to a 1/4 strength of normal nutrition solution (containing K_2_SO_4_ 0.8 mM, KH_2_PO_4_ 1 mM, NH_4_NO_3_ 1 mM, MgSO_4_ 1.5 mM, CaCl_2_ 2 mM, MnSO_4_ 3 μM, ZnSO_4_ 1 μM, CuSO_4_ 0.1 μM, (NH_4_)_6_Mo_7_O_24_ 0.1 μM, Fe-EDTA 30 μM, H_3_BO_3_ 1 μM, pH 6.1-6.3 adjusted using Tris-buffer) for 1 week growth; thereafter, plants of a similar size were chosen and grew on an aerated normal nutrition solution, which was refreshed every 2 d. In the N-treatment experiment, control plants were maintained in normal growth medium containing 1 mM NH_4_NO_3_ for 18 or 20 d, while treatment plants were transferred to N-free solution for 3 or 1 d growth; after 3 d N-starvation, the plants were resupplied with 2 mM N in the form of KNO_3_ or (NH_4_)_2_SO_4_ for 1 h or 4 h or 12 h, or urea or glutamine (Gln) for 4 h. Roots and leaves (the 2nd and 3rd fully-opened leaves counted down from the top) were sampled separately, frozen with liquid N immediately and stored at −80°C for later use. In the diurnal-dependent experiment, plants were cultured in the normal nutrient solution for 3 weeks; the roots and leaves were separately harvested at 2:00, 6:00, 10:00, 14:00, and 20:00, frozen quickly in liquid N, and stored at −80°C for gene expression analysis.

In the MSX- and Suc-experiment, plants were hydroponically grown (for 15 d) in normal nutrient solution as described above and then treated with 1% sucrose or 1 mM MSX for a time period of 0 h (start from 10:00), 2 h, 4 h, or 6 h. At each time point excepted for time 0 h, no-treatment plants were also harvested as control samples. Roots and leaves (the 2nd and 3rd fully-opened leaves counted down from the top) were harvested at 10:00, 12:00, 14:00, and 16:00, frozen quickly in liquid N and stored at −80°C for later use.

### Quantitative RT-PCR assay

Total RNA was extracted using Easyspin RNA Kit (AIDLAB). RNase-free DNase I was used for removal of DNA contamination in RNA samples. First strand cDNA synthesis was done with 2 μg total RNA as a template using M-MLV Reverse Transcriptase (Promega) according to the manufacturer's protocol. qPCR was conducted in 20 μL volume (containing 2 μL of 1:10 diluted original cDNAs, 200 nM of each gene-specific primer, and iQ^TM^ SYBR Green Supermix from Bio-Rad) using a Bio-Rad iCycler. PCR cycling parameters were set as following: 95°C for 5 min, 40 cycles of 30 s at 94°C, 30 s at 57°C, and then a final melting curve of 65–95°C. Gene specific primers used in the qPCR experiment are listed in Table S3. The relative expression level of *NtAMTs* and *NtNRTs* was normalized to that of a stable internal reference gene α*-tubulin* (Schmidt and Delaney, 2010). The efficiency of the primers ranges from 95 to 105% when tested and calculated using the relevant standard curve method described by Larionov et al. (2005).

To assure the reliability of qPCR results by using *tubulin* gene as a reference, a second house-keeping gene i.e., ribosomal protein gene *L25* (Schmidt and Delaney, 2010) was applied (representative results for certain genes or treatments were shown in Figure S2). The qPCR reaction was done in 3-4 biological replicates for each plant treatment, together with three “no template control” to examine a cross contamination of reagents. Data were analyzed by using the 2^−Δ*CT*^ method (CT, cycle threshold) (Schmittgen and Livak, 2008).

### Cloning, yeast, and arabidopsis functional complementation

The putative ORF of *NtAMT1.1/1.21.3/2.1/3.1/4.1/4.3* and *NtNRT1.1/1.2* was amplified by RT-PCR performed with a total RNA prepared from tobacco (K326) as stated in the “Quantitative RT-PCR assay” using the specific primers containing the *Bam*HI or *Pst*1 (only for *NtAMT4.1*) site (Table S3). The amplified DNA fragment of individual genes was constructed into pEASY-T3 (TRANSGENE Biotech, China) and sequenced for its correctness. The ORF of *NtAMT1.1, 1.2, 1.3, 2.1, 3.1, 4.1*, and *4.3* with a *Bam*HI or *PstI* overhang was ligated into the yeast expression vector pHXT426 (Liu et al., 2003) after its linearization by *Bam*HI or *PstI*. The resulting plasmid was transformed into the triple *mep* deletion yeast strain 31019b (*mep1-3*, Δ*ura3*), which can not grow on <5 mM ${\text{NH}}_{4}^{+}$ as only N source (Marini et al., 1997), and the pHXT426 was respectively introduced into the yeast strain 23346c (Δ*ura3*) (Liu et al., 2003) and 31019b as a positive and negative control. All transformants were first selected on SD agar (Oxid)-medium (2% agar, 2% glucose, 0.17% yeast nitrogen base without amino acids and ammonium sulfate; Difco, Detroit, USA) consisting of 10 mM (NH_4_)_2_SO_4_ as N source. A single colony was picked, suspended in 15 μl water, streaked onto the solid SD agar-medium supplied with 2 mM NH_4_Cl as the sole N source.

The ORF of *NtNRT1.1/1.2* were amplified by PrimerSTAR HS DNA Polymerase (Takara) using gene specific primers containing *BamHI* site (Table S3) and cloned into a plant expression vector pCF203^−^ (in which *GFP* gene was removed, Wang W. H. et al., 2012) using *Bam*HI site, to obtain constructs termed pCF203^−^35S_pro_:*NtNRT1.1*:rbcs_term_ and pCF203^−^35S_pro_:*NtNRT1.2*: rbcs_term_. A mutant line of Arabidopsis *AtNRT1.1* (*atnrt1.1-1*, Hachiya et al., 2011) was transformed by dipping inflorescences into a cell suspension (OD_600_ at c. 0.6) of *Agrobacterium* GV3101 harboring the above constructs. Since the T-DNA in the vector and *atnrt1.1*-1 carry the same Kanamycine-resistant marker gene, several transformants were selected out by PCR test using a primer (5′-TCCGTATGTTGCATCACCTTCAC-3′) annealing to the vector T-DNA and another specific for *NtNRT1.1* (5′-CGggatccATGGCACTTCCTGAAACACA-3′) or *NtNRT1.2* (5′-TTggatccATGGCACTTCCTGAGACA-3′). Independent homozygous *NtNRT1.1-/1.2*-transformed lines were generated in T2 generation for experiments.

For the growth complementation test, surface-sterilized *Arabidopsis* seeds [wild type (Col-0), *atnrt1.1-1, atnrt1.1-1*+*NtNRT1.1* (2, 3) and *atnrt1.1*+*NtNRT1.2* (2, 5)] were germinated for 5 d on 1/2 MS (N-free) agar-medium containing 1 mM NH_4_NO_3_ as N source, thereafter seedlings were transferred to vertical plates containing 1/2 MS (N-free) supplied respectively with 0.1 or 5 mM KNO_3_ for a 10 d growth. Shoots and roots were harvested separately for biomass determination.

### Statistical analysis

Statistical test was performed using the statistical software program SPSS version 16.0 (Beijing, China). Significant differences between treatments were determined by one-way analysis of variance (ANOVA), and *post-hoc* comparisons were carried out using Tukey's multiple range test at *P* < 0.05.

## Results

### Identification of putative coding sequences for ammonium- and nitrate-transporters from tobacco

**Figure 1:**
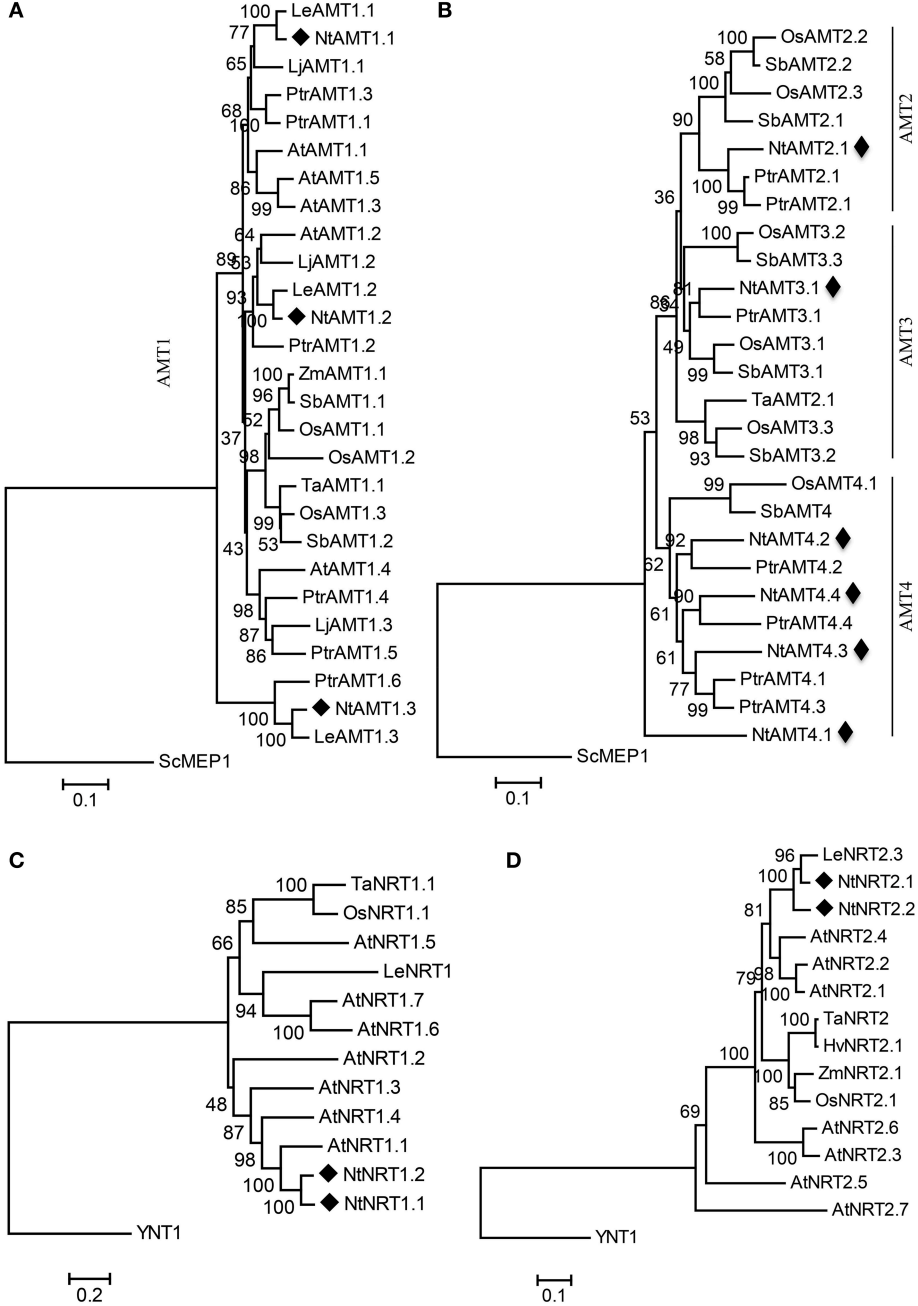
Phylogenetic tree of putative tobacco AMTs **(A,B)** and NRTs **(C,D)** with their representative counterparts from other plant species. The trees of AMT1 **(A)** and AMT2, 3, 4 **(B)** subclades were rooted using a *Saccharomyces cerevisiae* ScMEP1 sequence as an outgroup. Likewise, *Hansenula polymorpha* YNT1 protein was picked as an outgroup for NRT1 **(C)** and NRT2 **(D)** subcluster. Phylogenetic analysis was performed using the Neighbor-Joining method from MEGA6. Bootstrap values are from 1,000 replications. Evolutionary distances were estimated in a unit of the number of amino acid substitutions per site, with a scale bar equivalent to 0.1 or 0.2 substitutions per site. The numbers at the nodes are bootstrap values. Sequences and accession numbers of AMTs and NRTs were listed in Tables S2, S4. Sc, *Saccharomyces cerevisiae*; At, *Arabidopsis thaliana*; Lj, *Lotus japonicas*; Le, *Lycopersicon esculentum*; Os, *Oryza sativa*; Ptr, *Populus trichocarpa*; Sb, *Sorghum bicolor*; Ta, *Triticum aestivum;* Hv, *Hordeum vulgare*; YNT1 from *Hansenula polymorpha*. Nt, *Nicotiana tabacum* L. (cv K326). NtAMTs and NtNRTs are marked with a black rhombus.

To explore molecular bases of ${\text{NH}}_{4}^{+}$ and ${\text{NO}}_{3}^{-}$ movement in tobacco, sequences of Arabidopsis, rice and poplar ammonium- and nitrate-transporters (e.g., AtAMT1.1, AtAMT2.1, OsAMT3.1, PtrAMT4.1, AtNRT1.1, and AtNRT2.1) (http://aramemnon.uni-koeln.de/index.ep) were used as references to search for homologous sequences in tobacco database at Sol Genomics Network (http://solgenomics.net) using an *E*-value cutoff of 1e-5. Consequently, by means of a web server-based gene prediction (http://genes.mit.edu/GENSCAN.html), 9 and 4 coding-sequences (or open reading frames, ORFs) homologous respectively to AMT and NRT were extracted from tobacco (*N. tabacum* L. cv. K326). Based on sequence homology analysis at an amino-acid level, we termed here such tobacco orthologs as NtAMT1-4 and NtNRT1-2 (Table 1; Figure 1). Transcriptionally, some corresponding expressed sequence tags or cDNA clones for every *NtAMTs* and *NtNRTs* identified here could be found in EST databases (Table 1).

**Table 1:** Characteristics of *AMT* and *NRT* homologous genes in tobacco.

**Gene name**	**ORF size (bp)**	**Number of deduced amino acid residues**	**EST with accession number**	**Exon position in a genomic DNA contig(s) from K326**
NtAMT1.1	1,473	490	DW001426.1 (3′) (K326)	AWOJ-SS14797 (195908–197330)
NtAMT1.2	1,539	512	EB445104.1 (3′) (K326)	AWOJ-SS18441 (489118–488014, 490810–490371)
NtAMT1.3	1,395	464	CK293460.1 (5′), CK288135.1 (3′), DW000624.1 (K326)	AWOJ-SS1877 (53449–54843)
NtAMT2.1	1,470	489	FG135933.1 (5′), EB443094.1 (3′) (K326)	AWOJ-SS269362 (31827–32470, 35423–35862, 33446–33733, 34250–34357)
NtAMT3.1	1,461	486	FG144466.1 (5′), CK294598.1 (3′),	AWOJ-SS1274 (1158464–1158092, 1155881–1155630, 1156946–1156658, 1155059–1154787, 1159508–1159384)
NtAMT4.1	1,413	470	CK294598.1 (3′), CK284369.1 (3′)	AWOJ-SS4788 (25826–25290, 27887–27599, 30084–29489)
NtAMT4.2	1,488	495	BP135736.1 (3′)	AWOJ-SS5547 (33695–34332, 36252–36818, 35022–35310)
NtAMT4.3	1,422	473	AM826377.1 (5′)	AWOJ-SS14683 (110502–111046, 108872–109353, 109868–110155, 108457–108576)
NtAMT4.4	1,431	476	EG013243.1 (5′), AM841893.1	AWOJ-SS14196 (31349–31939, 32817–33104, 33270–33829)
NtNRT1.1	1,773	590	EB447264.1 (3′) (K326), BP746990.1 (3′)	AWOJ-SS894 (79324–79442, 79649–79829, 80652–81221, 83119–83987)
NtNRT1.2	1,785	594	BP750329.1, EB447264.1 (3′) (K326)	AWOJ-SS15736 (119215–118338, 121370–120798, 122479–122297, 123306–123185, 122157–122115)
NtNRT2.1	1,593	530	BP748887.1 (3′) EB683986.1 (3′) (K326)	AWOJ-SS1357 (486674–487483, 487828–488498, 487593–487713)
NtNRT2.2	1,593	530	BP752802.1 EB683986.1 (3′) (K326)	AWOJ-SS4175 (225048–225857, 226947–227616, 2261270–226250)

*Data were extracted mainly from genome databases of Nicotiana tabacum L. cv. K326 and Nicotiana sylvestris (e.g., some ESTs). The name of tobacco AMT and NRT genes is given based on analysis of the sequence similarity to their counterparts from certain plant species published (see Figure 1). ORF, open reading frame; EST, expressed sequence tag*.

Molecularly, surveying of genomic organization revealed that the putative ORF of *NtAMTs* and *NtNRTs* might be spliced from 1 to 5 exons, except for *NtAMT1.1* and *NtAMT1.3* (no intron in their genomic organization) (Table 1). The length of *NtAMTs'* or *NtNRTs'* ORFs was respectively predicted to range from 1,395 to 1,539 or 1,593 to 1,785 bp, which encode peptides with 464–512 or 530–594 deduced amino acids (Table 1). Furthermore, protein hydrophobicity analysis using “CBS prediction servers” (see section Materials and Methods) showed that NtAMT1.1, NtAMT1.3, and NtAMT4.2 were predicted to span a biological-membrane 10 times, and 11 transmembrane domains (TMD) for NtAMT1.2, 2.1, 3.1, 4.1, 4.3, and 4.4 (Figure S1). Regarding tobacco NRTs, NtNRT1 and 2 possess 12 predicted TMDs, especially for NtNRT1.1 and 1.2 with a relatively large hydrophilic-loop located between TMD6 and TMD7 (Figure S1), similar to their orthologs from Arabidopsis (Tsay et al., 2007). These topological inspection data indicate a typical nature of integral membrane proteins for tobacco AMTs and NRTs identified.

### Tobacco AMT or NRT homologs are phylogenetically separated into different clusters

To appreciate an evolutionary relationship and diversification of genes encoding tobacco putative AMTs and NRTs, amino acid sequences of 57 AMT proteins and 17 NRT peptides published for varied organisms ranging from plants (e.g., Arabidopsis, rice, wheat, barley, tomato, etc.), yeast, and bacterial (Orsel et al., 2002; Koegel et al., 2013; Buchner and Hawkesford, 2014; von Wittgenstein et al., 2014) were collected to perform a homologous alignment (using ClustalW from Lasergene 8), which was then used to generate a rooted-phylogenetic tree by the Neighbor-Joining method implemented in MEGA6 (Figure 1) (Koegel et al., 2013).

Resulting phylogenetic data showed that 55 AMT proteins (Table S4) from 8 plant species including those identified from tobacco could be divided into two major groups (termed here as AMT1 and AMT2-4) (Figures 1A,B), which were evolutionally diverged relatively far from yeast ScMEP1-2 (methylamine permease) with a highest sequence homology <25% (Table S1A). Phylogenetically, AMT1 seems to be separated quite far from AMT2-4 due to sharing only a low sequence identity of c. 20% [Table S1A, thus the phylogram of AMT1 was separately constructed (Figure 1A)], whereas the lineage between AMT2-4 is closely related by showing their homology by 50-80% (Table S1A). As presented in Figures 1A,B, nine NtAMTs are more closely related to the respective isoforms from tomato (Le, *Lycopersicon esculentum*) and poplar (Ptr, *Populus trichocarpa*) than to each other. They are distributed in all four AMT-subfamilies (i.e., NtAMT1.1-1.3, NtAMT2.1, NtAMT3.1, NtAMT4.1-4.4), indicating an evolutional divergence of ammonium transporters prior to the split between monocot and eudicot.

Similarly, based on homology analysis the collected plants' NRTs comprising 4 putative tobacco homologs could be split into two subgroups (NRT1, NRT2; Figures 1C,D), which strayed genetically away from that in unicellular organisms (e.g., YNT1 from yeast *Hansenula polymorpha* and NARK from bacterial *Escherichia coli*), with sequence homologies of only 6.3–12.8% to NRT1 and 11.2–24.2% to NRT2 (Table S1B). Plant NRT2 subcluster appears to be closer to yeast YNT1 (sharing 21.5–24.2% sequence identity) than NRT1 to YNT1 (with 7.3–12.8% homology) (Table S1B). Because of a strong separation between plant NRT1 and NRT2 by YNT1 (or no point to join NRT1 and NRT2 groups in a rooted phylogram due to their low sequence identity at 3.4–10.8%. Table S1B), we constructed respectively the phylogenetic tree for NRT1 and NRT2 (Figures 1C,D). NtNRT1.1 and 1.2 or NtNRT2.1 and 2.2 exhibit respectively the highest sequence similarity to Arabidopsis AtNRT1.1 (66.3% identity) or to tomato LeNRT2.3 (82% homology) (Table S1B).

### *NtAMTs* and *NtNRTs* are differentially transcribed in different aerial organs/tissues of tobacco

**Figure 2:**
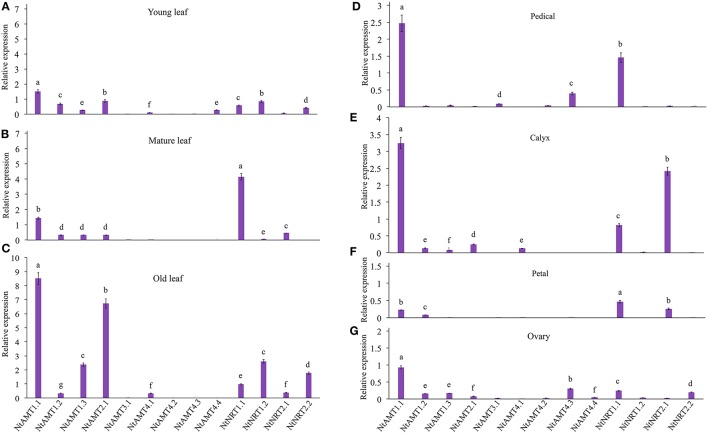
Measurement of the gene expression of *AMTs* and *NRTs* in different aged leaves and flower-tissues. Tobacco (K326) was grown in pot-soil for 3 months until flowering (see section Materials and Methods). Relative mRNA accumulation of *AMTs* and *NRTs* was assayed by using qPCR (see section Materials and Methods), which was performed with total RNA isolated from petals, calyxes, pedicels, ovaries, young leaves (the 3th leaf counted down from the top first-full-expended leaf), mature leaves (the 10th leaf) and old leaf (the 18th leaf), respectively. Gene-specific primers for a given *AMT* or *NRT* were used throughout this study (see Table S3). Before performing qPCR, the correctness of resulting amplicons of target genes was confirmed by DNA sequencing (note: this test was done throughout expression studies). Relative expression levels of *AMTs* and *NRTs* relative to that of tobacco α*-tubulin* (set to 1; Schmidt and Delaney, 2010) were calculated. Means of 3–4 biological replicates ± *SD* (*n* = 3–4) were plotted, and different letters above the bars indicate statistically significant differences (*P* < 0.05 by one-way ANOVA and a multiple comparison test). A second housekeeping gene *L25* was also used as an internal control to confirm the expression pattern (Schmidt and Delaney, 2010) (Figures S2A,B). The relative expression of NtAMTs and NtNRTs in mature leaf **(A)**, young leaf **(B)**, old leaf **(C)**, petals **(D)**, calyxes **(E)**, pedicels **(F)** and ovaries **(G)**.

To help understand putative physiological roles of NtAMTs and NtNRTs in ${\text{NH}}_{4}^{+}$ and ${\text{NO}}_{3}^{-}$ transport processes in tobacco (K326), gene expression studies in a spatiotemporal manner were conducted with total RNA from varied aerial part tissues, including differently aged leaves and flower organs of plants grown on pot-soil. Quantitative real-time RT-PCR (qPCR) analyses revealed that mRNA of most identified *NtAMTs* and *NtNRTs* in young or mature leaves occurred at low levels (with a relative expression level < 1), except for *NtAMT1.1* and *NtNRT1.1* (with higher mRNA accumulation in mature leaves, especially for *NtNRT1.1*) (Figures 2A,B), but in old leaves a strong expression of *NtAMT1.1*/*2.1* (with a relative expression level >6.5) and relatively more transcripts of *NtAMT1.3, NtNRT1.2/2.2* (with a relative expression level >2) were measured (Figure 2C). In flower tissues, *NtAMT1.1* and *NtNRT1.1* or *NtAMT1.1* and *NtNRT2.1* with obviously higher expression levels (>1.5-fold) were assayed in only pedicels or calyxes (Figures 2D–E), respectively; in both petals and ovaries all identified *NtAMTs* and *NtNRTs* were less expressed, except for *NtAMT1.1* with a relatively high expression level at 1-fold in ovaries (Figure 2G). These tissue-/organ-specific expression measurements might provide a valuable overview about possible physiological roles of such AMTs and NRTs in ${\text{NH}}_{4}^{+}$ and ${\text{NO}}_{3}^{-}$ movement into and/or within tobacco plant cells, depending on their subcellular localizations, which will have been described in our coming works. For rapidly getting a global view of tissue-specific expression patterns of all *NtMATs* and *NtNRTs*, a heat map summarizing transcriptional variations of these 13 genes can be further referred to supporting information (Figure S3A).

### The expression of *NtAMTs* and *NtNRTs* is differently regulated by varied nitrogen-nutritional status

**Figure 3:**
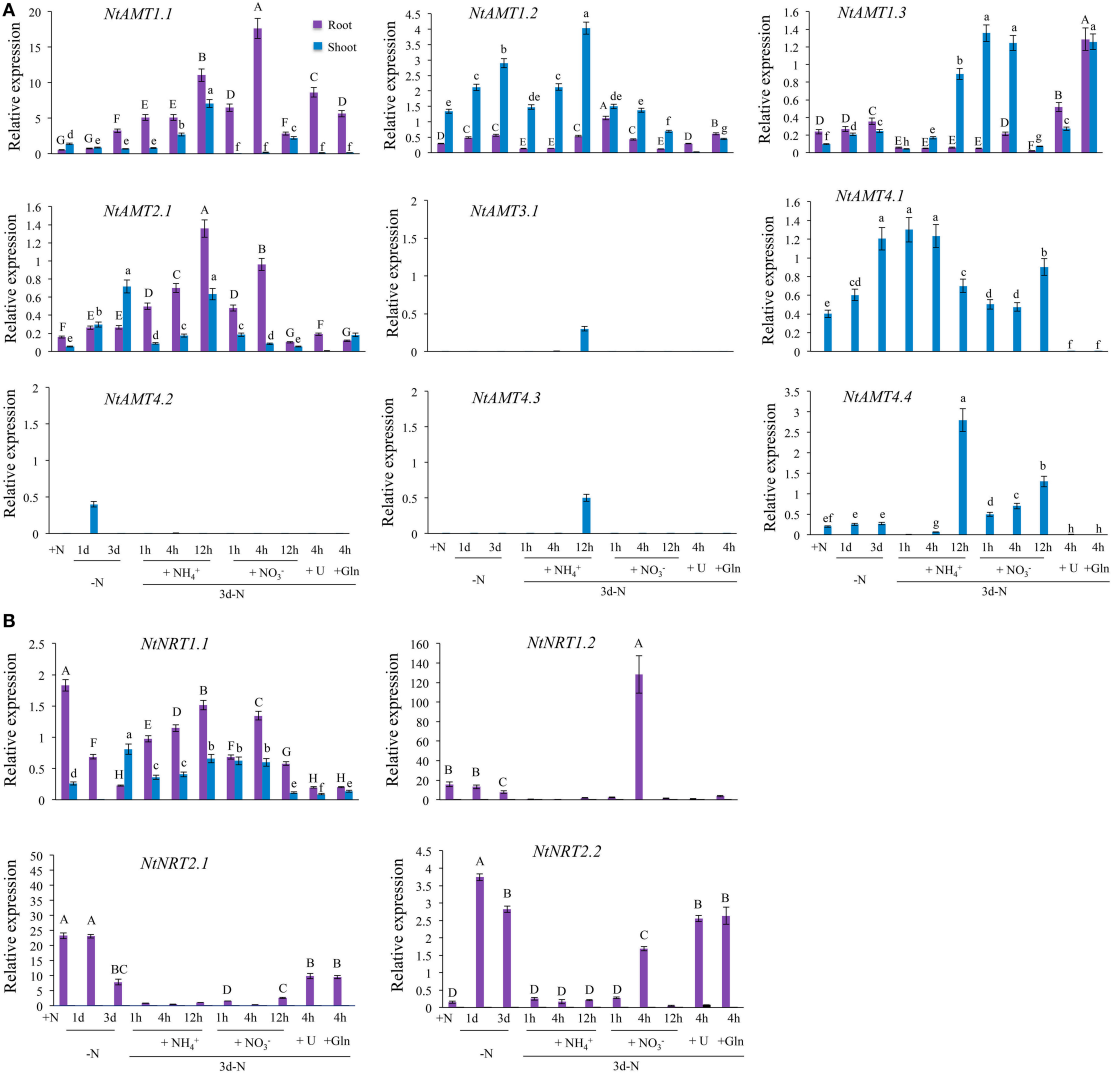
N-dependent expression of *AMTs* **(A)** and *NRTs* **(B)** in roots and leaves of tobacco K326. Plant growth and N-treatment are described in section “Materials and Methods”. Total RNA was extracted from roots or leaves (1 d after fully-opened), and relative mRNA abundance of *NtAMTs* and *NtNRTs* was quantified by qPCR (see section Materials and Methods). Expression levels of *AMTs* and *NRTs* relative to that of α*-tubulin* (set to 1) were calculated. Data are means of 3–4 biological repeats ± *SD* (*n* = 3–4); different letters above the bars indicate statistically significant differences (*P* < 0.05 by one-way ANOVA and a multiple comparison test). +N, growth with normal N treatment; -N, N-starvation; 1d-N and 3d-N, N-starvation for 1 d or 3 d; N-resupply with ${\text{NH}}_{4}^{+}$ (for 1, 4, 12 h), ${\text{NO}}_{3}^{-}$ (1, 4, 12 h), urea (4 h), and Gln (4 h) after 3d-N. *L25* was also applied as a reference to confirm the expression pattern (Figures S2C,D). Purple or blue bars indicate respectively root and leaf.

To comprehend a molecular response of tobacco *AMTs* and *NRTs* to plant N-nutritional status, we analyzed the gene expression using qPCR performed with total RNA from tobacco K326 subjected to varied N-regime treatments after a hydroponic pre-culture of plants for 18–21 days (see section Material and Methods). In roots, transcripts of *NtAMT1.1, 1.2, 1.3, and 2.1* were detectable but *NtAMT3.1, 4.1, 4.2, 4.3*, and *4.4* not (Figure 3A); upon N-depletion (for 1 or 3 d), an obvious transcriptional up-regulation was assayed only for *NtAMT1.1* (with its relative expression level >2) (Figure 3A); resupply of ${\text{NH}}_{4}^{+}$ (e.g., 12 h) or ${\text{NO}}_{3}^{-}$ (4 h) to the plants (starved of N for 3 d) stimulated 4- to 6-folds higher expression of *NtAMT1.1* and *2.1* as compared to that of in the control, while 12 h ${\text{NO}}_{3}^{-}$-resupply decreased *NtAMT1.1* and *2.1* expression (Figure 3A); interestingly, the expression of *NtAMT1.1* and *1.3* could be up-regulated by the addition of 1 mM of other N forms e.g., urea or glutamine (Gln) (Figure 3A).

In leaves, the expression of only *NtAMT1.2/2.1/4.1/4.2* was markedly elevated under N starvation of plants for 1 d (except for *NtAMT2.1*) or 3 d (except for *NtAMT4.2*) (Figure 3A). In the plants re-provided with ${\text{NH}}_{4}^{+}$ for 12 h, mRNA level of *NtAMT1.1/1.2/1.3/3.1/4.3/4.4* was increased respectively by about 6.8-, 1.5-, 2.8-, 29-, 47-, 42-fold as compared to that of in the control plants (N-starved of 3-d) (Figure 3A), but the relative expression of *NtAMT2.1/4.1/4/2* was very much low (Figure 3A). As resupplied with ${\text{NO}}_{3}^{-}$ to 3-d N-starved plants for 12 h, *NtAMT1.1/4.4* expression were significantly up-regulated (3-5 folds higher than the control), whereas *NtAMT1.2/1.3/2.1/4.1* mRNA levels were obviously decreased (Figure 3A), and *NtAMT2.1/3.1/4.2/4.3* transcripts were hardly detected (Figure 3A); when the plants were subjected to 3 d N-starvation, the presence of urea or Gln in the medium (for 4 h) induced a higher expression (3-fold more than the control) of *NtAMT1.3*, but greatly suppressed the transcription of *NtAMT1.2/2.1/4.1*. To help comprehend briefly the complex N-dependent transcriptional-regulation of identified *NtAMTs* and also *NtNRTs* (see later), an outline heat map is provided in supporting information (Figure S3B).

Regarding *NtNRTs* in response to N-treatments, only *NtNRT1.1* mRNA could be detected in both roots and leaves, and that of *NtNRT1.2/2.1/2.2* not in the leaves (Figure 3B, Figure S3B). In the roots, under normal N-supply (+N, 1 mM AN i.e., NH_4_NO_3_), *NtNRT1.1/1.2/2.1* but not *NtNRT2.2* were expressed in a relatively high amount (particularly for *NtNRT1.2/2.1* with a relative-expression level >15 and >20, respectively) (Figure 3B, Figure S3B); N-starvation (for 3 d) remarkably repressed the transcriptional level of *NtNRT1.1/1.2/2.1*, but strongly up-regulated *NtNRT2.2* expression (Figure 3B); Re-supply with ${\text{NH}}_{4}^{+}$ within 12 h caused a progressively transcriptional up-regulation of *NtNRT1.1* but a depression of *NtNRT1.2/2.1/2.2* transcription compared to that of 3-d N-starved control plants (Figure 3B); the presence of ${\text{NO}}_{3}^{-}$ for 4 h in the N-deprived medium induced more expression of *NtNRT1.1/1.2* but suppressed significantly the transcription of *NtNRT2.1/2.2* (Figure 3B); urea or Gln occurrence (for 4 h) seemed not to much affect *NRTs*' mRNA accumulation in the roots relative to that of 3-d N-starved control (Figure 3B).

In leaves, only *NtNRT1.1* expression was responsive to N treatments. As shown in Figure 3B, 3-d N-starvation induced about 3-fold higher level of relative-expression of *NtNRT1.1* compared to that of those grown with normal N supply; re-provision of 3-d N-starved plants with ${\text{NH}}_{4}^{+}$, ${\text{NO}}_{3}^{-}$, urea or Gln led to a transcriptional down-regulation to a level similar to or much lower than (see in the case of urea or Gln) that of the control (Figure 3B).

### The transcription of *NtAMTs* and *NtNRTs* displays differently a diurnal regulation pattern

To cognize if or how the transcription of putative *NtAMTs* and *NtNRTs* might be influenced by plant internal N-demand related to a carbon: nitrogen ratio (C:N ratio) or circadian clock, we monitored gene expression in a diurnal variation pattern by means of qPCR, which was conducted with whole RNA from roots or leaves of 3-week hydroponically-grown tobacco (K326) sampled over a 15 h light/9 h dark circle at the time point of 2:00, 6:00, 10:00, 14:00, and 20:00 (see section Materials and Methods).

**Figure 4:**
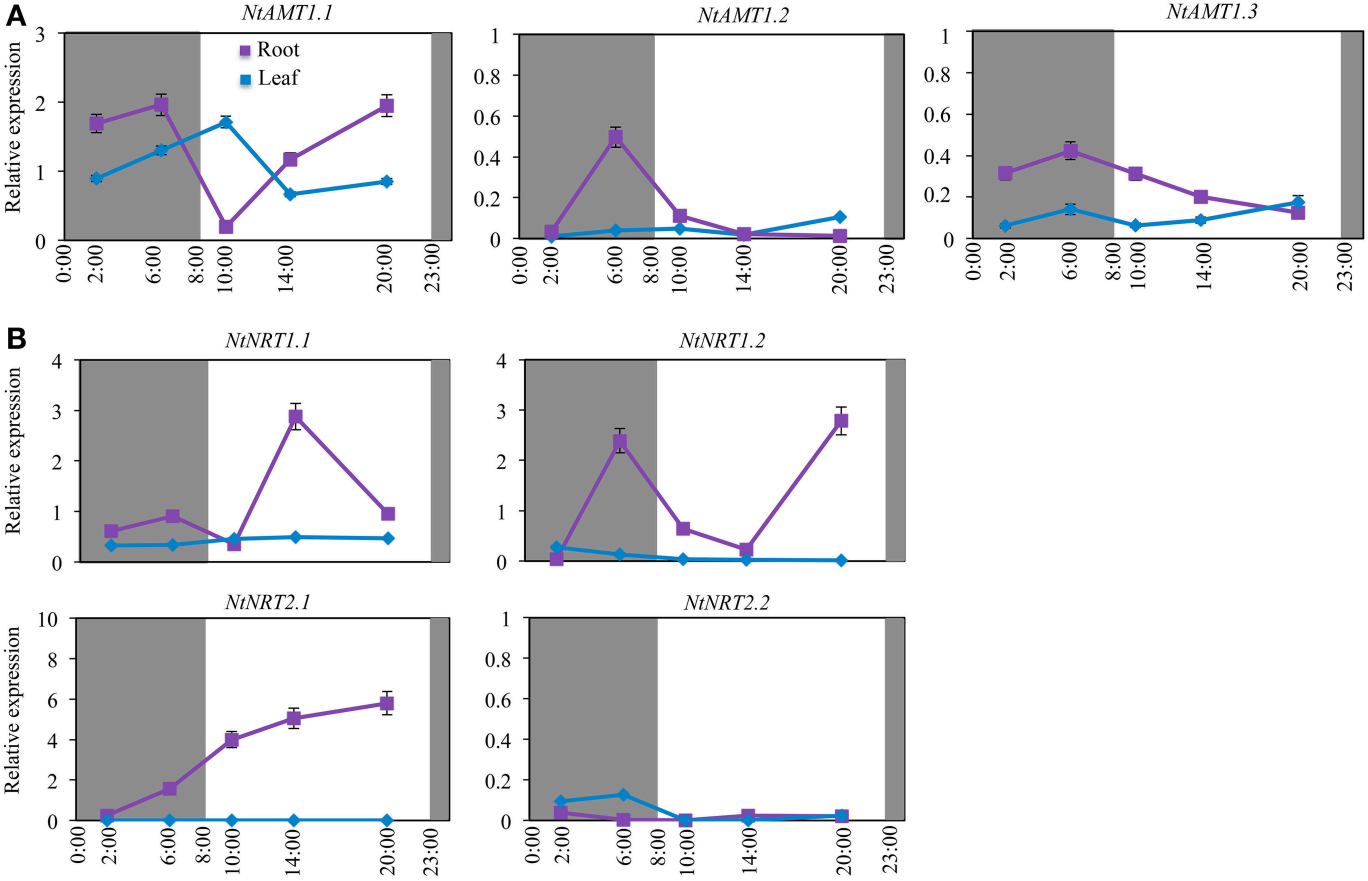
Diurnal-dependent expression of *AMTs* **(A)** and *NRTs* **(B)** in roots and leaves of tobacco k326. Plants were hydroponically cultured in a growth chamber with a 15 h light/9 h dark circle (08:00–23:00/23:00–8:00) (see section Materials and Methods). Plant samples were harvested at 2:00, 6:00, 10:00, 14:00, and 20:00. Relative mRNA accumulation of *NtAMTs* and *NtNRTs* was quantified by qPCR, which was conducted with total RNA from roots and leaves (1 d after fully-opened) of plants grown on normal nutrition solution for 3-week (see section Materials and Methods). Expression levels of *NtAMTs* and *NtNRTs* are relative to that of α*-tubulin* gene (set to 1). Mean values of 3–4 biological samples ± *SD* (*n* = 3–4) were shown. *L25* was used as a second reference gene to affirm the expression pattern observed from that using *tubulin* as an internal control (Figures S2E,F). Purple or blue lines indicate respectively root and leaf. The relative expression of those *NtAMT*s with a low level and less diurnal-variation pattern is shown in Figure S4.

Of nine *NtAMT*s, the expression of only *AMT1* members exhibited different diurnal-oscillation patterns (Figure 4A), whereas the transcript abundance of *AMT2.1, AMT3.1* and four *AMT4* members was detected at very low and relatively stable levels in both roots and leaves (also in the case of *AMT1.2/1.3*) during a diurnal circle (Figure S4; Figure 4A). In the roots, *NtAMT1.1* transcripts occurred with the highest amount at 6:00 (2 h before the onset of the light period) and then decreased to the lowest level at 10:00 (with 10-fold reduction), and thereafter elevated by 10-fold at 20:00 (3 h before dark) (Figure 4A); a higher transcription of *NtAMT1.2/1.3* was detected also at 6:00 and then continuously down-regulated over a period from 6:00 to 20:00 (Figure 4A). In the leaves, *NtAMT1.1* mRNA accumulation showed a diurnal expression pattern opposite to the expression in the roots (e.g., the highest vs. lowest at 10:00; Figure 4A).

With regard to the transcriptional regulation of *NtNRTs* by a diurnal change, as revealed in Figure 4B, the relative expression of all *NtNRTs* in leaves was in general fairly low and remained comparatively stable over a diurnal cycle (Figure 4B), while in roots the mRNA abundance of *NtNRT*s except for *NtNRT2.2* showed obvious fluctuations during the diurnal change (Figure 4B). Interestingly, the expression of both *NtNRT1.1* and *NtNRT1.2* exhibited an oscillation pattern but with a highest transcript accumulation shifted to different time points especially during the light period (Figure 4B), i.e., *NtNRT1.1* mRNA abundance peaked at 6:00 (but with a low level) and 14:00 (when *NtNRT1.2* expression was the lowest) (Figure 4B), and *NtNRT1.2* reached its maximum expression level at 6:00 and 20:00 (when *NtNRT1.1* expression was mostly repressed) (Figure 4B). A strong transcriptional up-regulation of *NtNRT1.2* in the scotophase (e.g., at 6:00) suggests that NtNRT1.2 would be an important pathway for uptake/transport of N (e.g., nitrate) in the roots, where a sufficient amount of N may be required for the assimilation of carbohydrate delivered from aerial part(s) in the dark. In the photophase, a reciprocal increase in mRNA abundance of *NtNRT1.1* and *NtNRT1.2* (Figure 4B), together with a preferential expression of *NtNRT2.1* in the daytime (Figure 4B), suggest that in tobacco at least two low-affinity and one high-affinity *NRTs* are differentially regulated by the circadian clock or at least a diurnal change, probably contributing co-ordinately to modulating plant internal N-demand via transport processes during a light-dark change.

### Impact of external sucrose and MSX on the expression of *NtAMTs* and *NtNRTs*

**Figure 5:**
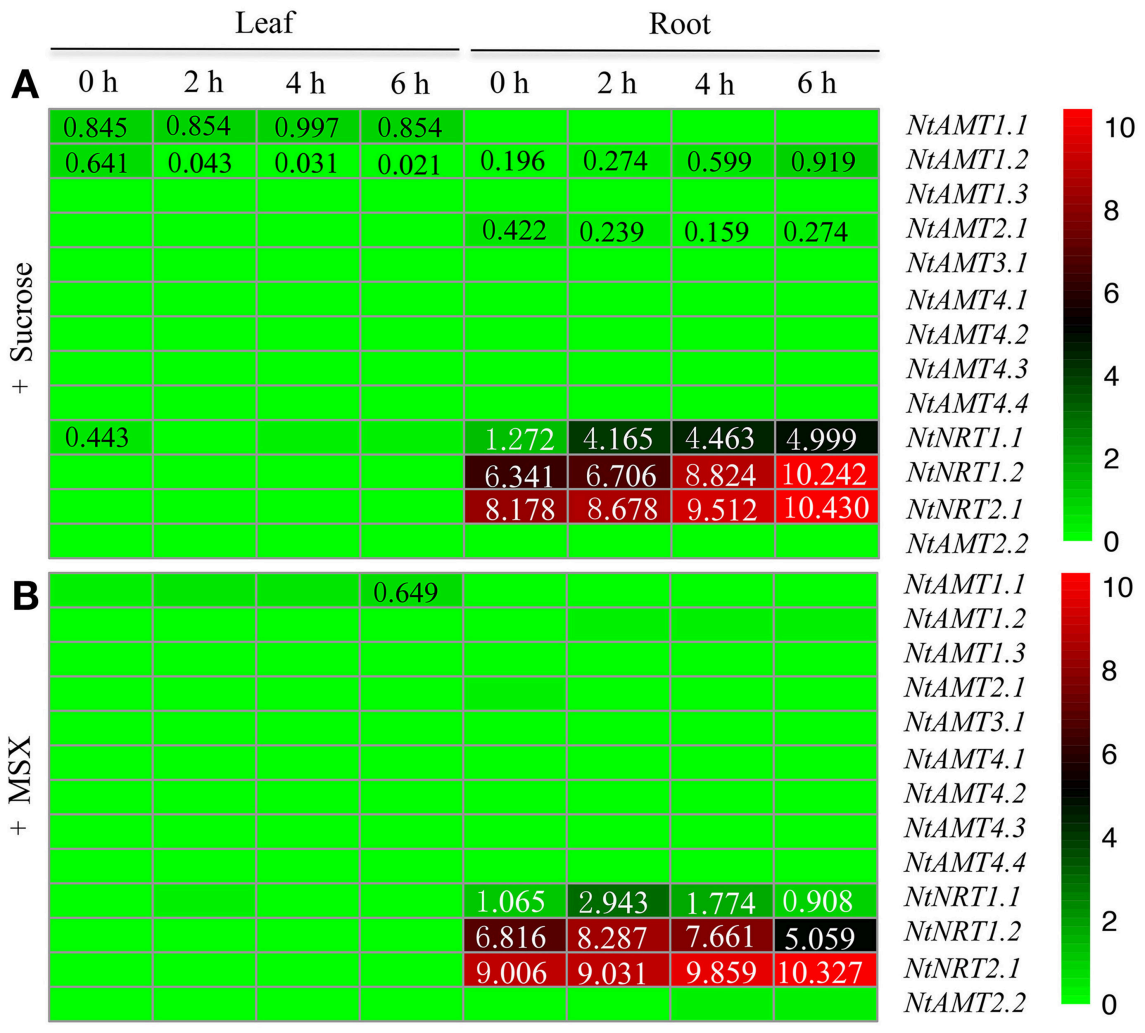
Heat map of transcriptional regulations of *AMTs* and *NRTs* in tobacco K326 by external sucrose and MSX. Plants were hydroponically grown (for 15 d) in normal nutrient solution and treated with 1% sucrose **(A)** or 1 mM MSX **(B)** for a time period of 0, 2, 4, or 6 h (see section Materials and Methods). Total RNA was extracted from roots and leaves (1 d after fully-opened); relative mRNA abundance of *NtAMTs* and *NtNRTs* was quantified by using qPCR, and the expression level of *AMTs* and *NRTs* relative to that of *L25* (set to 1) in each sample was calculated (see section Materials and Methods). A relative gene-expression abundance (derived from a difference of transcript levels between with and without MSX- or sucrose-treatment at each time point, except for time 0) was presented in a false color scale, where green or red color indicates respectively a lowest or highest expression with an absolute mean value at 0 or 10.

To discern if or how transcriptional response of tobacco *AMTs* and *NRTs* could be affected by plant N-assimilation and carbon-metabolism, gene expression was further monitored using qPCR on total RNA from 15-d hydroponically cultivated K326 plants, which were treated for different times with sucrose and MSX (methionine sulfoximine, an inhibitor of glutamine synthetase). As Figure 5 presented, in leaves, the expression of all identified *NtAMTs* and *NtNRTs* appeared not to be influenced obviously by the addition (0–6 h) of sucrose or MSX.

In roots, sucrose supply (after 6 h) could induce an increase in *NtAMT1.2* mRNA abundance (Figure 5A), while other *NtAMT* genes appeared not to transcriptionally respond to external sucrose (Figure 5A). Of four *NRTs, NtNRT1.1/1.2* were markedly up-regulated in the roots by the presence of sucrose (Figure 5A), an elevated transcript level was also observed for *NtNRT2.1* after 4–6 h sucrose induction (Figure 5A). These results may imply that the transport role or at least the transcriptional alteration of such *NtAMTs* and *NRTs* might link to carbon metabolism and/or sugar signaling in the roots, as reported for *AMTs/NRTs* from other species e.g., Arabidopsis and Populus (Gazzarrini et al., 1999; Rawat et al., 1999; Couturier et al., 2007), but this hypothesis related to sugar-regulated expression of tobacco *AMT1.2* and *NRT1.1, 1.2*, and *2.1* needs to be largely investigated in the future. Regarding MSX effect, exposure of the roots to this chemical for 2 h stimulated significantly higher expression of *NtNRT1.1* and *1.2* than that of in the control (i.e., 0 h MSX-treatment) (Figure 5B), however, these increased mRNA amounts were then declined by prolonged MSX-supply (e.g., after 6 h) (Figure 5B); 6-h incubation of the roots with MSX could increase obviously the quantity of *NtNRT2.1* transcripts as compared with the control (Figure 5B).

### Heterologous expression of *NtAMTs* and *NRTs* complemented mutant growth of yeast and arabidopsis

**Figure 6:**
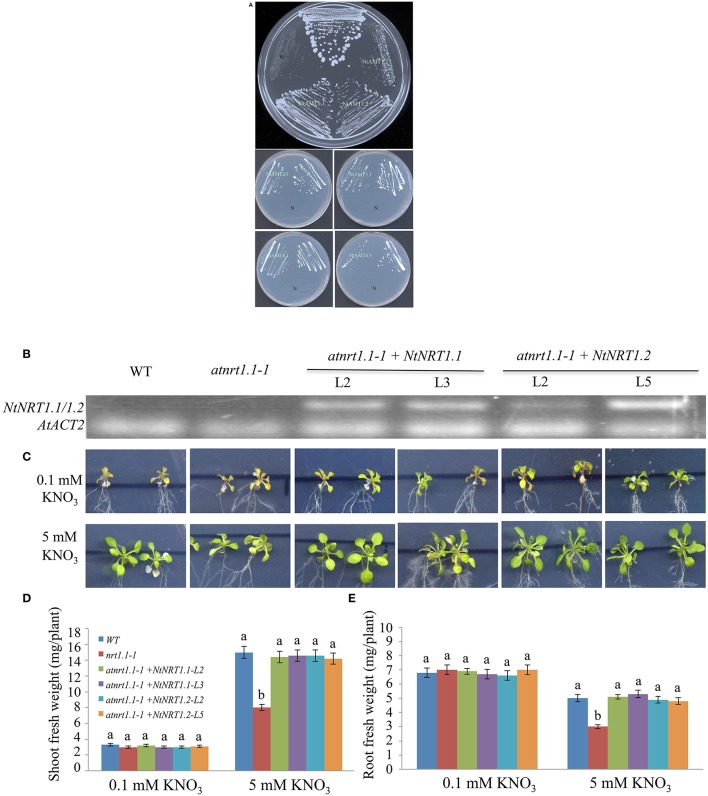
Growth complementation of a yeast or Arabidopsis mutant by heterologous expression of *NtAMTs* and *NtNRTs*. **(A)** Functional complementation of yeast mutant 31019b by the transformation of individual *NtAMTs*. The yeast strain 23346c (Δ*ura3*) and 31019b (Δ*mep1-3*, Δ*ura3*; defective in ${\text{NH}}_{4}^{+}$ uptake) transformed with a yeast expression-vector pHXT426 alone or harboring a putative ORF of *NtAMT1.1, 1.2, 1.3, 2.1, 3.1, 4.1*, and *4.3* were grown first on SD-medium agar plate containing 20 mM ${\text{NH}}_{4}^{+}$ as N source. A single colony was picked, suspended in 5 μl water and streaked onto the SD plate containing 2 mM ${\text{NH}}_{4}^{+}$ as sole N form. 31019b did not grow on <5 mM ${\text{NH}}_{4}^{+}$. Pictures were taken 5–6 days after yeast growth on ${\text{NH}}_{4}^{+}$ medium. P and N, positive and negative control (i.e., 23346c or 31019b harboring just pHXT426). **(B–E)** Expression of *NtNRT1.1/1.2* in an Arabidopsis mutant improved plant growth on nitrate. The putative ORF of *NtNRT1.1* or *NtNRT1.2* cloned after CaMV 35S-promoter was introduced into the Arabidopsis *atnrt1.1-1* line defected in *NRT1.1* gene (Hachiya et al., 2011), and two independent transgenic lines of *NtNRT1.1-* or *NtNRT1.2-*transformed *atnrt1.1* were used in the experiment. **(B)** Detection of gene expression in transgenic and non-transgenic (*atnrt1.1* and WT) plants. Semi-quantitative RT-PCR was conducted on total RNA from roots sampled from the experiment **(C)**. Primers for the amplification of *NtNRT1.1-* or *NtNRT1.2-* ORF were used (Table S3), and an Arabidopsis housekeeping gene *ACT2* served as a reference (Liu et al., 2003). The correctness of resulting amplicons was confirmed via DNA sequencing. **(C)** Growth phenotype of WT, *atnrt1.1-1, atnrt1.1-1*+*NtNRT1.1* (line 2 and 3) and *atnrt1.1-1* +*NtNRT1.2* (line 2 and 5). Plants were grown on 1/2 strength MS (N-free) agar-plate supplied with 0.1 or 5 mM KNO_3_ as only N source. Representative pictures were taken 10 days after plant growth on ${\text{NO}}_{3}^{-}$. Shoot **(D)** and root **(E)** biomass of WT, *atnrt1.1-1* and the transgenic lines. Data represent mean ± *SD* (*n* = 6 biological repeats, six plants in each), and different letters above the bars indicate statistically significant differences (*P* < 0.05 by one-way ANOVA and a multiple comparison test).

To know preliminarily about putative molecular action of identified tobacco *AMTs/NRTs* in ${\text{NH}}_{4}^{+}$ or ${\text{NO}}_{3}^{-}$ transport, heterologous functional-complementation approach was applied. The predicted ORF of *NtAMT1.1/1.2/1.3/2.1/3.1/4.1/4.3* and *NtNRT1.1/1.2* was cloned respectively into a yeast-expression vector pHXT426 and plant-expression plasmid pCF203 (carrying CaMV 35S promoter) (Wang W. H. et al., 2012) (see section Materials and Methods). The individual *NtAMTs* in the pHXT426 was then transformed into a yeast mutant 31019b (Δ*mep1-3*, Δ*ura3*), which is defective in ${\text{NH}}_{4}^{+}$ uptake and cannot growth on <5 mM ${\text{NH}}_{4}^{+}$ as a sole N source (Gazzarrini et al., 1999). Compared with transformants containing the empty vector pHXT426, yeast cells of 31019b harboring respectively *NtAMT1.1/1.2/1.3/2.1/3.1/4.1/4.3* restored the growth on 2 mM ${\text{NH}}_{4}^{+}$, comparable to that of the wild-type strain 23346c (Δ*ura3*) transformed with pHXT426 (Figure 6A), indicating that these *NtAMTs* ORF-encoded proteins could facilitate ${\text{NH}}_{4}^{+}$ movement from medium across the plasma membrane of yeast cells.

By transformation of *NtNRT1.1* or *NtNRT1.2* into an Arabidopsis mutant (*atnrt1.1-1*), which is deleted in a nitrate transporter *NRT1.1* and grows abnormally (e.g., smaller and more yellow) with ${\text{NO}}_{3}^{-}$ as sole N source (at <5 mM; Hachiya et al., 2011), some independent *NtAMT1.1-/1.2*-harboring homozygous lines were generated (see section Materials and Methods). As shown in Figure 6B, the actual expression of *NtNRT1.1*/*1.2* was confirmed by semi-quantitative PCR in transgenic plants, which were grown for 10 d on half strength MS (N-free) agar-plate supplied with 0.1 or 5 mM ${\text{NO}}_{3}^{-}$ as N-source after 5 d pre-culture on 1 mM NH_4_NO_3_ (see section Materials and Methods). Growth phenotyping revealed that the overexpression of *NtNRT1.1*/*1.2* could obviously improve *atnrt1.1* growth on ${\text{NO}}_{3}^{-}$ (Figure 6C), with characteristics of a bigger plant size, generously greener leaves and a significantly higher biomass as compared to that of the mutant (Figures 6C–E), leading to a suggestion of a possible physiological relevance of *NtNRT1.1*/*1.2* in plant ${\text{NO}}_{3}^{-}$ acquisition and utilization, but bona fide functions of these transporters in plant nitrate homeostatis in tobacco remains to be elucidated.

## Discussion

Although biological functions of *AMT* and *NRT* genes were widely studied in several plant species e.g., Arabidopsis, wheat, and rice etc. (Gazzarrini et al., 1999; Plett et al., 2010; Buchner and Hawkesford, 2014), genetic bases underlying transport processes for ${\text{NH}}_{4}^{+}$ and ${\text{NO}}_{3}^{-}$ in tobacco are poorly known. Here, we reported for the first time the identification of putative sequences coding for 9 AMTs and 4 NRTs from tobacco. Our results gained from the expression profiling of these genes in different tissues and growth conditions as well as heterologous functional complementation provide an overall figure, which should be informative and valuable for further understanding of their physiological significance for N nutrition in tobacco growth.

### Evolutionary conservation and divergence of NtAMTs and NtNRTs

To date, the existence of homologs of AMTs and NRTs in some plant species has been documented in many publications (Koegel et al., 2013; von Wittgenstein et al., 2014; O'Brien et al., 2016). Using homology BLAST search against the annotated genome at Sol Genomics Network allows us to identify indeed in tobacco (*Nt* L. cv. K326) at least 13 putative coding sequences, 9 of which belong to *AMT* gene family and 4 to *NRT* family (Table 1; Figure 1; Table S4), emphasizing a notion that such orthologs for AMT and NRT proteins should be evolutionarily conserved throughout *planta*. In tobacco database, presence of corresponding EST's confirms that the identified genes are indeed expressed (Table 1).

Based on the sequence similarity, 9 *NtAMT* genes can be classified into four clusters, namely *AMT1* (3 members), *AMT2* (1), *AMT3* (1), and *AMT4* (4) (Figures 1A,B). Compared with Arabidopsis, sorghum and rice, tobacco contains also similar numbers of *AMTs*, e.g., 6 in Arabidopsis, 8 in sorghum, and 10 in rice. However, their assignment to individual subclasses appears different, for instance, tobacco possesses a smaller number (i.e., only 1) in the *AMT3* subfamily than in the rice (3 members) and sorghum (3 members) (Figures 1A,B); in contrast, four members fall into the subgroup *AMT4* in tobacco but only one in sorghum (Koegel et al., 2013) (Figure 1B). These different gene numbers might indicate distinct duplications of *AMT* genes after the divergence of monocots and eudicots. In addition, most genes in the *AMT1* cluster comprise only one exon, except for *PtrAMT1.7, LjAMT1.1*, and *NtAMT1.2* with their ORFs spliced from one intron (Wu et al., 2015) (Table 1).

For nitrate transporters, all of *Arabidopsis* NRTs, for which four tobacco homologs were identified here, are able to transport ${\text{NO}}_{3}^{-}$ when expressed in *Xenopus* oocyte (Orsel et al., 2002; Tsay et al., 2007). Given a background knowledge that orthologous genes from e.g., Arabidopsis and rice involve ${\text{NO}}_{3}^{-}$ root-uptake and transport within the plant (von Wittgenstein et al., 2014), a phylogenetically close relationship might point to a similar function and substrate transport-specificity for tobacco orthologous gene products. Notably, compared with other species, NtNRT1.1/1.2 share higher similarity (66.3% sequence identity) to AtNRT1.1 (Table S1B), proposing that NtNRT1.1/1.2 would fulfill a similar function for ${\text{NO}}_{3}^{-}$ permeation into tobacco cells. To test such a hypothesis, a future work will be addressed on molecular and physiological characterization of NtNRTs in ${\text{NO}}_{3}^{-}$ movement process. Besides 4 *NtNRTs* studied here, one could not exclude that there might be more NRT homologs in the tobacco genome, since this family comprises relatively large members as reported for Arabidopsis and rice etc. (Plett et al., 2010). Therefore, the *NRT* family members suggest that not more than the four *NRT's* characterized here in tobacco are known.

More significantly, heterologous expression of the putative ORF of seven *NtAMTs* (*AMT1.1/1.2/1.3/2.1/3.1/4.1/4.3*) and two *NtNRTs* (*NRT1.1/1.2*) successfully complemented the yeast ${\text{NH}}_{4}^{+}$-uptake mutant or Arabidopsis ${\text{NO}}_{3}^{-}$ transporter mutant (Yuan et al., 2007; Hachiya et al., 2011), suggesting functionality of transport activity *in vivo*.

### Complexity of *NtAMTs'* and *NtNRTs'* expressions in different aerial tissues

To ensure their normal growth and development, plants express their functional genes mostly in a spatiotemporal manner (Plett et al., 2010; Feng et al., 2011). For tobacco, its (leaf) yield and quality depend largely on the active exchange of metabolites and mineral nutrients including particularly N between aged (source) and developing (sink) organs (especially leaves) (Masclaux et al., 2000). Although metabolic events were intensively studied in this sink-source transition, a possible role(s) for *NtAMT* and *NtNRT* genes in N transport/nutrition in differently aged leaves as well as in other N-sink tissues (e.g., flowers) is little understood.

In this work, 3 members from *NtAMT1*-family were isolated in tobacco. Tissue-specific gene expression assay indicated that *NtAMT1.1* was widely expressed in leaves, flowers, and roots (Figures 2, 3A), similar to that of *AtAMT1.1* (Neuhäuser et al., 2007). A strong expression of *NtAMT1.1/2.1* and *NtNRT1.2/2.2* in the old leaves might reflect their dominant roles in N remobilization from the source tissues/organs once required (Figure 2C); likewise, because of their relatively higher expression in young and mature leaves (Figures 2A,B), NtAMT1.1 and NtNRT1.2 might also contribute mostly to ${\text{NH}}_{4}^{+}$ and ${\text{NO}}_{3}^{-}$ distribution into sink tissues.

For *AMT2* subcluster, similar to the case in Arabidopsis (Sohlenkamp et al., 2002), we revealed in tobacco also only one *AMT2* member *NtAMT2.1*, with its higher expression level in aged leaves than in young and mature ones (Figures 2A–C), leading to a suggestion that NtAMT2.1 might be induced by senescence process associated with ammonium-N redistribution. Regarding *AMT3* and *AMT4* genes, their differently tissue-specific transcript abundance was mostly dependent of plant species, e.g., poplar *PtrAMT3.1* with a maximal expression in male catkins was induced during senescence, *PtrAMT4.3* or *PtrAMT4.4* was leaf- or stem-specifically transcribed, respectively (Wu et al., 2015); rice *OsAMT3.1* has an highest expression in seeds (von Wittgenstein et al., 2014). However, in the case of tobacco grown under pot-soil conditions, transcripts of most identified *NtAMT3* and *NtAMT4* members was detectable but at very low levels in aerial parts (Figure 2, Figure S3A).

Regarding *NRT*s, their distinct tissue-specific expressions were well-described for certain plants (Fan et al., 2009; Migocka et al., 2013), suggesting diverse biological functions of *NRT1* and *NRT2* in specific tissues. Molecularly, Arabidopsis *NRT1.1* was most extensively studied. AtNRT1.1 was shown to fulfill multiple functions (e.g., as a ${\text{NO}}_{3}^{-}$ transporter and sensor in the roots), and occurred widely in shoots, roots and with highest expression in the epidermis of the primary root tip (Nazoa et al., 2003). Similar to *AtNRT1.1*, tobacco *NtNRT1.1* mRNA presents also widespread in the roots, leaves and flowers (Figure 3B; Figure S3A). However, the highest expression of *NtNRT1.1* was in the mature leaves (Figure 2B), implying its complex biological roles in tobacco. The expression of *NtNRT2.1* seemed to be calyx-specific amongst aerial part tissues (Figure 2E). The transcripts of *NtNRT1.2* and *NtNRT2.2* were measured mostly in aged leaves (Figure 2C), assuming that they might involve the re-distribution and -utilization of nitrate-N from senescing parts to growing tissues, as reported for NRT1.7 in Arabidopsis (Fan et al., 2009).

### Transcriptional regulation of *NtAMTs* and *NtNRTs* by N-nutritional status

Transcriptional regulation of membrane permeases for the transport of nutrients/solutes is thought to be a common characteristic of plant response to the nutritional status and/or their substrates (Gazzarrini et al., 1999; Liu et al., 2015). For instance, in rice roots, both *OsAMT1.1* and *OsAMT1.2* expression were ${\text{NH}}_{4}^{+}$ inducible in N-starved plants, and repressed by transfer from low to high ${\text{NH}}_{4}^{+}$, while *OsAMT1.3* was changed slightly (Kumar et al., 2003); *OsNRT2.1/2.2*/*2.3a* were up-regulated by ${\text{NO}}_{3}^{-}$ and suppressed by ${\text{NH}}_{4}^{+}$ (Feng et al., 2011). In this study, the expression of *NtAMTs* /*NRTs* responsive to N-depletion and re-provision with ${\text{NH}}_{4}^{+}$, ${\text{NO}}_{3}^{-}$, urea and glutamine exhibited similar and complex patterns (Figures 3A,B; Figure S3B), as reported for their counterparts in Arabidopsis and rice etc. (Gazzarrini et al., 1999; Feng et al., 2011), leading to a suggestion of an individual fine tuning of the regulation of *NtAMTs*/*NRTs* in respect of N acquisition, transport/remobilization as well as even sensing. Notably, the expression of *NtAMT1.1* in the roots could be markedly up-regulated by both N-starvation and resupply with ${\text{NH}}_{4}^{+}$, ${\text{NO}}_{3}^{-}$ urea and glutamine after N-starvation (Figure 3A), different from Arabidopsis *AMT1.1* that was transcriptionally repressed by ${\text{NH}}_{4}^{+}$ and ${\text{NO}}_{3}^{-}$ due to perhaps a different feedback inhibition (Gansel et al., 2001), proposing that NtAMT1.1 would play a dominant role in ${\text{NH}}_{4}^{+}$-N transport whenever required in the tobacco roots. A tendency of *NtAMT2.1* root-expression responsive to N-starvation and resupply with ${\text{NH}}_{4}^{+}$ and ${\text{NO}}_{3}^{-}$ appears similar to that of measured for *NtAMT1.1* (Figure 3A), although overall levels of their mRNA accumulation were much different (i.e., with about 10-fold higher for *NtAMT1.1* than *NtAMT2.1*. Figure 3A). Interestingly, the expression of *NtAMT1.2, 1.3, 3.1, 4.1, 4.2, 4.3*, and *4.4* in response to N could be detected most obviously only in leaves (Figure 3A), implying that the leaf would be a major site for such *NtAMTs* with their functions linked to plant N-status and (${\text{NH}}_{4}^{+}$-) N movement within aerial part tissues. Nevertheless, how such individual NtAMTs implement their molecular and physiological roles related to N-nutrition in the tobacco remains interesting in our future study.

Concerning *NRT* homologs, their transcriptional regulations were well-documented to be associated with plant N-nutritional status (Okamoto et al., 2003; Feng et al., 2011). Indeed, the mRNA abundance of *NtNRT1.1* was regulated by varied N treatments in roots and leaves, and expressions of *NtNRT1.2***,** *2.1* and *2.2* responsive to N were most likely root-specific (Figure 3B; Figure S3B), suggesting the significance of such NtNRTs in ${\text{NO}}_{3}^{-}$ uptake/transport and even sensing in tobacco root system. Transcriptionally, under N-deficient condition, only *NtNRT2.2* was shown an increase in its mRNA abundance in the roots (Figure 3B), indicating that NtNRT2.2 would be a critical component positively regulating N demand of tobacco when subjected to N limitation. Notably, similar to *AtNRT1.1, 2.1*, and *2.2* (Okamoto et al., 2003), only *NtNRT1.2* in the roots was measured to be strongly induced by ${\text{NO}}_{3}^{-}$ after N-starvation, with a highest level occurred after 4 h ${\text{NO}}_{3}^{-}$ resupply (Figure 3B), proposing that NtNRT1.2 should be a major molecular factor with a substrate-inducible nature for root ${\text{NO}}_{3}^{-}$ uptake (Figure 3B). In addition, external ${\text{NH}}_{4}^{+}$ suppressed the expression of *AtNRT1.1, 2.1*, and *2.2* but stimulated *NtNRT1.1* transcription (Figure 3B) after medium N-depletion, speculating that these *NtNRT* genes might be differently regulated by a N-metabolic feedback signal(s), as reported for rice *OsNRT2.1/2.2* (Feng et al., 2011). Further, these different metabolic-feedback regulations of *NtNRTs* in the roots could be partly supported by MSX-affected gene expression study, where a remarkably higher transcription of *NtNRT1.1* and *1.2* was stimulated by 2 h MSX supply and then declined after prolonged MSX treatment (Figure 5B), and an obvious up-regulation of *NtNRT1.2* and *2.1* occurred only after 6 h root-exposure to MSX (Figure 5B). Nevertheless, our results from N-dependent gene expression analysis should argue a notion that an intricate transcriptional-regulation of identified *NtAMT*s and *NtNRT*s might reflect a possibility and also ability of tobacco to adapt to a fluctuation of N in internal and soil environments.

### Effect of a photoperiod on transcriptional regulation of *NtAMTs* and *NtNRTs*

Studies with Arabidopsis, rice, and tomato reported a diurnal change of the uptake and assimilation of ${\text{NH}}_{4}^{+}$ and ${\text{NO}}_{3}^{-}$ (Gazzarrini et al., 1999; Lejay et al., 1999; Feng et al., 2011). Physiologically, this type of the variation appears to depend closely upon certain events involved in e.g., a status of N and C in roots, circadian clock and N assimilation. Here, we observed a different effect of a light-dark cycle on the transcript abundance of tobacco *AMTs* and *NRT*s. As sown in Figure 4, of 9 *NtAMT*s and 4 *NtNRTs*, expressions of *NtAMT1.1/1.2/1.3* and *NtNRT1.1/1.2/2.1* were markedly regulated by the diurnal cycle (Figures 4A,B), but the rest not (Figure S4). Despite a closely phylogenetic relation of *NtAMT1.1/1.2/1.3* to their counterparts in tomato (von Wirén et al., 2000), their diurnal regulation patterns are somehow distinct. Unlike *LeAMT1.1* with its transcript accumulation at a constantly low level in leaves (von Wirén et al., 2000), *NtAMT1.1* was expressed well in both roots and leaves, with a maximum transcript level observed 2 h after the onset of the light in the leaves but the highest mRNA abundance in the roots occurred 2 h before the light onset and a second peak at the end of light (or 3 h before dark) (Figure 4A), assuming that this differentially diurnal regulation of *NtAMT1.1* might reflect a difference of N-demand or -status between the roots and leaves because of circadian-dependent production and distribution of carbon-skeleton in these two tissues (Gazzarrini et al., 1999). Regarding to *NtAMT1.2* and *NtAMT1.3*, though their mRNA was ${\text{NH}}_{4}^{+}$-inducible (Figure 3A), similar to that of tomato *LeAMT1.2* and *1.3* (von Wirén et al., 2000), their diurnal expression undulation was different. In tomato, the expression of *LeAMT1.2* and *1.3* exhibited a reciprocal pattern (von Wirén et al., 2000), which was not revealed for *NtAMT1.2* and *1.3* (Figure 4A). These results lead to a suggestion that, besides NtAMT1.1 being a major ${\text{NH}}_{4}^{+}$ transporter in darkness (since its maximum root-expression occurred 2 h before light, Figure 4A), NtAMT1.2 and 1.3 would be additional molecular components required for an enhancement of ${\text{NH}}_{4}^{+}$-N acquisition into the roots, where an internal limitation of N might become prominent due to its intensive incorporation into carbohydrates, which are delivered from aerial parts during the dark period. This hypothesis can be supported by at least in part the observation of N-starvation stimulated up-regulation of *NtAMT1.1, 1.2*, and *1.3* in the roots (Figure 3A). However, surprisingly, unlike *NtNRTs* (Figure 5A), the expression of these *NtAMT1* members seemed not to be obviously influenced by external root-supply of sucrose (Figure 5A), perhaps due to that the time (0–6 h) with sucrose treatment would not be long enough to cause an internal C- and/or N-status/metabolic change to a certain extent required for regulating *NtAMT1*s' transcription, or due to a low sensitivity of their expression response to medium sucrose.

The diurnal regulation of *NRTs*' expression was described also in certain plant species including Arabidopsis, potato and phytoplankton (Tai and Zebarth, 2015). For instance, mRNA levels of Arabidopsis *NRT1.1* and *NRT2.1* in roots were regulated by a marked diurnal-change and quickly increased by sucrose supply at night (Lejay et al., 1999). In this work, the relative expression of 4 identified *NtNRTs* in tobacco leaves was detected at fairly low and comparatively stable levels over a diurnal period (Figure S4), coincident with a previous observation of no significant diurnal-oscillation for nitrate level in leaves of *N. tabacum* (Poire et al., 2010). In the roots, 4 *NtNRTs* except for *NtNRT2.2* at a transcriptional level were shown differential diurnal oscillations (Figure 4B). Similar to *NRT2* genes in Arabidopsis and other plant species, the expression level of *NtNRT1.2* and *NtNRT2.1* peaked at the end of lighting likely due to the accumulation of carbon from shoots (Rawat et al., 1999; Couturier et al., 2007; Feng et al., 2011). mRNA abundance of *NtNRT1.1* and *1.2* exhibited similar but a desynchronized oscillation fashion with the highest transcript accumulation peak shifting to different time points of a diurnal circle (Figure 4B), arguing for a notion that such different expression fluctuations might be induced by internal circadian-clock signal(s), temperature, carbon and/or N status (Poire et al., 2010; Feng et al., 2011). This suggestion can be at least partly supported by our observation that a short time application of both sucrose and MSX could elicit expression changes of *NtNRT1.1, 1.2*, and *2.1* (Figure 5).

In conclusion, from public database we identified and isolated for the first time from tobacco nine and four coding sequences respectively for ${\text{NH}}_{4}^{+}$- and ${\text{NO}}_{3}^{-}$-transporters (*NtAMTs, NtNRTs*), most of whose transport activities were preliminarily evidenced by heterologous functional complementation test. Moreover, our data from carefully gene expression-profiling by qPCR revealed obviously that: (i) A strong expression of *NtAMT1.1, 1.3*, and *2.1, NtNRT1.2* and *2.2* in aged leaves might reflect their major roles in N remobilization from source tissues once required; NtAMT1.1 and NtNRT1.1 could contribute mostly to ${\text{NH}}_{4}^{+}$ and ${\text{NO}}_{3}^{-}$ distribution into sink tissues. (ii) Upregulation in roots of *NtAMT1.1* by both N-starvation and resupply with N including ${\text{NH}}_{4}^{+}$ suggests a primary role of NtAMT1.1 in ${\text{NH}}_{4}^{+}$ uptake/transport whenever needed in the roots, whereas the obvious expression in leaves of other *NtAMTs* responsive to external N implies a major site for such NtAMTs with their functions associated with plant N-status and N movement within aerial-part tissues; preferentially root-specific transcriptions of *NtNRT1.1, 1.2*, and *2.1* in response to N strongly argue the importance of these NtNRTs in ${\text{NO}}_{3}^{-}$ uptake and even sensing in tobacco roots. (iii) Only *NtAMT1.1, NtNRT1.1* and *1.2* were markedly shown their expression alteration in a typical diurnal-oscillation pattern particularly in the roots, reflecting perhaps their significant actions in root N acquisition regulated by an internal N-demand or -status. Thus, this work should provide not only experimental evidence for the existence of different tobacco *AMT*/*NRT* genes, but also valuable molecular information for further understanding mechanisms involved in tobacco N transport and utilization contributed by individual *AMTs* and *NRTs*.

## Author contributions

L-HL: Designed the experiments; L-HL, T-FF, D-XS, and C-JL: Conducted major experiments and data analysis. Other authors assisted in certain measurements, analyzed data, and discussed the results. L-HL and T-FF prepared the manuscript.

### Conflict of interest statement

The authors declare that the research was conducted in the absence of any commercial or financial relationships that could be construed as a potential conflict of interest.
