# Supplementary material for: Coding-Sequence Identification and Transcriptional Profiling of Nine AMTs and Four NRTs From Tobacco Revealed Their Differential Regulation by Developmental Stages, Nitrogen Nutrition, and Photoperiod
Source: Front Plant Sci. 2018 Mar 5;9:210. doi: 10.3389/fpls.2018.00210 (PMC5850829; doi:10.3389/fpls.2018.00210)
Supplement: Supplementary file 2 [file Table2.PDF]

Table S2 Accession numbers of AMT and NRT proteins extracted from GenBanks and used for the phylogenetic analysis.

AMTs:

Arabidopsis thaliana in NCBI:
AtAMT1.1 (NP_193087.1), AtAMT1.2 (AEE34288.1), AtAMT1.3 (AEE76886), AtAMT1.4 (At4g28700), AtAMT1.5 (AEE76885.1), AtAMT2.1 (NP_181363)

Lycopersicon esculentum in NCBI:
LeAMT1.1 (NP_001304667.1), LeAMT1.2 (NP_001234253.2), LeAMT1.3 (NP_001234216.1)

Lotus japonicas in GenBank:
LjAMT1.2 (AAM95453), LjAMT1.1 (CAC10555.1), LjAMT1.3 (CAE01484), LjAMT2.1 (AAL08212.1)

Nicotiana tabacum in GenBank:
NtAMT1.1 (XP_009784484.1), NtAMT1.2 (XP_009795683.1), NtAMT1.3 (XP_009768027.1), NtAMT2.1 (XP_009778221.1), NtAMT3.1(XP_009800812.1), NtAMT4.1(XP_009799644.1), NtAMT4.2(XP_009784746.1), NtAMT4.3(XP_009796398.1), NtAMT4.4(XP_009765535.1)

Oryza sativa in GenBank:
OsAMT1.1 (CAE03364.1), OsAMT1.2 (BAD21532.1), OsAMT1.3 (BAD21574.1) OsAMT2.1 (BAC65231.1), OsAMT2.2 (CAY33634.1), OsAMT2.3 (NP_915334.1) OsAMT3.1 (BAD33268.1), OsAMT3.2 (BAD33268.1), OsAMT3.3 (AAO41130.1), OsAMT4.1 (Q10CV4.1)

Populus trichocarpa in UniProt:
PtrAMT1.1 (B9HSW3), PtrAMT1.2 (B9IPE2), PtrAMT1.3 (B9HKW8), PtrAMT1.4 (B9GRB5), PtrAMT1.5 (B9GRB4), PtrAMT1.6 (B9HP47), PtrAMT2.1 (B9HCZ0), PtrAMT2.2 (B9IGE2), PtrAMT3.1 (B9GHA5), PtrAMT4.1 (B9GS88), PtrAMT4.2 (B9IKS2), PtrAMT4.3 (B9H8E7), PtrAMT4.4 (B9I5F0)

Sorghum bicolor in NCBI:
SbAMT1;1 (XP_002446777), SbAMT1;2 (XP_002452468.1), SbAMT2;1 (XP_002439939.1), SbAMT2;2 (XP_002458715.1), SbAMT3;1 (XP_002456706.1), SbAMT3;2 (XP_002466132.1), SbAMT3;3 (XP_002452249.1), SbAMT4 (XP_002466457.1)

Triticum aestivum in GenBank:
TaAMT1.1 (AAS19466), TaAMT2.1 (AAR87397)

Saccharomyces cerevisiae MEPs in Genbank:

ScMEP 1 (CAA97132.1), ScMEP2 (CAA96025.1)

NRTs:
Arabidopsis thaliana in NCBI:
AtNRT1.1 (NP_563899.1), AtNRT1.2 (NP_564978.1), AtNRT2.1 (NP_172288.1), AtNRT2.2 (NP_172289.1)

Oryza sativa in GenBank:
OsNRT1.1 (ABF94839.1), OsNRT2.1 (BAA33382)

Hordeum vulgare in GenBank:
HvNRT2.1 (AAC49531)

Triticum aestivum in GenBank:
TaNRT1.1 (AAT69243.1), TaNRT2.1 (AAG01172.1)

Lycopersicon esculentum in NCBI:
LeNRT1 (NP_001307053.1), LeNRT2.3 (NP_001234127.1)

Nicotiana tabacum in GenBank:
NtNRT1.1 (BAC56914.1), NtNRT1.2 (BAC56915.1), NtNRT2.1 (CAD89798.1), NtNRT2.2 (CAD89799.1)

Escherichia coli in GenBank:
NARK (CAA34126)

Hansenula polymorpha in GenBank:
YNT1 (CAA93631)
