# Supplementary material for: Coding-Sequence Identification and Transcriptional Profiling of Nine AMTs and Four NRTs From Tobacco Revealed Their Differential Regulation by Developmental Stages, Nitrogen Nutrition, and Photoperiod
Source: Front Plant Sci. 2018 Mar 5;9:210. doi: 10.3389/fpls.2018.00210 (PMC5850829; doi:10.3389/fpls.2018.00210)

Table S3 List of primers used for gene cloning and qPCR-based gene expression analysis of *AMT* and *NRT* genes in tobacco (*Nicotiana tabacum* L. cv. K326)

Gene	Amplicon length of qPCR (bp)	Primer for qPCR	Primer for gene cloning
NtAMT1.1	164	5′-GATCCACTTGAAGCAGCACA-3′<br>5′-TGTGCCCCAAGTAGTTTTCC-3′	5′-GAggatccATGGCTTGTGAAGTTAACC-3′<br>5′-ATggatccCTAACTTGAGCTTGTTGG-3′
NtAMT1.2	175	5′-CCATCTTTTGACTACAGCTACT-3′<br>5′-CGTCACCGGACCAGAACCAA-3′	5′-ttggatcc ATGGCCTCAGCCGTGACCTGCT-3′<br>5′-ATggatcc TTACACAACCACAGATCTAGA3′
NtAMT1.3	168	5′-CATAGCTGAACGTACCCAAT-3′<br>5′-CCACTGCCAGCAAAGTCAAT-3′	5′-GTggatccATGGATACTTCATGGGAA-3′<br>5′-GGggatccTTATGATTGGTGTTGCATC-3′
NtAMT2.1	197	5′-GCTTTTGTTCCTCTTTGGCT-3′<br>5′-GGTGGAAACCTTTCCCTATCA-3′	5′-CtggatccATGTCCATACCAGGAGCTT-3′<br>5′-GtggatccTTATAAATTAATTGTTACTCCTC-3′
NtAMT3.1	152	5′-TAACTCTGCTTTCATGGCTC-3′<br>5′-CGGCTTGTTTGATGAGGAAC-3′	5′-ATggatccATGGATACAGTGGTGCCACAG-3′<br>5′-TTggatccTTATATCATTTCTGTAGTCCCTC-3′
NtAMT4.1	204	5′-GCATGGATGTTGTTTGTTCC–3′<br>5′-GTTTGGTGGGAAATGTTGTC-3′	5′-TTagatctATGGATCCAAATAATTCATACCTTC-3′<br>5′-AGtctagaGATAACTCAACAAGGTATAACCG-3′
NtAMT4.2	160	5′-GGAGCATACACCATTTGGTC-3′<br>5′-GTGGAAATCTCTCTCTGTCC-3′	5′-TGggatccATGAGTTTATTAGCTCCACC-3′<br>5′-TAggatccTTAGACCATTTCAACCTGAC-3′
NtAMT4.3	137	5′-GCTCACTTGGCTAATGTTAG-3′<br>5′-CCATTAGTATTGCTGCCCAAC-3′	5′-TAggatccATGGCATACTTGCCTAGAAACC-3′<br>5′-CAggatccTCAAAGTCCATAGCCACTTTTGG-3′
NtAMT4.4	99	5′-CTTCTGGTGTAGCTGGTTTC-3′<br>5′-CCAGCATCATCAGAATGTTG-3′	5′-TAggatccATGGAACTTCCCTCAAATC-3′<br>5′-AGggatccTCATACCATTTGGAATTCAC-3′
NtNRT1.1	204	5′-GTGGTTTCGTAGCCGACACT-3′<br>5′-CCGTCATGTATAGTGCTATGT-3′	5′-CGggatccATGGCACTTCCTGAAACACA-3′<br>5′-CGggatccTTAGTGGCAAGCTGGTTCTG-3′
NtNRT1.2	229	5′-GGTATCTTTGCCACTGTTCA-3′<br>5′-CAGCATCGTCGAATTGGTCG-3′	5′-TTggatccATGGCACTTCCTGAGACA-3′<br>5′-ATggatccTCAATGACAAACCGGTCCA-3′
NtNRT2.1	140	5′-CTATTGGTGCTCAAGCTGCA-3′<br>5′-CGTGAAGAACAACAGTTGT-3′	5′-AGggatccATGGGTGATATTGAGGGCGAA-3′<br>5′-TGggatccTCAGACACGATTAGGCGTTA-3′
NtNRT2.2	171	5′-GATATTGTGGTATGCTGCAACA-3′<br>5′-GCCATACCAAATGTGGCAGCA-3′	5′-AGggatccATGGTTGATATTGAAGGATC-3′<br>5′-TGggatccTCAGACACGGTTTGGCGTT-3′
α-Tubulin	196	5′-GGTATTCAGGTCGGAAATGCA-3′<br>5′-CTTCGTCAATGACAGTAGGCT-3′	
L25	51	5′-CCCCTCACCACAGAGTCTGC-3′<br>5′-AAGGGTGTTGTTGTCCTCAATCTT-3′	

* Semi-quantitative RT-PCR-based specificity test of primer pairs used in qPCR analysis (Note: Method described as in Liu et al., 2003).

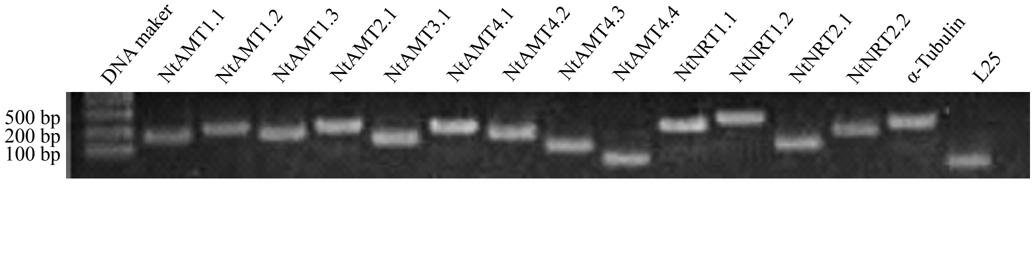

Supplement: Supplementary file 3 [file Table3.PDF]
