# Supplementary material for: Coding-Sequence Identification and Transcriptional Profiling of Nine AMTs and Four NRTs From Tobacco Revealed Their Differential Regulation by Developmental Stages, Nitrogen Nutrition, and Photoperiod
Source: Front Plant Sci. 2018 Mar 5;9:210. doi: 10.3389/fpls.2018.00210 (PMC5850829; doi:10.3389/fpls.2018.00210)
Supplement: Supplementary file 4 [file Table4.PDF]

Table S4 Protein sequence with its accession number used for the construction of phylogenetic tree

NRTs:

Arabidopsis thaliana in NCBI:

AtAMT1.1 (NP_193087.1)

MSCSATDLAVLLGPNATAAANYICGQLGDVNNKFIDTAFAIDNTYLLFSAYLVFSMQLGF
AMLCAGSVRAKNTMNIMLTNVLDAAAGGLFYYLFGYAFAFGSPSNGFIGKHYFGLKDIP
TASADYSNFLYQWAFAIAAAGITSGSIAERTQFVAYLIYSSFLTGFVYPVVSHWFWSVDG
WASPFRTDGDLLFSTGAIDFAGSGVVHMVGGIAGLWGALIEGPRLGRFDNGGRAIALRG
HSASLVVLGTFLLWFGWYGFNPGSFNKILVTYETGTYNGQWSAVGRTAVTTTLAGCTAA
LTTLFGKRLLSGHWNVTDVCNGLLGGFAAITGGCSVVEPWAAIICGFVAALVLLGCNKL
AEKLKYDDPLEAAQLHGGCGAWGLIFTALFAQEKYLNQIYGNKPGRPHGLFMGGGGKL
LGAQLIQIIVITGWVSATMGTLFFILKKMKLLRISSEDEMAGMDMTRHGGFAYMYFDDDE
SHKAIQLRRVEPRSPSPSGANTTPTPV

AtAMT1.2 (AEE34288.1)

MDTATTTCSAVDLSALLSSSSNSTSSLAAATFLCSQISNISNKLSDTTYAVDNTYLLFSAYL
VFAMQLGFAMLCAGSVRAKNTMNIMLTNVLDAAAGAISYYLFGFAFAFGTPSNGFIGRH
HSFFALSSYPERPGSDFSFFLYQWAFAIAAAGITSGSIAERTQFVAYLIYSTFLTGFVYPTVS
HWFWSSDGWASASRSDNNLLFGSGAIDFAGSGVVHMVGGIAGLCGALVEGPRIGRFDRS
GRSVALRGHSASLVVLGTFLLWFGWYGFNPGSFLTILKGYDKSRPYYGQWSAVGRTAVT
TTLSGCTAALTTLFSKRLLAGHWNVIDVCNGLLGGFAAITSGCAVVEPWAAIVCGFVAS
WVLIGFNLLAKKLKYDDPLEAAQLHGGCGAWGLIFTGLFARKEYVNEIYSGDRPYGLFM
GGGGKLLAAQIVQIIVIVGWVTVTMGPLFYGLHKMNLLRISAEDEMAGMDMTRHGGFA
YAYNDEDDVSTKPWGHFAGRVEPTSRSSTPTPTLTV

AtAMT1.3 (AEE76886)

MSGAITCSAADLATLLGPNATAAADYICGQLGTVNNKFTDAAFAIDNTYLLFSAYLVFA
MQLGFAMLCAGSVRAKNTMNIMLTNVLDAAAGGLFYYLFGYAFAFGGSSEGFIGRHNF
ALRDFPTPTADYSFFLYQWAFAIAAAGITSGSIAERTQFVAYLIYSSFLTGFVYPVVSHWF
WSPDGWASPFRSADDRLFSTGAIDFAGSGVVHMVGGIAGLWGALIEGPRRGRFEKGGRA
IALRGHSASLVVLGTFLLWFGWYGFNPGSFTKILVPYNSGSNYGQWSGIGRTAVNTTLSG
CTAALTTLFGKRLLSGHWNVTDVCNGLLGGFAAITAGCSVVEPWAAIVCGFMASVVLIG
CNKLAELVQYDDPLEAAQLHGGCGAWGLIFVGLFAKEKYLNEVYGATPGRPYGLFMGG
GGKLLGAQLVQILVIVGWVSATMGTLFFILKRLNLLRISEQHEMQGMDMTRHGGFAYIY
HDNDDESHRVDPGSPFPRSATPPRV

AtAMT1.4 (At4g28700)

MASALSCSASDLIPLLSGGANATAAAAAAEYICGRFDTVAGKFTDAAYAIDNTYLLFSAY
LVFAMQLGFAMLCAGSVRAKNTMNIMLTNVIDAAAGGLFYYLFGFAFAFGSPSNGFIGK
HFFGMYDFPQPTFDYPYFLYQWTFAIAAAGITSGSIAERTQFVAYLIYSSFLTGLVYPIVSH
WFWSSDGWASPARSENLLFQSGVIDFAGSGVVHMVGGIAGLWGALIEGPRIGRFGVGGK
PVTLRGHSATLVVLGTFLLWFGWYGFNPGSFATIFKAYGETPGSSFYGQWSAVGRTAVT

TTLAGCTAALTTLFGKRLIDGYWNVTDVCNGLLGGFAAITSGCSVVEPWAALVCGFVAA
WVLMGCNRLAEKLQFDDPLEAAQLHGGCGAWGIIFTGLFAEKRYIAEIFGGDPNRPFGLL
MGGGGRLLAAHVVQILVITGWVSVTMGTLFFILHKLKLLRIPAEDEIAGVDPTSHGGLAY
MYTEDEIRNGIMVRRVGGDNDPNVGV

AtAMT1.5(AEE76885.1)
MSGAITCSAADLSALLGPNATAAADYICGQLGSVNNKFTDAAYAIDNTYLLFSAYLVFA
MQLGFAMLCAGSVRAKNTMNIMLTNVLDAAAGGLFYYLFGYAFAFGESSDGFIGRHNF
GLQNFPTLTSDYSFFLYQWAFAIAAAGITSGSIAERTKFVAYLIYSSFLTGFVYPVVSHWF
WSPDGWASPFRSEDRLFGTGAIDFAGSGVVHMVGGIAGLWGALIEGPRIGRFPDGGHAIA
LRGHSASLVVLGTFLLWFGWYGFNPGSFTKILIPYNSGSNYGQWSGIGRTAVTTTLSGCT
AALTTLFGKRLLSGHWNVTDVCNGLLGGFAAITAGCSVVDPWAAIVCGFVASLVLIGCN
KLAELLKYDDPLEAAQLHGGCGAWGLIFVGLFAKEKYINEVYGASPGRHYGLFMGGGG
KLLGAQLVQIIVIVGWVSATMGTLFFILKKLNLLRISEQHEMRGMDLAGHGGFAYIYHDN
DDDSIGVPGSPVPRAPNPPAV
AtAMT2.1 (NP_181363)
MAGAYDPSLPEVPEWLNKGDNAWQLTAATLVGLQSMPGLVILYASIVKKKWAVNSAF
MALYAFAAVLLCWVLLCYKMAFGEELLPFWGKGGPAFDQGYLKGQAKIPNSNVAAPYF
PMATLVYFQFTFAAITTILVAGSVLGRMNIKAWMAFVPLWLIFSYTVGAYSIWGGGFLYQ
WGVIDYSGGYVIHLSSGVAGFVAAYWVGPRPKADRERFPPNNVLLMLAGAGLLWMGW
SGFNGGAPYAANLTSSIAVLNTNLSAATSLLVWTTLDVIFFGKPSVIGAIQGMVTGLAGVT
PGAGLIQTWAAIIIGVVSGTAPWASMMIIHKKSALLQKVDDTLAVFYTHAVAGLLGGIMT
GLFAHPDLCVLVLPLPATRGAFYGGNGGKQLLKQLAGAAFIAVWNVVSTTIILLAIRVFIP
LRMAEEELGIGDDAAHGEEAYALWGDGEKFDATRHVQQFERDQEAAHPSYVHGARGV
TIVL

Lycopersicon esculentum in NCBI:
LeAMT1.1 (NP_001304667.1)
MACSVDTLAPFLGPNTTNAVAAASYICNQFSGVSDRFVDTGYAIDSTYLLFSAYLVFSMQ
LGFAMLCAGSVRAKNTMNIMLTNVLDAAAGGLFYYLFGFAFAWGGPSNGFIGRHFFGL
KEIPSNSFDYSNFLYQWAFAIAAAGITSGSIAERTQFVAYLIYSSFLTGFVYPVVSHWFWTP
DGWASPTNSNLLFGSGVIDFAGSGVVHMVGGIAGFYGALIEGPRIGRYDHTGRSVALRGH
SASLVVLGTFLLWFGWYGFNPGSFNKILVTYGASGGYYGQWSAVGRTAVTTTLAGCTA
ALTTLFGKRILSGHWNVTDVCNGLLGGFAAITAGCSVVEPWAAIICGFVAALVLIGFNML
AEKFKYDDPLEAAQLHGGCGAWGIIFTGLFAKGEFVDQVYPGKPGRPHGLFMGGGGKLL
GAHIIQILVIIGWVSATMGPLFYILHKFKLLRISSEDEMAGMDLTRHGGFAYYHEEDPKLG
MQMRRIEPTTST

LeAMT1.2 (NP_001234253.2)
MASAMTCSAAELFPHLGSSANATAAAEFICSRFSAVSEYLTNTTYAVDTTYLLFSAYLVF
AMQLGFAMLCAGSVRAKNTMNIMLTNVLDAAAGGLSYYLFGFAFAFGAPSNGFIGKHFF
GLKEFPSQAFDYSYFLYQWAFAIAAAGITSGSIAERTQFVAYLIYSSFLTGFVYPIVSHWF
WSGDGWASASKTDGNLLFGSGVIDFAGSGVVHMVGGIAGLWGAFIEGPRIGRFDRSGRS
VALRGHSASLVVLGTFLLWFGWYGFNPGSFLTILKSYDHTIRGTYYGQWSAIGRTAVTTT

LAGCTAALTTLFCKRLLVGHWNVVDVCNGLLGGFAAITSGCAVVEPWAAIVCGFIAAW
VLIGFNALAAKLKYDDPLEAAQLHGGCGSWGIIFTGLFAKKEYVNEVYPGFPNRPYGLF
MGGGGKLLGAQVIQVVVIIGWVSVTMGPLFYLLHKFKLLRISRDDETAGMDLTRHGGFA
YIYHDEDEGSSMPGFKMTRVEPTNTSTPDHQNRSVNVVV

LeAMT1.3 (NP_001234216.1)
MDSSWEASVTDSINAIYLLFSAYLVFVMQLGFAMLCAGSVRAKNAMNIMLTNVVDAVV
GSLSYYLFGFAFAFGDSNPFIGASYFALKDIPSSSYDYSFFLYQWAFAIAVAGITSGSIAERT
QFTAYLVFSFFLTGFVYPVVAHWLWSSNGWLSPNSTYLLFGSGAIDFAGSGVVHLVGGIA
GFWGSIVEGPRVGRFDAFGNPVKMRGHNATLVVLGTLLLWFGWFGFNPGSFNKILVAYP
HMADQGNWTSVGRTAVTTTLAGSTAGIVTLFGRRLLVGHWDAMDVCNGVLGGFVAITS
GCSVVEPWAAILCGFCAAWVLIGLNILALKFKFDDPLEAAQLHGGCGAWGLIFTGLFAKE
EFVLQAYNSGKTQIIRPSGLILGGGWGLFGAQIVELLSIVVWVSLTMGPLFYLLQKLGILRI
SSDEEVAGLDISSHGGYAYDASQEESNARFYGEYLRMQQQ

Lotus japonicas in GenBank:
LjAMT1.2 (AAM95453)
MAALPECSAANLAQLIGPNATDAAAVAGFICDQFTAVGQRFSDTAFAVDSTYLLFSAYLV
FSMQLGFAMLCAGSVRAKNTMNIMLTNVLDAAAGGLFYYLFGFAFAFGAPSNGFIGRHF
FGLKDVPTVAFDYSYFLYQWAFAIAAAGITSGSIAERTQFVAYLIYSSFLTGFVYPVVSHW
FWSGDGWASATNTGNLLFGTGVIDFAGSGVVHMVGGVAGLWGALIEGPRIGRFDHAGR
AVSLRGHSASLVVLGTFMLWFGWYGFNPGSFTKILSAYDSGNYYGQWSAIGRTAVTTTL
AGCTAALTTLFGKRIISGHWNVTDVCNGLLGGFAAITAGCSVVEPWAAIVCGFVAAVVLI
ACNMLAEKVRYDDPLEAAQLHGGCGAWGIIFTALFAKEEYVNQVYPGKPGRPYGLFMG
GGGKLLGAHVIQILVIIGWVSATMGPLFFILNKMKLLRISTEDELAGMDLTRHGGFAYAY
EDDESHKPGIQLRKIEPNSSSTPSAES

LjAMT1.1 (CAC10555.1)
MASLSCSATDLAPLLTATTNATATAAATYLCNQLDTISRKLSDTTYAVDNTYLLFSAYLV
FAMQLGFAMLCAGSVRAKNTMNIMLTNVLDAAAGGLSYYLFGFAFAFGAPSNGFIGRHF
FGLKHYPSPTYDYSFFLYQWAFAIAAAGITSGSIAERTQFVAYLIYSSFLTGFVYPVVSHW
LWSSDGWASPTRTTGSLLFGSGAIDFAGSGVVHMVGGIAGLWGAFIEGPRIGRFDRTGRS
VALRGHSASLVVLGSFLLWFGWYGFNPGSFLTIAKAYGNNGENGNYYGQWSAIGRTAV
TTTLAGCTAALTTLFSKRLLEGHWKVLDVCNGLLGGFAAITSGCSVVEPWPAIVCGFVAA
WVLIGLNLVAAKMKYDDPLEAAQLHGGCGAWGVLFTGLFAKGEYVAEVYGSAGRPYG
LLMGGGGKLLAAQVIEILVVCGWVTATMGPLFYGLHKTKLLRISEDDETAGMDLTRHGG
FAYAYHDDDDVSTKRGVMMSRIGPGSSSPSTMNTPAASAAANDC

LjAMT1.3 (CAE01484)
MAAAFTCSAADLHPLLGTGANATAAAEFICSRFGAISNKFTDTAYAVDNTYLLFSAYLVF
AMQLGFAMLCAGSVRAKNTMNIMLTNVLDAATGGIFFYIFGFALAFGTPSNGFIGKHFFG
LNEFPSPSFDYGFFLYQWAFAIAAAGITSGSIAERTQFVAYLIYSSFLTGLVYPIVAHWFWS
SDGWGSPARTENLLFGSGVIDFAGSGVVHLVGAVAGFWGAFIEGPRMGRFDHAGKPVPL
RGHSGTLVVLGTFLLWFGWYGFNPGSFITILKSYGESGSGNYYGQWSAIGRTAVTTTLAG

CSAALTTLFGKRLQTGHWNVTDVCNGLLGGFAAITSGCSVVDPWAAIICGFVAAWVLIG
CNVLAEKFHYDDPLEAAQLHGGCGTWGIIFTALFAKKQYVNEVYAGLPDRPYGLLMGG
GWRLLAAHVVQILTIAGWVSVTMGAVFFVLHKLNLLRISPEEEMAGLDMTSHGGLAYEY
HEDVGSVQKALKFVFDFYCKGNYYKTLLE

LjAMT2.1 (AAL08212.1)
MATPTAYQENLPASPEWLNKGDNAWQMTAATLVGLQSMPGLVILYASIVKKKWAVNS
AFMALYAFAAVLLCWVLLCYRMAFGDKLFPFWGKGAPALGQKFLINQASVPESTHYFN
NGSIETQTQRPFYPMATLVYFQFNFAAITMILLAGSVLGRMNIKAWMAFVPLWLIFSYTV
GAFSIWGGGFLYHWGVIDFSGGYVIHLSSGIAGFTAAYWVGPRLKSDRERFPPNNVLLML
AGAGLLWMGWSGFNGGAPYAANIDASIAVLNTNICAATSLLVWTSLDVAFFGKPSVIGA
VQGMMTGLVCITPGAGLVQSWAAIVMGILSGSIPWVSMMILHKKSSLLQKVDDTLGVFH
THAVAGLLGGLLTGLLAEPELCRLVLPVSNSRGAFYGGTGGAQFLKQLVAALFVIGWNL
VSTTLILLAIQLFIPLRMPDYQLEIGDDAVHGEEAYALWGDGEKYDPTKHGSTIVDETLAS
AYSVGARGVTVNL

Nicotiana tabacum in GenBank:
NtAMT1.1 (XP_009784484.1)
MACEVNQLAPFLGPNTTDAVAAATYICSQFSDVSNKFVDTGYAIDSTYLLFSAYLVFSMQ
LGFAMLCAGSVRAKNTMNIMLTNVLDAAAGGLFYYLFGFAFAWGGPSNGFIGRHFFGL
KEIPSSSFDYSNFLYQWAFAIAAAGITSGSIAERTQFVAYLIYSSFLTGFVYPVVSHWFWAP
DGWASPTNSNLLFGSGVIDFAGSGVVHMVGGIAGFYGALIEGPRIGRFDHAGRSVALRGH
SASLVVLGTFLLWFGWYGFNPGSFNKILVTYGTSGGYYGQWSAVGRTAVTTTLAGCTA
ALTTLFGKRILSGHWNVTDVCNGLLGGFAAITAGCSVVEPWAAIICGFIAALVLIGCNKLA
EIFKYDDPLEAAQLHGGCGAWGIIFTALFAKGSYVDQVYPGKPGRPHGLFMGGGGKLLG
AHIIQILVIFGWVTATMGPLFYILHKFKLLRISSEDEMAGMDLTRHGGFAYYHDEDLKHG
TQMRRIEPTSSS

NtAMT1.2 (XP_009795683.1)
MASAVTCSAADLLPHLGGSANATAAAEFICDRFTAVSEYLTNTTYAVDNTYLLFSAYLVF
AMQLGFAMLCAGSVRAKNTMNIMLTNVLDAAAGGLSYYLFGFAFAFGTPSNGFIGKHFF
GLKEFPSPSFDYSYFLYQWAFAIAAAGITSGSIAERTQFVAYLIYSSFLTGFVYPIVSHWFW
SGDGWASASKTDGNLLFGSGVIDFAGSGVVHMVGGIAGLWGAFIEGPRIGRFDRTGRSV
ALRGHSASLVVLGTFLLWFGWYGFNPGSFLTILKSYDSPKGVYYGQWSAIGRTAVTTTL
AGCTAALTTLFSKRLLVGHWNVIDVCNGLLGGFAAITSGCAVVEPWAAIVCGFVAAWVL
IGFNKLAVKLKYDDPLEAAQLHGGCGSWGIIFTGLFAKKAYVNEVYPGFPDRPYGLFMG
GGGKLLGAQIIQILVIIGWVSVTMGPLFYLLHKFKLLRICREDEEAGMDLTRHGGFAYLYH
DEDEGSSMPGFKMTRIEPTNTPTPDNHNSRSVVV

NtAMT1.3 (XP_009768027.1)
MDTSWEASVTDSINAIYLLFSAYLVFVMQLGFAMLCAGSVRAKNAMNIMLTNVVDAVV
GSISYYLFGFAFAFGDASNSNPFIGTNYFALKDIPSSSYDYSFFLYQWAFAIAVAGITSGSIA
ERTQFTAYLFFSFFLTGFVYPVVAHWLWSSNGWLSPNSDSLLFGSGAIDFAGSGVVHLVG
GIAGLWGSLIEGPRVGRFDAFGNPVKMRGHNATLVVLGTFLLWFGWFGFNPGSFDKILV

PYPNKIDQGNWTSVGRTAVTTTLAGSTAGIVTLFGRRILVGHWDATDVCNGVLGGFVAI
TSGCSVVEPWAAIVCGFWAAWVLIGLNILALKLKFDDPLEAAQLHGGCGAWGLIFTGLF
AKEEFVLQAYNSGKTQVARPSGLILGGGWGLFGAQVVELLSIVVWVSLTMGPLFYALQK
LGILRITADEEIAGLDISSHGGYAYEANQEENGPRFYGEYLRMQHQS

NtAMT2.1 (XP_009778221.1)
MSIPGAYQTDLPAVPPWLNKGDNAWQMTAATLVGLQSMPGLVILYASIVKKKWAVNSA
FMALYAFAAVLICWVLVGYRIAFGDKLLPFWAKGAPALGQKYLIGRARVPETTHYYSNG
NIESPMLEPFYPMAAHVYFHFTFAAITMILLAGSVLGRMNIKAWMAFVPLWLIFCYTVGA
FSLWGGGFLYHWGVIDYSGGYVIHLSAGISGFTAAYWVGPRLKSDRERFPPNNVLLMLA
GAGLLWMGWSGFNGGAPNAANVAAPLAVLNTNISAATSLLVWTTLDVFYFGKPSVIGA
VQGMMTGLACVTPGAGVVQAWAAIVMGILSGSIPWFSMMILHKKSTFLQKVDDTLAVF
HTHAVAGLLGGLLTGLLAEPSLCSIILPVSNTRGAFYGGSGGMLFLKQIVAALFIIGWNIV
ATTIILLAIRLFIPLRMSDEQLMIGDDAVHGEEAYALWGDGEKYDPTRHGWHGSDTPQETI
PTGFVNGARGVTINL

NtAMT3.1(XP_009800812.1)
MDTVVPQAYLNNTSTAPDWLNKGDNSWQLISATLVGLQSVPGLVILYGSIVKKKWAVN
SAFMALYAFAAVIICWVAWAYKMSFGEQLLPFWGKAGPALGQKFLIKQAALPATTHYFS
DHTTVETAMATPFYPMASMVWFQCVFAAIALILLAGSLLGRMNIRAWMMFVPLWLTFS
YTVGAFSLWGGGFLYHWGVIDYSGGYVIHLSSGIAGITAAYWVGPRTKSDRERFPPNNIL
LMLAGAGLLWMGWAGFNGGDPYSANIDSSIAVLNTNICAATSLLVWTWLDVIFFEKPSV
IGAVQGMITGLVCITPGAGLVQGWAAIIMGILSGSVPWFTMMIVHKRWPLLQKIDDTLGV
FHTHAVAGFLGGTLTGLFAEPVLCNLFLPVTNTRGGVYGGLGGVQFMKQIVGGGFIIGW
NVVVTSIICYLISFLMPLRMSEEQLKIGDDAVHGEEAYALWGDGEVYDPTKHGFSSPDKT
THGKLARGTTEMI

NtAMT4.1(XP_009799644.1)
MDPNNSYLPQGLKPDESTPQWLSKGDNAWELTAAMVGLQSVPGLVILYGSMVKKKW
AVNSAFMALYAFAAVLICWVLWAHRMAFGTHLISIIGKPEQAMTQDFLLTKHSKYYIPTA
DYVFYQFAFAAITVILLAGSLLGRMNFYAWMLFVPLWITFSYTVGAYSIWGSGFLEPIIDF
SGGYVIHLSSGVAGFTAAYWVGPRHSQDRQHFPPNNIIHMLGGAGFLWMGWTGFNGGS
PLASDLITSLAILNTHLCTATSLLVWLSMDMIFYNKSSVIGAVQGMITGLVCITPGAGIVES
WAAILMGISSGSIPWFTMMVLHKKSAFFQKVDDTLGVFHTHAVAGLLGGILSGFFAKPKL
LRMFYGSDKYGPGFLYSIIDGDIKRGVRQMIYQILGAAFITIWNVVATSLICIFISRIVELRL
HEDDLEIGDDAVHGEEAYALWGDGERDPPRFNRTPKIPSFCRRQYGTTQ

NtAMT4.2(XP_009784746.1)
MSLLAPPPPFPPAFNFSMLPSNLNPNDANPPWMSKGDNAWQLIAATLVGMQSVPGLIILY
GGAVKKKWAVNSAFMALYAFACVLVCWVCWGYRLSFGEKLIPIWGKVDVALEQEYLF
EQAFLGLFPNATMVFFQFVFAAITLILIAGALLGRMNFYAWMLFVPLWLTFSYTIGAYTI
WSTNGWLSLKGIIDYSGGYVIHLSSGVAGFTAAYWVGPRSTKDRERFPPNNILLMLAGAG
LLWMGWTGFNGGDPYAASIDASLAVLNTHVAAATSLLTWLILDLIFFGKPSVIGAVQGMI
TGLVAITPAAGVVQGWAAIIIGLCSGSIPWFTMMVVHKKSELLQKVDDTMAVFHTHAVA

GSLGGILAGLFANPRLCYLFYGYYNKYYGFFYGLRDGKIHNGFRQMGLQLLGILFIVVVN
VVMTSLICLLVQLIVPLRMSEEDMEIGDEAAHGEEAYAIWGQGDRLEKSAGFSAYNDDM
ITAGASRSNYNTSKSQVEMV

NtAMT4.3(XP_009796398.1)
MAYLPRNLQPDDANPPWMNKADNAWQLTSATLVGLQSVPGLVILYGSIVKKKWALNSA
LMALYAFAAVLVCWVGWGYQLAFGDTLVPFLGKPDIHALDQKFLMSQAFIGKYPNASM
VYFQFVFAAITLILIAGALLGRMNFIAWMLFVPLWLTFSYTVGAFSIWCPQGWLFRMGVI
DYSGGFVIHLSSGVAGFTAAYWVGPRAPRDRERFPPNNILLMLAGAGILWMGWTGFNGG
DPYVASLDASLAVLNTHVCAAASLLTWLMLDILFYEKPSVIGATQGMITGLVCITPAAGV
VQGWAAILMGLMSGCIPWFSMMFLHKKMWLLKQVDDTMAVFHTHAVAGTLGAILAGV
LANPRLSRIFYMVDDWPKYIGLAYGIQSGRSRAGVRQLWVQLIGIGFVFVWNVISTSVICL
LIRMVVPLRMTEEEVSEGDNAVHGEEAYALWGDGEKFDNSRLQLVHEIEGAPLSKSGYG
L

NtAMT4.4(XP_009765535.1)
MELPSNLSTDEASPEWMNKGDNAWQLTAATLVGLQCVPGLVILYGGMVKKKWAINSAF
MALYAFASVLICWVGWGYRMSFGDKLVAFWGKPAVAMDEKYLLGQAFLGYFPTASMV
FFQFVFAAITPILVAGALLGRMNFIAWMLFVPLWHTFSYTIGAFSIWCPEGWLSKLGVIDF
AGGYVIHLSSGVAGFTAAYWVGPRLDKDRERFPPNNILMMLAGAGLLWMGWTGFNGG
APYTASTDASLAVLNTHVCTATSLLTWLVLDMSVFGKPSVIGAVNGMITGLVCITPGAGV
VQCWAAILMGLTSGSVPWYTMTVLHKKIKFLRHVDDTFAVFHTHALAGILGGILTGFFA
VPKLCRLFYLVPEWERYIGLAYGLQTGRTSAGLRQMGAQLAGIGFIVCLNIVMTSLVCLFI
KLIVPLRLEEEVLKIGDDAIHGEEAYAIWDDEEKYENTQVNSAYDADEYPSVVSKTVSEF
QMV

Oryza sativa in GenBank:
OsAMT1.1 (CAE03364.1)
MATCAADLAPLLGPVAANATDYLCNRFADTTSAVDATYLLFSAYLVFAMQLGFAMLCA
GSVRAKNTMNIMLTNVLDAAAGALFYYLFGFAFAFGTPSNGFIGKQFFGLKHMPQTGFD
YDFFLFQWAFAIAAAGITSGSIAERTQFVAYLIYSAFLTGFVYPVVSHWIWSADGWASAS
RTSGPLLFGSGVIDFAGSGVVHMVGGVAGLWGALIEGPRIGRFDHAGRSVALKGHSASL
VVLGTFLLWFGWYGFNPGSFTTILKTYGPAGGINGQWSGVGRTAVTTTLAGSVAALTTL
FGKRLQTGHWNVVDVCNGLLGGFAAITAGCSVVDPWAAIICGFVSAWVLIGLNALAARL
KFDDPLEAAQLHGGCGAWGILFTALFARQKYVEEIYGAGRPYGLFMGGGGKLLAAHVIQ
ILVIFGWVSCTMGPLFYGLKKLGLLRISAEDETSGMDLTRHGGFAYVYHDEDEHDKSGV
GGFMLRSAQTRVEPAAAAASNSNNQV

OsAMT1.2 (BAD21532.1)
MATCLDSLGPLLGGAANSTDAANYICNRFTDTSSAVDATYLLFSAYLVFAMQLGFAMLC
AGSVRAKNSMNIMLTNVLDAAAGALFYYLFGFAFAFGTPSKGFIGKQFFGLKHMPQTGY
DYDFFLFQWAFAIAAAGITSGSIAERTRFSAYLIYSAFLTGFVYPVVSHWFWSTDGWASA
GRLTGPLLFKSGVIDFAGSGVVHLVGGIAGLWGAFIEGPRIGRFDAAGRTVAMKGHSASL
VVLGTFLLWFGWFGFNPGSFTTISKIYGESGTIDGQWSAVGRTAVTTSLAGSVAALTTLY

GKRWLTGHWNVTDVCNGLLGGFAAITAGCSVVDPWASVICGFVSAWVLIGCNKLSLILK
FDDPLEATQLHAGCGAWGIIFTALFARREYVELIYGVPGRPYGLFMGGGGRLLAAHIVQI
LVIVGWVSATMGTLFYVLHRFGLLRVSPATEMEGMDPTCHGGFGYVDEDEGERRVRAK
SAAETARVEPRKSPEQAAAGQFV

OsAMT1.3 (BAD21574.1)
MATCADTLGPLLGTAAANATDYLCNQFADTTSAVDSTYLLFSAYLVFAMQLGFAMLCA
GSVRAKNTMNIMLTNVLDAAAGALFYYLFGFAFAFGAPSNGFIGKHFFGLKQVPQVGFD
YSFFLFQWAFAIAAAGITSGSIAERTQFVAYLIYSAFLTGFVYPVVSHWIWSADGWASASR
TSGSLLFGSGVIDFAGSGVVHMVGGVAGLWGALIEGPRIGRFDHAGRSVALRGHSASLV
VLGSFLLWFGWYGFNPGSFLTILKSYGPPGSIHGQWSAVGRTAVTTTLAGSTAALTTLFG
KRLQTGHWNVIDVCNGLLGGFAAITAGCSVVDPWAAIICGFVSAWVLIGLNALAARLKF
DDPLEAAQLHGGCGAWGVIFTALFARKEYVDQIFGQPGRPYGLFMGGGGRLLGAHIVVI
LVIAAWVSFTMAPLFLVLNKLGLLRISAEDEMAGMDQTRHGGFAYAYHDDDASGKPDR
SVGGFMLKSAHGTQVAAEMGGHV

OsAMT2.1 (BAC65231.1)
MAAAGAYSASLPAVPDWLNKGDNAWQLTASTLVGIQSMPGLVVLYGSIVKKKWAVNS
AFMALYAYASSLLVWVLVGFRMAFGDQLLPFWGKAGVALTQSYLVGRATLPATAHGAI
PRTEPFYPEATLVLFQFEFAAITLVLLAGSVLGRMNIKAWMAFTPLWLLLSYTVGAFSLW
GGGFLYRWGVIDYSGGYVIHLSSGIAGFTAAYWVGPRLKSDRERFSPNNILLMIAGGGLL
WMGWAGFNGGAPYAANIAASVAVLNTNVCAATSLLMWTCLDVIFFRKPSVIGAVQGM
MTGLVCITPGAGLVQTWAAVVMGIFAGSVPWFTMMILHKKSALLMKVDDTLAVFHTHA
VAGLLGGILTGLLATPELFSLESTVPGLRGAFYGGGIKQIGKQLGGAAFVIAWNLVVTTAI
LLGIGLFIPLRMPDEQLMIGDDAAHGEEAYALWGDGEKFDATRHDLSRGGGGGDRDGPA
GERLSALGARGVTIQL

OsAMT2.2 (CAY33634.1)
MHLRMASPPQPGPYMPDLPAVPAWLNKGDTAWQLVAATFVGIQSMPGLVVIYGSIVKK
KWAVNSAFMALYAYASTLIVWVLVGFRMAFGDRLLPFWAKAGPALTQDFLVQRAVFPA
TAHYGSDGTLETPRTEPFYAEAALVLFEFEFAAITLVLLAGSLLGRMNIKAWMAFTPLWL
LFSYTVGAFSLWGGGFLYQWGVIDYSGGYVIHLSSGVAGFTAAYWVGPRLKSDRERFSP
NNILLMIAGGGLLWLGWAGFNGGAPYAPNVTATVAVLNTNVSAATSLLTWTCLDVIFFG
KPSVIGAVQGMMTGLVCITPGAGLVHTWSAMLMGMFAGSVPWFTMMILHKKSTFLMK
VDDTLAVFHTHAVAGILGGVLTGLLATPELCALDCPIPNMRGVFYGSGIGQLGKQLGGAL
FVTVWNLIVTSAILLCIGLFIPLRMSDDQLMIGDDAAHGEEAYALWGDGEKFDVTRPETT
RTGGAGGAGREDTMEQRLTNMGARGVTIQL

OsAMT2.3 (NP_915334.1)
MASPTRPGPYMPRPPAVPEWLNTGDNGWQLAAATFVGLQSMPGLVVLYGSIVKKKWA
VNSAFMALYAYASTLIVWVLVGFRMAFGDRLLPFWGKAGAALTEGFLVARASVPATAH
YGKDGALESPRTEPFYPEASMVLFQFELAAITLVLLAGSLLGRMNIKAWMAFTPLWLLFS
YTVCAFSLWGGGFLYQWGVIDYSGGYVIHLSSGIAGFTAAYWVGPRLKSDRERFSPNNIL
LMIAGGGLLWLGWAGFNGGAPYAPNITASIAVLNTNVSAAASLLTWTCLDVIFFGKPSVI

GAVQGMMTGLVCITPGAGLVHTWAAILMGICGGSLPWFSMMILHKRSALLQKVDDTLA
VFHTHAVAGLLGGFLTGLFALPDLTAVHTHIPGARGAFYGGGIAQVGKQIAGALFVVVW
NVVATTVILLGVGLVVPLRMPDEQLKIGDDAAHGEEAYALWGDGERFDVTRHEGARGG
AWGAAVVDEAMDHRLAGMGARGVTIQL

OsAMT3.1 (BAD33268.1)
MSGDAFNMSVAYQPSGMAVPEWLNKGDNAWQMISATLVGMQSVPGLVILYGSIVKKK
WAVNSAFMALYAFAAVWLCWVTWGYNMSFGHKLLPFWGKARPALGQSFLLAQAVLP
QTTQFYKGGGGADAVVETPWVNPLYPMATMVYFQCVFAAITLILLAGSLLGRMNIKAW
MLFVPLWLTFSYTVGAFSLWGGGFLFHWGVMDYSGGYVIHLSSGVAGFTAAYWVGPRS
TKDRERFPPNNVLLMLTGAGILWMGWAGFNGGDPYSANIDSSLAVLNTNICAATSLLVW
TCLDVIFFKKPSVIGAVQGMITGLVCITPGAGLVQGWAAIVMGILSGSIPWFTMMVVHKR
SRLLQQVDDTLGVFHTHAVAGFLGGATTGLFAEPVLCSLFLPVTNSRGAFYPGRGGGLQF
VRQVAGALFIICWNVVVTSLVCLAVRAVVPLRMPEEELAIGDDAVHGEEAYALWGDGE
KYDSTKHGWYSDNNDTHHNNNKAAPSGVTQNV

OsAMT3.2 (BAD33268.1)
MAAGAIPMAYQTTPSSPDWLNKGDNAWQMTSATLVGLQSMPGLVILYGSIVKKKWAIN
SAFMALYAFAAVWICWVVWAYNMSFGDRLLPFWGKARPALGQSFLVAQSELTATAIRY
HNGSAEAPMLKPLYPVATMVYFQCMFASITIIILAGSLLGRMNIKAWMAFVPLWITFSYT
VCAFSLWGGGFLFQWGVIDYSGGYVIHLSSGIAGLTAAYWVGPRSASDRERFPPNNILLV
LAGAGLLWLGWTGFNGGDPYSANIDSSMAVLNTHICASTSLLVWTILDVFFFGKPSVIGA
VQGMITGLVCITPGAGLVQGWAAIVMGILSGSIPWYTMMVLHKKWSFMQRIDDTLGVFH
THAVAGFLGGATTGLFAEPILCSLFLSIPDSKGAFYGGPGGSQFGKQIAGALFVTAWNIVIT
SIICVIISLILPLRIADQELLIGDDAVHGEEAYAIWAEGELNDMTHHNESTHSGVSVGVTQN
V

OsAMT3.3 (AAO41130.1)
MSSSATVVPLAYQGNTSASVADWLNKGDNAWQLVAATVVGLQSVPGLVVLYGGVVKK
KWAVNSAFMALYAFAAVWICWVTWAYNMSFGEKLLPIWGKARPALDQGLLVGRAALP
ATVHYRADGSVETAAVEPLYPMATVVYFQCVFAAITLILVAGSLLGRMSFLAWMIFVPL
WLTFSYTVGAFSLWGGGFLFHWGVIDYCGGYVIHVSAGIAGFTAAYWVGPRAQKDRER
FPPNNILFTLTGAGLLWMGWAGFNGGGPYAANSVASMAVLNTNICTAMSLIVWTCLDVI
FFKKPSVVGAVQGMITGLVCITPAAGVVQGWAALVMGVLAGSIPWYTMMILHKRSKILQ
RVDDTLGVFHTHGVAGLLGGLLTGLFAEPTLCNLFLPVADSRGAFYGGAGGAQFGKQIA
GGLFVVAWNVAVTSLICLAINLLVPLRMPDDKLEVGDDAVHGEEAYALWGDGEMYDVT
KHGSDAAVAPVVV

OsAMT4.1 (Q10CV4.1)
MAAEAAPEWVEKGDNAWPLAAATLVGLQSVPRLVILYGDCGAVGPRTEKDREAFPPNN
VLLTLAGAGLLLWMGWTGFNGGAPYAANVDASVTVVNTHLCTATSLLVWLLLDSFVFG
RLSVISAVQGMITGLVCVTPAARLVLHKRSRLLARVDDTLAVLHTHGVAGSLSGVLTGLL
LLAEPRFARLFFGDDPRYVGLAYAVRDGRAGSGLRQVGVQLAGIAFVVALNVAVTSAVC
LAVRVAVPQLAGGGDAIHGEDAYAVWGDGETYEQYSVHGGGSNHGGFPMTANPVASK

ADEMIWI

Populus trichocarpa in UniProt:
PtrAMT1.1 (B9HSW3)
MASMTCSAGDLAQLLGSNVTNSTGAADFICSQFNTAADNFSATQYAVDNTYLLFSAYLV
FSMQLGFAMLCAGSVRAKNTMNIMLTNVLDAAAGGLFYYLFGFAFAFGTPSNGFIGKHN
FGLKNFPSSSFDYSYFLYQWAFAIAAAGITSGSIAERTQFVAYLIYSSFLTGFVYPVVSHWF
WSGDGWASATRTDGDLLFGTGVIDFAGSGVVHMVGGIAGLWGALIEGPRIGRFDHSGKA
IALRGHSASLVVLGTFLLWFGWYGFNPGSFTKILSAYPAGGYYGQWSAIGRTAVTTTLAG
CTAALTTLFGKRILSGHWNVTDVCNGLLGGFAAITAGCSVVEPWAAIICGFVASLVLIGC
NKLAEILKFDDPLEAAQLHGGCGTWGVIFTALFATEKYVREVYPNKPNRPYGLFMGGGG
KLLGAHLIQILVIIGWVSATMGPLFFVLRKLKLLRISSEDEMAGMDMTRHGGFAYIYHDD
ESNKHGFQLKRVEPTSRTPNANV

PtrAMT1.2 (B9IPE2)
MACSASDLAPLLSTTVNSTEAATYLCSQFTSISSQLSDTSYAINNTYLLFSAYLVFAMQLG
FAMLCAGSVRAKNTMNIMLTNVLDAAAGGLSYYLFGYAFAFGSPGNGFIGRHLFGLSDF
PTIQADYSFFLYQWAFAIAAAGITSGSIAERTQFVAYLIYSSFLTGFVYPVVSHWLWSGDG
WANPAKTDNKLLFGSGAIDFAGSGVVHMVGGIAGLWGALIEGPRIGRFDQNGRSVALRG
HSASLVVLGSFLLWFGWYGFNPGSFLTILKSYGGNRVFYGQWSAVGRTAVTTTLAGSTA
ALTTLFGKRLLSGHWNVIDVCNGLLGGFAAITAGCSVVEPWAAIICGFVAAWVLIGCNKL
ADKLQYDDPLEAAQLHGGCGMWGLLFTGLFAKETYVNEVYSNKPGRPYGLFMGGGGK
LLAAQIIEILVIVGWVSATMGPLFYGLHKLKLLRISAEDEMAGMDLTRHGGFAYAYDEED
DVSGKPSFMMKKVEPAKNTSPNGNSPAINV

PtrAMT1.3 (B9HKW8)
MASMNCSAGDLAQLLGPNVTNSTGAAAFICSQFQIAAGNFNATQFAVDNTYLLFSAYLV
FSMQLGFAMLCAGSVRAKNTMNIMLTNVLDAAAGGLFYYLFGFAFAFGTPSNGFIGKHN
FGLKAFPSSSFDYSYFLYQWAFAIAAAGITSGSIAERTQFVAYMIYSSFLTGFVYPVVSHW
FWSVDGWASATREHGDLLFGSGVIDFAGSGVVHMVGGIAGLWGALIEGPRIGRYHHSGR
SIALRGHSASLVVLGTFLLWFGWYGFNPGSFNKILSAYTDAPVYYGQWSAIGRTAVTTTL
AGCTAALTTLFCKRILSGHWNVTDVCNGLLGGFAAITAGCSVVEPWAAIVCGFVASLVLI
GCNKLAEIFKFDDPLEAAQLHGGCGTWGVIFTALFATEKYVGEVYPKKPGRPYGLFMGG
GGNLLAAHLIQVLVIIGWVSATMGPLFFVLHKLKLLRISAEDEMAGMDLTRHGGFAYIYH
DDDDESQRPGTFRLGQIEPTNSTTPSANA

PtrAMT1.4 (B9GRB5)
MAALTCSASDLQSLLGGAANATAAAEYICTRFVAVSDHFVDTAYAVDNTYLLFSAYLVF
AMQLGFAMLCAGSVRAKNTMNIMLTNVLDAATGGLFYYLFGFAFAFGSPSNGFIGKQFF
GLESFPSPSFDYGYFLYQWAFAIAAAGITSGSIAERTQFVSY
LIYSSFLTGLVYPIVSHWFWSADGWASAGRTDGNLLFGSGVIDFAGSGVVHMVGGIAGL
WGALIEGPRMGRFDHEGKSMALRGHSGTLVVLGTFLLWFGWYGFNPGSFLNILRTYGDV
GSYYGQWSAIGRTAVTTTLAGSSAALTTLFGKRMLAGNWNVTDVCNGLLGGFAAITSGC
AVVDPWAAIICGFVAAWVLIGCNKLADKFHYDDPLEAAQLHGGCGAWGIIFTALFAKET

YVNEIYSGKPGRPYGLLMGGGGRLLAAHMVQILVITGWVSVTMGTLFWILHKFKLLRIS
ADEEMAGMDLTSHGGLAYAYYDEHDDAMQKKSFMMTKADP

PtrAMT1.5 (B9GRB4)
MASSPLSCSASDLYPLLGDGANATAAAEFFCGRFEAISNKFVDTGYAVDSTYLLFSAYLV
FAMQLGFAMLCAGSVREKNTMNIMLTNVLDAAAGGLFYYTFGFALAFGSPSNGFIGQHF
FGLSKFPSPSFDYGYFLYQWAFAIAVAGITSGSIAERTQFV
AYLVYSSFLTGLVYPIVSHWFWSADGWASPARAENLLFGSGVIDFAGSGVVHLVGAVAG
LWGALIEGPRIGRFDHAGRAVTLRGHSGTLVVLGTFLLWFGWYGFNPGSFINISKSYESGS
YYGQWSAIGRTAATTTLAGCTAALTTLFGKRLLAGHWNVTDVCNGLLGGFAAITGGCSV
VDPWAAVLCGFVSAWVLIGCNMLAEKFHYDDPLEATQLHGGCGSWGIIFTALFAKEAY
VNEVYPGQPGRPYGLFMGGGARLLAAHIVQILVIVAWVSVTMGTVFFILHKLKLLRISAE
EEMAGMDLTSHGGLAYVYTDHEDEVKKQLGVV

PtrAMT1.6 (B9HP47)
MEVSWEQSVTDSINTIYLLFSAYLVFVMQLGFAMLCAGSVRAKNAMNIMLTNVVDAVV
GSISYYLFGFAFAFGDGTNSNPFIGTTFFALKDIPNTSYDYSYFLYQWAFAIAVAGITSGSIA
ERTQFSAYLVFSFFLSGFVYPIVVHWVWSSNGWLSPSSD
MLFGSGAIDFAGSGVVHLVGGIAGLWGSFIQGPRVGRFDAFGKPVPMRGHNATLVVLGT
FLLWFGWFGFNPGSFGKILVAYPNTTYQGNWTGIGRTAVTTTLAGSTAGLTTLFGRRLLV
GHWDALDACNGLLGGFVAITSGCSVVEPWAAIVCGFCAAWVLIGLNVLALKLQFDDPLE
ATQLHGGCGAWGLIFTGLFAKKEFVIQVYNSGEAGVVRPYGLLLGGGWGLIGCQVVELL
AIVAWVSITMGPVFFALDKLKMLRISIDEEVAGLDISSHGGYAYTTHPEENHPRFYADYM
PIQGRNHS

PtrAMT2.1 (B9HCZ0)
MNASTAYEQVSPAVPSWLNKGDNAWQMTASTLVALQSMPGLVILYASIVKKKWAVNS
AFMALYAFAAVLICWVLLGYRMAFGDELLPFWGKGAPALGQKYLLTRARVPESTHTLE
DGTRETVEPWYPMASLVYFQFTFAAITLILLAGSVLGRMNIKAWMAFVPLWLIFSYTVGA
FSLWGGGFLYHWGVIDYSGGYVIHLSSGIAGLTAAYWVGPRLKSDRERFPPNNVLLMLA
GAGLLWMGWSGFNGGAPYAANIDASIAVLNTNVCAATSLLVWTSLDVVYFGKPSVIGA
VQGMMTGLVCITPGAGLVQSWAAIVMGILSGSIPWVSMMILHKKSALLQKVDDTLGVFH
THAVAGLLGGLLTGLLAEPELCDLILPVNTRGAFYGGSGGVQFLKQVVAALFVIGWNVV
STTIILLFIRLFIPLRMPEEQLAIGDDAVHGEEAYALWGDGEKYDPTRHGRNTLYGEETAQ
SPYVNGARGVTINL

PtrAMT2.2 (B9IGE2)
MDAPAYEQVSPAVPSWLNKGDNAWQMIASILVATQSMPGLVILYASIVKKKWAVNSAF
MALYAFAAVLICWVLLCYRMAFGDELLPFWGKGAPALGQKYLIAQARIPESTHTHEDGT
RETVAPLYPMATLVYFQFTFAAITLILLAGSVLGRMNIKAWMAFVPLWLIFSYTVGAFSL
WGGGFLYHWGVIDYSGGYVIHLSSGIAGLTAAYWVGPRLKSDRERFPPNNVLLMLAGA
GLLWMGWSGFNGGAPYAANIDASMAMLNTNVCAATSLLVWTSLDVVYFGKPSVIGAV
QGMMTGLVCITPGAGLVQSWAAIVMGILSGSIPWVSMMILHKKFALLQKVDDTLGVFHT
HAVAGLLGGLLTGLLAEPELCDLILQVNTRGAFYGGNGGVQFLKQMVAALFVIGWNIVS

TTLILLFIRLFIPLRMPEEQLAIGDDAVHGEEAYALWGDGEKYDPSKHGRITTLFGEETTQS
PDVNGARGVTINL

PtrAMT3.1 (B9GHA5)
MAALPPNPVPVAYQGGSASVPDWLNKGDNSWQMISATLVGLQSMPGLVILYGSIVKKK
WAVNSAFMALYAFAAVVLCWVVWAYKMSFGDKLLPFWGKAGPALGQKFLIRQAELPA
TTHFYDNGEVETAMIQPFYPMASMVWFQCVFAAITLILLAGSVLGRMSFKAWMAFVPL
WLTFSYTVGAFSLWGGGFLFHWGVMDYSGGYVIHLSSGIAGLTTAFWVGPRSTKDRERF
PPNNVLLMLAGAGLLWMGWAGFNGGDPYTANIDSSMAVLNTNICAATSLLVWTWLDVI
FFKKPSVIGAVQGMITGLVCITPGAGLVQGWAAIIMGILSGSVPWFTMMIVHKRWTLIQKI
DDTLGVFHTHAVAGLLGGVLTGLFAEPQLCALFLPVTNSRGGVYGGSGGIQVLKQLVGA
AFIIGWNVVVTSIICVVINLVIPLRMSDEELLIGDDAVHGEEAYALWGDGEKYDSTKHGDT
TEEFPMERKSSTQVL

PtrAMT4.1 (B9GS88)
MNKGDNAWQLTAATLVGLQSVPGLVILYGSIVKKKWAVNSAFMALYAFASVLVCWVG
WGYQMSFGDKMIPFLGRPDVSLDQKFLLHQAFLGYLPNATMVYFQFVFAAITLILVAGA
LLGRMNFHAWMLFVPLWLTFSYTITAYSIWCPEGWLAKRGIIDYSGGYVIHLSSGVAGFT
AAYWVGPRTIKDRERFPPNNIILMLAGAGLLWMGWSGFNGGDPYTVSTDASLAVLNTH
VCTATSLLTWLLLDILFFGKPSVIGATQGMITGLVCITPAAGVVQGWAAILMGIMSGSIPW
YTMMVLHKKLWLLKQVDDTMAVFHTHAVAGSLGGILTGFFAHPKLNRIFYLVPDWQHY
IGLAYGLQMGRASAGFKQMGIQLLGILFVVSLNVFVTSIICLLIRFVVPLRLSDEELQTGDD
AIHGEEAYALWGDGEKYESRHNSVYAQNTTLIM

PtrAMT4.2 (B9IKS2)
MSNDTAFPPNLLPDEASPEWFNKADNAWQLTAATLVGLQSIPGLMILYGGGVKKKWAV
NSAFMVLYAFACVMFCWVTWGYRMSFGSKLLPFWGEANVALDQKYLLDPAFLGKFPN
ATMVYFQSVFAAITLILIAGAVLGRMNFYAWMIFVPLWLTFSYTFTAFSVWCPDGFLAK
MGLIDYSGGYVIHLSSGVAGYTAAYWVGPRLTKDRERFPPNNILLMLFGAGLLWMGWT
GFNGGDPYVVSTDASLAVLNTHLCTATSLLTWVALDIIFFRKASVIGAVQGMITGLVCITP
AAGVVQGWAATIMGLCSGSIPWITMMVIHKNSELLQKVDDTMAVLHTHAIAGSLGGILT
GLFAEPKLNRLFFGSSGHYIGLVYGFDDKSRIGSGVRQMGVQFIGILFVVFVNVLTTSIICIL
IRLVVPLRMSNEDLEIGDDAAHGEEAYAIWGNGDRQENSFYR

PtrAMT4.3 (B9H8E7)
MAEIPTNLLPDEASPEWMNKGDNAWQLTAATLVGLQSVPGLVILYGSIVKKKWAVNSAF
MALYAFAAVLVCWVGWGYHMSFGDKMLPFLGRPDISLDQKFLLDKAYVGYLPSATLV
YFQFVFAAITLILVAGALLGRMNFHAWMLFVPLWLTFSYTFTAYSLWCPGGWLAKLGIID
YAGGYVIHLSSGVAGFTAAYWVGPRTNKDRERFPPNNILLMLAGAGLLWMGWSGFNGG
GPFAANTDASLAILNTHVCTATSLLTWLLLDIVFFGKPSVIGATQGMITGLVCITPAAGVV
QGWAAILMGILSGSIPWYSMMVLHKKIWLLKQVDDTMAVFHTHAVAGSLGGILAGFFA
NPKLNRIFYMVKDWQHYIGLAYGLQNGRTAAGLKQMGVQLLGILFVVVLNVFVTSAICL
LIRLVVPLRLTDEELQTGDDAIHGEEAYALWGDGEKYESKHNSLHGVEEFPQVVSKEVE
MA

PtrAMT4.4 (B9I5F0)
MNKGDNAWQLTAATLVGLQSIPGLVILYGSIVKKKWAINSAFMVFYAFAMVLVCWVG
WGFRMSFGEKLVFFLGKPAVALDEKFLLGKAFLGYFPTATMVFFQGVFACITLILIAGCLL
GRMNFRAWIMFVPLWLTFSYTITAFSIWCPDGWLAKLGVIDFSGGYVIHLSAGVAGFTAA
YWVGPRIDKDREMFPPNNIILMLAGAGLLWMGWSGFNGGGPFAVSTISSLAVLNTHVCT
ATSLLTWLLLDTCFFGKPSVIGAVQGMITGLVCITPAAGVVQCWAAILMGIVSGSVPWYT
MMVLHKKVKFLRLVDDPIAIFHTHAIAGGLGGILTGFFAVPKLCRLFYMVPDWEKYIGLG
YGLQNGQTSAGLRQMGIQLGGILFVIFINISTTSMICWFVGLFVPLRLSDDELQIGDDAIHG
EEAFALWNDEETFQNTKTNSAFESEDSSYMKSRSFGDVQMV

Sorghum bicolor in NCBI:
SbAMT1.1 (XP_002446777)
MSTCAADLAPLLGPAAANATDYLCGQFADTASAVDATYLLFSAYLVFAMQLGFAMLCA
GSVRAKNTMNIMLTNVLDAAAGALFYYLFGFAFAFGTPSNGFIGKQFFGLQHLPKTGFD
YDFFLYQWAFAIAAAGITSGSIAERTQFVAYLIYSAFLTGFVYPVVSHWFWSADGWAAAS
RTSGPLLFGSGVIDFAGSGVVHMVGGIAGLWGALIEGPRIGRFDHAGRSVALKGHSASLV
VLGTFLLWFGWYGFNPGSFTTILKSYGPAGTVNGQWSAVGRTAVTTTLAGSVAALTTLF
GKRLQTGHWNVVDVCNGLLGGFAAITAGCSVVEPWAAVVCGFVSAWVLIGANALAAR
LKFDDPLEAAQLHGGCGAWGVLFTGLFASQKYVEEIYGSGRPYGLFMGGGGKLLAAQII
QILVIAGWVSCTMGPLFYALKKLDLLRISADDEMSGMDLTRHGGFAYVYHDEDPGDKSG
VGGFMLKSAQHRVEPAAATATSNQRSLEVARTSIRLTYQSIDEGSCWVGRAHQLPSAAA
GYR

SbAMT1;2 (XP_002452468.1)
MATCATTLAPLLGPAAANATEYLCNQFADTTSAVDSTYLLFSAYLVFAMQLGFAMLCA
GSVRAKNTMNIMLTNVLDAAAGALFYYLFGFAFAYGTPSNGFIGKHFFGLKRLPQVGFD
YDFFLFQWAFAIAAAGITSGSIAERTQFVAYLIYSAFLTGFVYPVVSHWVWSADGWASPT
RTSGNLLFGSGIIDFAGSSVVHMVGGIAGLWGALIEGPRIGRFDHAGRSVALRGHSASLVV
LGTFLLWFGWFGFNPGSFLTILKSYGPAGSIHGQWSAVGRTAVTTTLAGSTAALTTLFGK
RLQTGHWNVVDVCNGLLGGFAAITAGCSVVDPWAAVICGFVSAWVLIGLNALAARLRF
DDPLEAAQLHGGCGAWGVLFTALFARREYVEQIYGTPGRPYGLFMGGGGRLLAANVVM
ILVIAAWVSVTMAPLFLALNKLGLLRVSAEDEMAGMDQTRHGGFAYAYHDEDMSLSSR
PKGAQSTQIAAASSGEF

SbAMT2.1 (XP_002439939.1)
MAASGGAYAAQLPAVPEWLNKGDNAWQLTAATLVGIQSMPGLVVLYGSIVKKKWAVN
SAFMALYAYASSLLVWVLVGFRMAFGERLLPFWGKAGVALSQSYLIGRASLSATAHEGT
PRTEPLYPEATLVLFQFEFAAITLVLLAGSVLGRMNIKAWMAFTPLWLIFSYTVGAFSLW
GGGFLYHWGVIDYSGGYVIHLSSGVAGFTAAYWVGPRLKSDRERFSPNNILLMIAGGGL
LWMGWAGFNGGAPYAANIAASVAVLNTNVSAATSLLTWTCLDVIFFSKPSVIGAVQGM
MTGLVCITPGAGLVQTWAAVIMGVFAGSVPWFTMMILHKKSALLMKVDDTLAVFHTHA
VAGLLGGVLTGLLATPELLEIESPVPGLRGAFYGGGARQLGKQLAGAAFVVAWNVVVTS
LILLAIGLVVPLRMPDDQLMIGDDAAHGEEAYALWGDGEKFDATRHHDAARGGGGGG

MMDRDGSADQRMSGMGARGVTIQL

SbAMT2.2 (XP_002458715.1)
MASPPEPGPYMPDLPAVPAWLNKGDNAWQLVAATFVGLQSMPGLVVLYGSIVKKKWA
VNSAFMALYAYASTLIVWVLVGFRMAFGERMLPFWAKAGPALTQDFLVRRAVFPATAH
YGRGGALETPRTEPYYAQASLVLFEFEFAAITLVLLAGSLLGRMNIRAWMAFTPLWLLFS
YTVGAFSLWGGGFLYQWGVIDYSGGYVIHLSSGIAGFTAAYWVGPRLKSDRERFSPNNIL
LMIAGGGLLWLGWAGFNGGAPYAPNITASVAVLNTNVSAATSLLTWTCLDVIFFGKPSVI
GAVQGMMTGLVCITPGAGLVHTWSAMLMGAFAGSVPWFTMMILHKKSSLLMKVDDTL
AVFHTHAVAGVLGGVLTGLLATPELCALDSPVPGARGVFYGGGIAQMGKQLGGALFVT
VWNLVVTSAILLCIGLFIPLRMPDDQLMIGDDAAHGEEAYALWGDGEKFDVTRPDATRT
GGASGAAVTEDTVEQRLTSMGARGVTIQL

SbAMT3.1 (XP_002456706.1)
MASLPFNVTMTLGYQPGTGIPPWLNKGDNAWQMVAATLVGLQSVPGLVILYGSIVKKK
WAVNSAFMALYAFAAVWLCWVIWGYQMSFGQRLVPFWGKAGHTLGQGFLLSQAGLP
ATQHYYHDSDVVETFEITPFYPMASMVYFQCVFAAITLILLAGSLLGRMNFKAWMLFVPL
WLTFSYTIGAFSIWGGGFLFHWGVMDYSGGYVIHLSSGVAGFTAAYWVGPRLTKDRERF
PPNNVLLMLTGAGILWMGWAGFNGGDPYAANVDSSIAVLNTNICAATSLLVWTCLDVIF
FKKPSVIGAVQGMITGLVCITPGAGLVQGWAAILMGMISGSIPWFTMMVVHKRSRLLQQ
VDDTLGVFHTHAVAGFLGGFTTGLFAHPDLCPMFLPVTNSRGAFYGNGMQLVKQVVGA
LFIISWNVVVTSLVCLVVRLVVPLRMPDDELAIGDDAVHGEEAYALWGDGEKYDSTKHG
WYSDNDTQHNKAPSGVTQNV

SbAMT3.2 (XP_002466132.1)
MASSGTMPLAYQTTAASPDWLNKGDNAWQLTAATLVGLQSFPGLVVLYGGVVKKKWA
VNSAFMALYAFAAVWICWVTWAYNMSFGDKLLPLWGKARPALNQGYLIGQADLPATV
HYFANGSTIETAAAEPLYPMATVVYFQCVFAAITLILVAGSLLGRMSFAAWMLFVPLWLT
FSYTVGAFSVWGGGFLFQWGVIDYCGGYVIHLSAGFAGFTAAYWVGPRAQKDRERFPP
NNILFTLTGAGLLWMGWAGFNGGGPYAANVVASMSVLNTNICTAMSLIVWTCLDVIFFK
KPSVVGAVQGMITGLVCITPAAGVVQGWAAIVMGVLAGSVPWYTMMILHKRSRLLKHV
DDTLGVIHTHGVAGLLGGILTGLLADPTLCALFLPVTNSRGAFYGRAAGGAQLGKQLAG
ALFIIGWNVVVTSIICVAINAVVPLRMTEDKLEVGDDAVHGEEAYALWGDGEVYDVTEH
GPRGAAAVAPVSTTPN

SbAMT3.3 (XP_002452249.1)
MAAGAIPLAYQSSPSSPEWLNKGDNAWQMTSATLVGLQSMPGLVILYGSIVKKKWAINS
AFMALYAFAAVWICWVVWAYNMSFGDRLLPFWGKARPALGQSFLVAQSQLTATAVQY
RRRDDGTTAEAAMLRPLYPAATMVYFQCMFASITVIILAGSLLARMNIKAWMAFVPLWI
TFSYTVSAFSLWGGGFLFQWGVIDYSGGYVIHLSSGIAGLTAAYWVGPRSASDRERFPPN
NILLVLAGAGLLWLGWTGFNGGDPYAANIDSSMAVLNTHICASTSLLMWTLLDVFFFGK
PSVIGAVQGMITGLVCITPGAGLVQGWAAIVMGILSGSIPWYTMMVLHKKWSFMQRIDD
TLGVFHTHAVAGLLGGTTTGLFAEPVLCNLFLSIQESRGAFYGGDGGSQFGKQIAGALFVI
AWNIVITSIICVLIGLVLPLRISDEQLLIGDDAVHGEEAYAIWAEAELNDVTRHDEGRHSSV

AVGVTQNV

SbAMT4 (XP_002466457.1)
MATEAAPEWLDKGDNAWQLAAATLVGLQSVPGLVILYGSIVKKKWAVNSAFMALYAF
AATFVCWCLWAFRMSFGDRLLPFVGRPDLAALDQSFLTEQGFAGAYPAATLLFFQFVFA
AITLILVAGSLLGRINFRAWMLFVPLWLTFSYTIGAFSLWSPNGFLFKAGVMDFAGGYVIH
LSSGIAGFTAAYWKKLISIDRQVGPRTAKDREAFPPNNILLTLAGAGLLWMGWTGFNGG
APYAANIDASLAVVNTHFCTATSLLVWLCLDCFVFGRPSVIGAVQGMITGLVCITPAAGL
VQGWAAMLMGVVSGSVPWCTMMVLHKRCQFLKRVDDTLAVLHTHGVAGSLGGILTG
VLAEPRLSRLFFGDDPRYVGLVYALKDGRAAAGLRQVGMQLAGIAFVVALNVAVTSAV
CLVVGLLVPLRLSEEQLAAGDDAIHGEDAYAVWGDGQMYEQSVHGDHLYPMMSNPMA
SKADEMI

Triticum aestivum in GenBank:
TaAMT1.1 (AAS19466)
MSTCAASLAPLLGTAAANATDYLCNQFADTTTAIDSTYLLFSAYLVFAMQLGFAMLCAG
SVRAKNTMNIMLTNVLDAAAGALFYYLFGFAFAFGTPSNGFIGKHFFGLRDVPQVGFDY
SFFLFQWAFAIAAAGITSGSIAERTQFVAYLIYSAFLTGFVY
PVVSHWIWSADGWASASRTSGPLLFNSGVIDFAGSGVVHMVGGVAGLWGALIEGPRIGR
FDHAGRAVALRGHSASLVVLGTFLLWFGWYGFNPGSFLTILKSYGPPGSIHGQWSAVGR
AAVTTTLAGSTAALTTLFGKRLQTGHWNVLDVCNGLLGGFAAVTAGCSVVDPWAAIICG
FVSAWVLIGLNKLAARFKFDDPLEAAQLHGGCGAWGVIFTALFARREYVEQIYGAPGRP
YGLFMGGGGRLLGAHVVLILVIAAWVSCTMGPLFLALNKLGLLRISAEDEMAGMDQTR
HGGFAYAYTDEDSSSRPGRGAGGSVGGFMLKSAQTSQVAADATSPSSSV

TaAMT2.1 (AAR87397)
MSVPVAYQGNTSAAVADWLNKGDNAWQLTASTLVGLMSVPGMVVLYGGVVKKKWA
VNSAFMALYAFAAVWICWVVWAYNMSFGEELLPFWGKAGPALDQAFLVGRASLPATA
HYRADGTLETAMVEPYFPMATVVYFQCVFAAITLILVAGSLLGRMSFLAWMLFVPLWLT
FSYTVGAFSVWGGGFLFHWGVIDYCGGYVIHIPAGVAGFTAAYWVGPRTKKDRESFPPN
NILFALTGAGLLWMGWAGFNGGGPYAANVDSSMAILNTNICTAASLIVWTCLDAVFFKK
PSVVGAVQAVITGLVCITPGAGVVQGWAALVMGVLAGSVPWYTMMVLHKRSKLLQRV
DDTLGVIHTHGVAGLLGGVLTGLFAEPNLCNLFLPVTNSRGAFYGGNGGAQLGKQIAGA
LFVIGWNVVVVTSIICVVIRLVVPLRMSEEKLAIGDDAVHGEEAYALWGDGEHYDDTKHG
AAVVPV

Saccharomyces cerevisiae MEPs in Genbank:
ScMEP 1 (CAA97132.1)
MESRTTGPLTTETYDGPTVAFMILGAALVFFMVPGLGFLYSGLARRKSALALIWVVLMA
TLVGILQWYFWGYSLAFSKSAPNNKFIGNLDSFGFRNVYGKKFDEDAYPELAYATFQMM
FSCVNLSIIAGATAERGRLLPHMVFLFILATIGYCPVTYWIWSPGGWAYQWGVLDWAGG
GNIEILSAVSGFVYSWFLGKRNEKLLINFRPHNVSLVTLGTSILWFGWLLFNSASSLSPNLR
SVYAFMNTCLSAITGGMTWCLLDYRSEKKWSTVGLCSGIISGLVAATPSSGCITLYGSLIQ
GIVAGVVCNFATKLKYYAKVDDAMDILAEHGVAGVIGLIFNALFGADWVIGMDGTTEHE

GGWVTHNYKQMYKQIAYIAASIGYTAAVTAIICFVLGYIPGMRLRISEEAEEAGMDEDQI
GEFAYDYVEVRRDYYLWGVDEDSQRSDVNHRVNNAHLAAERSSSGTNSSSDGNGEMIQ
SEKILPIHQEDPANR

ScMEP2 (CAA96025.1)
MSYNFTGTPTGEGTGGNSLTTDLNTQFDLANMGWIGVASAGVWIMVPGIGLLYSGLSRK
KHALSLLWASMMASAVCIFQWFFWGYSLAFSHNTRGNGFIGTLEFFGFRNVLGAPSSVSS
LPDILFAVYQGMFAAVTGALMLGGACERARLFPMMVFLFLWMTIVYCPIACWVWNAEG
WLVKLGSLDYAGGLCVHLTSGHGGLVYALILGKRNDPVTRKGMPKYKPHSVTSVVLGT
VFLWFGWMFFNGGSAGNATIRAWYSIMSTNLAAACGGLTWMVIDYFRCGRKWTTVGL
CSGIIAGLVGITPAAGFVPIWSAVVIGVVTGAGCNLAVDLKSLLRIDDGLDCYSIHGVGGC
IGSVLTGIFAADYVNATAGSYISPIDGGWINHHYKQVGYQLAGICAALAWTVTVTSILLLT
MNAIPFLKLRLSADEEELGTDAAQIGEFTYEESTAYIPEPIRSKTSAQMPPPHENIDDKIVG
NTDAEKNSTPSDASSTKNTDHIV

NRTs:
Arabidopsis thaliana in NCBI:
AtNRT1.1 (NP_563899.1)
MSLPETKSDDILLDAWDFQGRPADRSKTGGWASAAMILCIEAVERLTTLGIGVNLVTYLT
GTMHLGNATAANTVTNFLGTSFMLCLLGGFIADTFLGRYLTIAIFAAIQATGVSILTLSTIIP
GLRPPRCNPTTSSHCEQASGIQLTVLYLALYLTALGTGGVKASVSGFGSDQFDETEPKERS
KMTYFFNRFFFCINVGSLLAVTVLVYVQDDVGRKWGYGICAFAIVLALSVFLAGTNRYR
FKKLIGSPMTQVAAVIVAAWRNRKLELPADPSYLYDVDDIIAAEGSMKGKQKLPHTEQF
RSLDKAAIRDQEAGVTSNVFNKWTLSTLTDVEEVKQIVRMLPIWATCILFWTVHAQLTTL
SVAQSETLDRSIGSFEIPPASMAVFYVGGLLLTTAVYDRVAIRLCKKLFNYPHGLRPLQRI
GLGLFFGSMAMAVAALVELKRLRTAHAHGPTVKTLPLGFYLLIPQYLIVGIGEALIYTGQ
LDFFLRECPKGMKGMSTGLLLSTLALGFFFSSVLVTIVEKFTGKAHPWIADDLNKGRLYN
FYWLVAVLVALNFLIFLVFSKWYVYKEKRLAEVGIELDDEPSIPMGH

AtNRT1.2 (NP_564978.1)
MEVEEEVSRWEGYADWRNRAAVKGRHGGMLAASFVLVVEILENLAYLANASNLVLYL
REYMHMSPSKSANDVTNFMGTAFLLALLGGFLSDAFFSTFQIFLISASIEFLGLIILTIQART
PSLMPPSCDSPTCEEVSGSKAAMLFVGLYLVALGVGGIKGSLASHGAEQFDESTPKGRKQ
RSTFFNYFVFCLACGALVAVTFVVWLEDNKGWEWGFGVSTIAIFVSILIFLSGSRFYRNKI
PCGSPLTTILKVLLAASVKCCSSGSSSNAVASMSVSPSNHCVSKGKKEVESQGELEKPRQE
EALPPRAQLTNSLKVLNG
AADEKPVHRLLECTVQQVEDVKIVLKMLPIFACTIMLNCCLAQLSTFSVQQAASMNTKIG
SLKIPPASLPIFPVVFIMILAPIYDHLIIPFARKATKTETGVTHLQRIGVGLVLSILAMAVAAL
VEIKRKGVAKDSGLLDSKETLPVTFLWIALQYLFLGSADLFTLAGLLEYFFTEAPSSMRSL
ATSLSWASLAMGYYLSSVIVSIVNSITGSSGNTPWLRGKSINRYKLDYFYWLMCVLSAAN
FLHYLFWAMRYKYRSTGSRS

AtNRT2.1 (NP_172288.1)
MGDSTGEPGSSMHGVTGREQSFAFSVQSPIVHTDKTAKFDLPVDTEHKATVFKLFSFAKP

HMRTFHLSWISFSTCFVSTFAAAPLVPIIRENLNLTKQDIGNAGVASVSGSIFSRLVMGAVC
DLLGPRYGCAFLVMLSAPTVFSMSFVSDAAGFITVRFMIGFCLATFVSCQYWMSTMFNSQ
IIGLVNGTAAGWGNMGGGITQLLMPIVYEIIRRCGSTAFTAWRIAFFVPGWLHIIMGILVL
NLGQDLPDGNRATLEKAGEVAKDKFGKILWYAVTNYRTWIFVLLYGYSMGVELSTDNVI
AEYFFDRFHLKLHTAGLIAACFGMANFFARPAGGYASDFAAKYFGMRGRLWTLWIIQTA
GGLFCVWLGRANTLVTAVVAMVLFSMGAQAACGATFAIVPFVSRRALGIISGLTGAGGN
FGSGLTQLLFFSTSHFTTEQGLTWMGVMIVACTLPVTLVHFPQWGSMFLPPSTDPVKGTE
AHYYGSEWNEQEKQKNMHQGSLRFAENAKSEGGRRVRSAATPPENTPNNV

AtNRT2.2 (NP_172289.1)
MGSTDEPGSSMHGVTGREQSYAFSVDGSEPTNTKKKYNLPVDAEDKATVFKLFSFAKPH
MRTFHLSWISFSTCFVSTFAAAPLIPIIRENLNLTKHDIGNAGVASVSGSIFSRLVMGAVCD
LLGPRYGCAFLVMLSAPTVFSMSFVSDAAGFITVRFMIGFCLATFVSCQYWMSTMFNSQII
GLVNGTAAGWGNMGGGITQLLMPIVYEIIRRCGSTAFTAWRIAFFVPGWLHIIMGILVLTL
GQDLPGGNRAAMEKAGEVAKDKFGKILWYAVTNYRTWIFVLLYGYSMGVELSTDNVIA
EYFFDRFHLKLHTAGIIAACFGMANFFARPAGGWASDIAAKRFGMRGRLWTLWIIQTSGG
LFCVWLGRANTLVTAVVSMVLFSLGAQAACGATFAIVPFVSRRALGIISGLTGAGGNFGS
GLTQLVFFSTSRFTTEEGLTWMGVMIVACTLPVTLIHFPQWGSMFFPPSNDSVDATEHYY
VGEYSKEEQQIGMHLKSKLFADGAKTEGGSSVHKGNATNNA

Oryza sativa in GenBank:
OsNRT1.1 (ABF94839.1)
MDSSYQHDKPLLDEENSSQVTLEYTGDGSVCIRGHPALRKHTGNWKGSSLAIVFSFCSYL
AFTSIVKNLVSYLTKVLHETNVAAARDVATWSGTSYLAPLVGAFLADSYLGKYCTILIFC
TIFIIGLMMLLLSAAVPLISTGPHSWIIWTDPVSSQNIIFFVGLYMVALGYGAQCPCISSFGA
DQFDDTDENERTKKSSFFNWTYFVANAGSLISGTVIVWVQDHKGWIWGFTISALFVYLGF
GTFIFGSSMYRFQKPGGSPLARICQVVVAAIHKRDKDLPCDSSVLYEFLGQSSAIEGSRKL
EHTTGLKFFDRAAMVTPSDFESDGLLNTWKICTVTQVEELKILIRMFPVWATMILFAAVL
DNMFSTFIEQGMVMEKHIGSFEIPAASFQSIDVIAVLILVPVYERVLVPVFRKFTGRANGIT
PLQRMGIGLFFSMLSMVSAALVESNRLRIAQDEGLVHRKVAVPMSILWQGPQYFLIGVGE
VFSNIGLTEFFYQESPDAMRSLCLAFSLANVSAGSYLSSFIVSLVPVFTAREGSPGWIPDNL
NEGHLDRFFWMMAGLCFLNMLAFVFCAMRYKCKKAS

OsNRT2.1 (BAA33382)
MDSSTVGAPGSSLHGVTGREPAFAFSTEVGGEDAAAASKFDLPVDSEHKAKTIRLLSFAN
PHMRTFHLSWISFFSCFVSTFAAAPLVPIIRDNLNLTKADIGNAGVASVSGSIFSRLAMGAI
CDMLGPRYGCAFLIMLAAPTVFCMSLIDSAAGYIAVRFLIGFSLATFVSCQYWMSTMFNS
KIIGLVNGLAAGWGNMGGGATQLIMPLVYDVIRKCGATPFTAWRLAYFVPGTLHVVMG
VLVLTLGQDLPDGNLRSLQKKGDVNRDSFSRVLWYAVTNYRTWIFVLLYGYSMGVELT
TDNVIAEYFYDRFDLDLRVAGIIAASFGMANIVARPTGGLLSDLGARYFGMRARLWNIWI
LQTAGGAFCLLLGRASTLPTSVVCMVLFSFCAQAACGAIFGVIPFVSRRSLGIISGMTGAG
GNFGAGLTQLLFFTSSRYSTGTGLEYMGIMIMACTLPVVLVHFPQWGSMFLPPNAGAEEE
HYYGSEWSEQEKSKGLHGASLKFAENSRSERGRRNVINAAAAAATPPNNSPEHA

Hordeum vulgare in GenBank:
HvNRT2.1 (AAC49531)
MEVEAGAHGDTAASKFTLPVDSEHKAKSFRLFSFANPHMRTFHLSWISFFTCFISTFAAAP
LVPIIRDNLNLAKADIGNAGVASVSGSIFSRLAMGAICDLLGPRYGCAFLVMLSAPTVFCM
AVIDDASGYIAVRFLIGFSLATFVSCQYWMSTMFNSKIIGTVNGLAAGWGNMGGGATQLI
MPLVFHAIQKCGATPFVAWRIAYFVPGMMHIVMGLLVLTMGQDLPDGNLASLQKKGDM
AKDKFSKVLWGAVTNYRTWIFVLLYGYCMGVELTTDNVIAEYYFDHFHLDLRAAGTIA
ACFGMANIVARPTGGYLSDLGARYFGMRARLWNIWILQTAGGAFCIWLGRASALPASVT
AMVLFSICAQAACGAIFGVAPFVSRRSLGIISGLTGAGGNVGAGLTQLLFFTSSQYSTGRG
LEYMGIMIMACTLPVALVHFPQWGSMFFPASADATEEEYYASEWSEEEKAKGLHIAGQK
FAENSRSERGRRNVILATSATPPNNTPQHV

Triticum aestivum in GenBank:
TaNRT1.1 (AAT69243.1)
MDSTDQFDNSPLLDGDGSSQENTTEYTGDGSVCISGHPASRKHTGNWKASFLIIVCSFCC
YLAYSSIGKNLVSYLTKVLHETNLDAARHVATWQGTSYLAPLVGAFVADSYLGKYRTAL
IACKIFIIGMMMLLLSAALQLISAGPHAWTVWVHLVSSQYTIFLIGLYMVGLGYGAQRPC
VTSFGADQFDDTDYVEKTRKSSFFNWHYFAINAGSLIAGTVIVWVQEHEGWLWGFTISTL
FVTLGVCIFFLGSIVYRFQKPRGSPLTRLCQVVIAATRNFDKVLPCDSSALYEFMGQGSAIE
GRRKLEHTTGLGFFDKAAIVTLPDCESPGQHNKWKICTVTQVEELKILIRMFPIWSAMILF
AAVQEQMSSTFVEQGMAMDKHIGSFEIPSASFQCVDTITVIVLVPIYERLIVPVIRKFTGRA
NGITSPQRIGIGLCFSMFSMVSAALVEGNRLQIAQAEGLVHRKVAVPMSIMWQGPQYFLL
GVAEVFSNIGLTEAFYDESPDGMRSLCMAFSLVNMSAGNYLSSLILSLVPVFTARGGSPG
WIPDNLNEGHLDRFYLMMAGLSFFNIVVFVFCAMRYKCKKAS

TaNRT2.1 (AAG01172.1)
MEVEASAHGDTAASKFTLPVDSEHKAKSFRLFSFANPHMRTFHLSWISFFTCFVSTFAAAP
LVPIIRDNLNLAKADIGNAGVASVSGSIFSRLAMGAICDLLGPRYGCAFLVMLSAPTVFCM
AAIDDASGYIAVRFLIGFSLATFVSCQYWMSTMFNSKIIGTVNGLAAGWGNMGGGATQLI
MPLVFHAIQKCGATPFVAWRIAYFVPGMMHIVMGLLVLTMGQDLPDGNLASLQKKGDM
AKDKFSKVLWGAVTNYRTWIFVLLYGYCMGVELTTDNVIAEYYYDHFHLDLRAAGTIA
ACFGMANIVARPMGGYLSDLGARYFGMRARLWNIWILQTAGGAFCIWLGRASALPASV
TAMVLFSICAQAACGAVFGVAPFVSRRSLGIISGLTGAGGNVGAGLTQLLFFTSSQYSTGR
GLEYMGIMIMACTLPVALVHFPQWGSMFFPASADATEEEYYASEWSEEEKGKGLHITGQ
KFAENSRSERGRRNVILATSATPPNNTPQHV

Lycopersicon esculentum in NCBI:
LeNRT1 (NP_001307053.1)
MGTESQAEINTRKGGLRTMPFIILNESFERIASYGSQTNMIIYLMTYYNMSAATGTSIIGIW
GALSSGLAIVGAIIADCYWGRYNAVAYGTIFTFIGMVILWLTSMIPQLTTLACSHFQHVCN
GPTAFQLAVLFTSFVFTSIGAGFVRPCSIMFGADQLEQKGNPENKKIVESYFNWYYASTG
VATMIAVTVIIYIQDRYGWQIGFGIPVILMVLSVSTFLIGSSLYIKVKPDTNNLLLGLFQAG
AAAFRKRKTPLSLTGCDDYYHSPYETEVLTPSKDFRCLNRACMIEDPERDLNADGSASNP
WNLCSVERVESLKALIRIIPMWSAGFMMFVDMNVFAFSVLQTKTMDRHILPHFEVPAASF

SVFLIIALTIWITFYDRVLVPLLSKYTGRPRGLSPVTRMGIGLTVSCMSMALSAITESIRRKR
AITEGHEDDPNALVNMSAMWFVPQYALLGIAEATHGVGQIEFFYTLLPKSMASIASAMY
TVGTAVSSLIGSILVSSEDWLTSRGGKTSWLSSNINKGHLDYHFWLLALMSFLNLLYFLW
VCRFYETGNDELPHVADEEECDYRLLHES

LeNRT2.3 (NP_001234127.1)
MGEANVKKWACKELKDHRGVLCMESPEENLFSLSPWLLQWCLRIPPPSFQYRWITEHKA
KQFKFYSFSKPHGLTFQLSWISFFTCFVSTFAAAPLVPIIRDNLNLTKMDVGNAGVASVSG
SILSRLTMGAVCDLLGPRYGCAFLIMLSAPTVFCMSFVSSAGGYVAVRFMIGFSLATFVSC
QYWMSTMFNSKIIGLVNGTAAGWGNMGGGATQLIMPLLYDIIRRAGATPFTAWRIAFFIP
GWLHVVMGILVLTLGQDLPDGNRGTLQKTGTVAKDKFGNILWYAATNYRTWIFVLLYG
YSMGVELSTDNVIAEYFFDRFDLKLSTAGIIAATFGMANLLARPFGGFSSDYAAKKFGMR
GRLWVLWILQTLGGVFCVLLGRSNSLPLAVTFMILFSIGAQAACGATFGIIPFISRRSLGIIS
GMTGAGGNFGSGLTQLLFFTSSKYSTATGLTYMGFMIIGSCFSHQQKIQSRERKNIIILQST
QRPERQKGMHQNSLKFAENCRSERGKRVGSAPTPPNLTPNRV

Nicotiana tabacum in GenBank:
NtNRT1.1 (BAC56914.1)
MALPETQQDTKTLPDAWDYKGRPAVRSSSGGWSSAAMILGIEAVERLTTLGIAVNLVTY
LTGTMHLGNASSANNVTNFLGTSFMLTLLGGFVADTFLGRYLTIGIFTTIQAMGVTILTIST
IIPSLRPPKCSPGSSTCIPASSKQLMVLYIALYMTALGTGGLKSSVSGFGSDQFDETDKKER
GQMIKFFNWFFFFINVGSLGAVTVLVYIQDNLGREYGYGICACAIVIGLVIFLSGTRKYRF
KKLVGSPLTQIASVFVAAWNKRHMELPSDSSLLYNIDDIPGDGNKKAKQRLPHSKEFRFL
DKAAIKVQDPESAGITVVNKWNLSTLTDVEEVKLVVRMLPTWATTIMFWTVYAQMTTF
SVSQATTMDRHIGNFEIPPASLTLFFVGSILLTCIFYDRAVVPVCRRVLNNPHGTSPLQRIA
VGLILSIIAMIAAALTEVKRLNVAHLHGLTNDANAKVPLSVFWLVPQFLLVGAGEAFTYI
GQLDFFLRECPKGMKTMSTGLFLSTLSLGFFFSSILVTIVHKVTGKNPWLADNLNQGRLY
DFYWLLATLSVLNLMIFLFISRRYVYKEKRLAECGIEMEDSEPACH

NtNRT1.2 (BAC56915.1)
MALPETQQDSKALPDAWDYKGRPALRSSSGGWASGAMILGVEAVERLTTLGIAVNLVT
YLTGTMHLGNATAANNVTNFLGTSFMLTLFGGFVADTFLGRYLTIGIFATVQAMGVTILT
ISTIIPSLRPPKCEQVGSSSCIPANSKQLMVLYIALYMTALGTGGLKSSVSGFGTDQFDDAD
KKEKGQMIKFFDWFFFFINVGSLGAVTVLVYIQDNLGREWGYGICACAIVIGLVVFLSGT
RKYRFKKLVGSPLTQIASVIVAAWKKRHLELLSDSSLLFEIDDIFGEGNKKNKQKLPHSKE
YRFLDKAAIKEDHDLESNGTNVVINKWKLATLTDVEEVKLLIRMLPTWATTIMFWTVYA
QMTTFSVSQATTMDRHIGTFEIPPASLTVFFVGSILLTVIFYDRVIVPICRRFMNKPHGLTPL
QRIFTGLVLSILAMIAAALTEVKRLKVAHLHGLTNDANATIPLSVFWLVPQFLLVGAGEA
FTYIGQLDFFLRECPKGMKTMSTGLFLSTLALGFFFSSILVTIVHVVTGTTNPWLADNLNQ
GRLYDFYWLLAILSVLNLMFFLYFSRKYVYKEKRLAEMGIELEDDGPVCH

NtNRT2.1 (CAD89798.1)
MGDIEGEPGSSMHGVTGREPVLAFSVASPMVPTDTTAKFSVPVDTEHKAKIFKFYSFSKP
HGLTFQLSWISFFTCFVSTFAAAPLVPIIRDNLNLTKMDVGNAGVASVSGSILSRLVMGAV

CDMLGPRYGCAFLIMLSAPTVFCMSFVSSAGGYVAVRFMIGFSLATFVSCQYWMSTMFN
SQIIGLVNGTAAGWGNMGGGATQLIMPIVYDIIRRAGATPFTAWRIAFFIPGWLHIVMGIL
VLTLGQDLPDGNRGDLQKKGDVSKDKFSNILWYAATNYRTWIFVLLYGYSMGVELSTD
NVIAEYFFDRFDLKLHTAGIIAATFGMANLLARPFGGFSSDYAAKRFGMRGRLWVLWILQ
TLGGVFCVLLGRSNPLPIAVTFMILFSIGAQAACGATFGIIPFISRRSLGIISGMTGAGGNFG
SGLTQLLFFTSSKYSTATGLTYMGLMIIGCTLPVTFCHFPQWGSMFFPPTKDPVKGSEEHY
YAAEYTEAERQKGMHQNSLKFAENCRSERGKRVGSAPTPPNLTPNRV

NtNRT2.2 (CAD89799.1)
MVDIEGSPGSSMHGVTGREPVLAFSVASPMVQTDTTAHFKVPVDSEHKAKVFKFYSFSKP
HGLTFQLSWISFFTCFVSTFAAAPLVPIIRDNLNLTKMDVGNAGVASVSGSILSRLAMGAI
CDMLGPRYGCAFLIMLSAPTVFCMSFVSSAGGYVAVRFMIGFSLATFVSCQYWMSTMFN
SQIIGLVNGTAAGWGNMGGGATQLIMPILYDIIRRAGATPFTAWRIAFFIPGWLHVIMGIL
VLTLGQDLPDGNLASLQKKGDVSKDKFSKILWYAATNYRTWIFVLLYGYSMGVELTTD
NVIAEYFFDRFDLKLHTAGIIAATFGMANLLARPFGGWSSDIAAKHFGMRGRLWNLWIL
QTLGGVFCFLLGKANTLPMAIAWMIIFSLGAQAACGATFGIIPFISRRSLGIISGMTGAGGN
FGSGLTQLLFFTTTKWSTETGLSYMGIMIIACTLPVSLVHFPQWGSMFLPPTKDPVKSTEE
HYFTSEYTEAEKQKGMHQNSIKFAENCRSERGKRVGSALTPPNVTPNRV

Escherichia coli in GenBank:
NARK (CAA34126)
MSHSSAPERATGAVITDWRPEDPAFWQQRGQRIASRNLWISVPCLLLAFCVWMLFSAVA
VNLPKVGFNFTTDQLFMLTALPSVSGALLRVPYSFMVPIFGGRRWTAFSTGILIIPCVWLG
FAVQDTSTPYSVFIIISLLCGFAGANFASSMANISFFFPKQKQGGALGLNGGLGNMGVSVM
QLVAPLVVSLSIFAVFGSQGVKQPDGTELYLANASWIWVPFLAIFTIAAWFGMNDLATSK
ASIKEQLPVLKRGHLWIMSLLYLATFGSFIGFSAGFAMLSKTQFPDVQILQYAFFGPFIGAL
ARSAGGALSDRLGGTRVTLVNFILMAIFSGLLFLTLPTDGQGGSFMAFFAVFLALFLTAGL
GSGSTFQMISVIFRKLTMDRVKAEGGSDERAMREAATDTAAALGFISAIGAIGGFFIPKAF
GSSLALTGSPVGAMKVFLIFYIACVVITWAVYGRHSKK

Hansenula polymorpha in GenBank:
YNT1 (CAA93631)
MQLSTLWEPPIVNPRNLKATSIPIFNLWNVYGRNFFFGWFGFFVCFLSWFAFPPLLHGML
KKDLRLTAVDISNNNICGLTGTLLGRFILGPLNDKYGPRITLTGVLVAGAIPTAFVPLVTN
VAGLHAIRFFISFLGSSFICCSQFCAVFFDNNIIGTANAISAGWGNAGGGVAFFVMPAISNA
LENRGYSLHHSWSYSFVIGPFLILMITAILIFVFGSDCPRGRWSLRGDILGINMDNMLVKSV
SVTRHFSKEGELTSVFVEPVNAIDKVVVEPNQDQEILEVADIINGDEIIEDPSLNDVVKICLS
PRTMLVGLCYMCSFGTELAVESIISNLFGQKMTNWSTSKAGAWGSMLGLLNVVARPAG
GIISDFLYQRFKTTKAKKFWMIFTGLMQGIFLIWIGLVPELSIAGLIVSVSFLCLWFEMGNG
ANYACVPVVNRHHSGIVSGVTGAMGNLGGILFSLVFRYTISNGVNNYFKAFWIIGIVCTA
VNLVCVLIPIREERPRKAEN
