# Supplementary figures and images for: Coding-Sequence Identification and Transcriptional Profiling of Nine AMTs and Four NRTs From Tobacco Revealed Their Differential Regulation by Developmental Stages, Nitrogen Nutrition, and Photoperiod
Source: Front Plant Sci. 2018 Mar 5;9:210. doi: 10.3389/fpls.2018.00210 (PMC5850829; doi:10.3389/fpls.2018.00210)

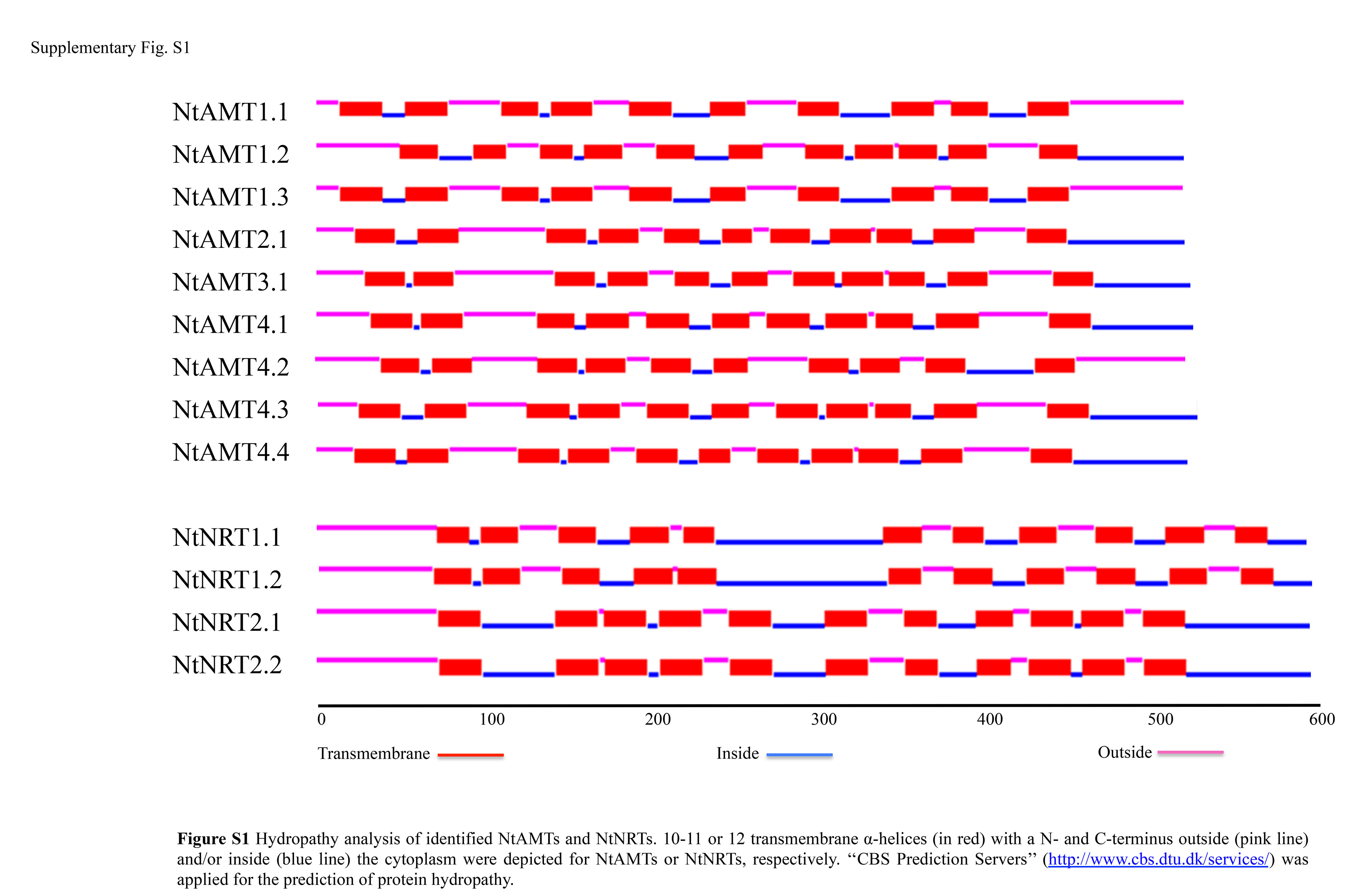

Supplement: Supplementary file 5 [file Image1.JPEG]

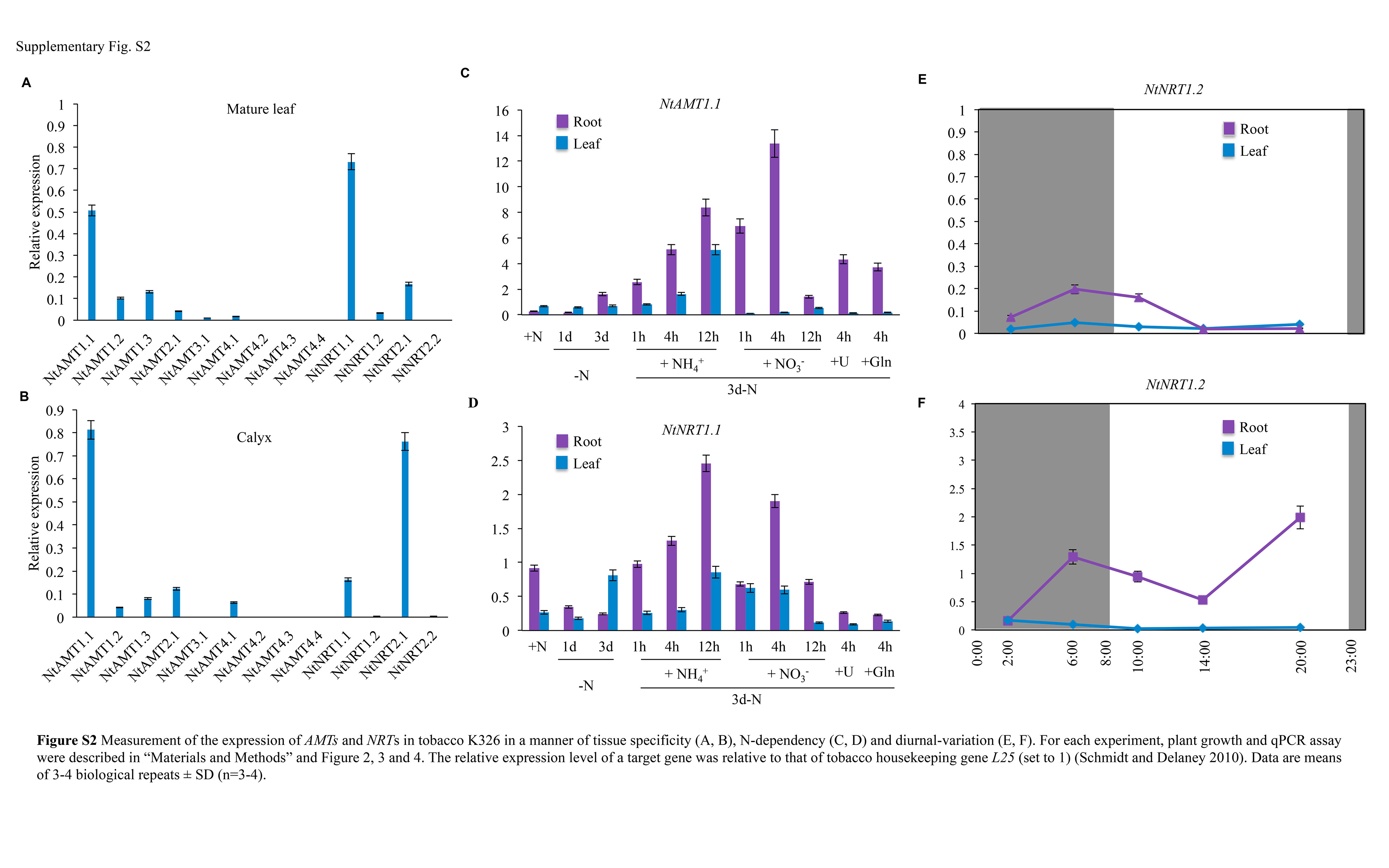

Supplement: Supplementary file 6 [file Image2.JPEG]

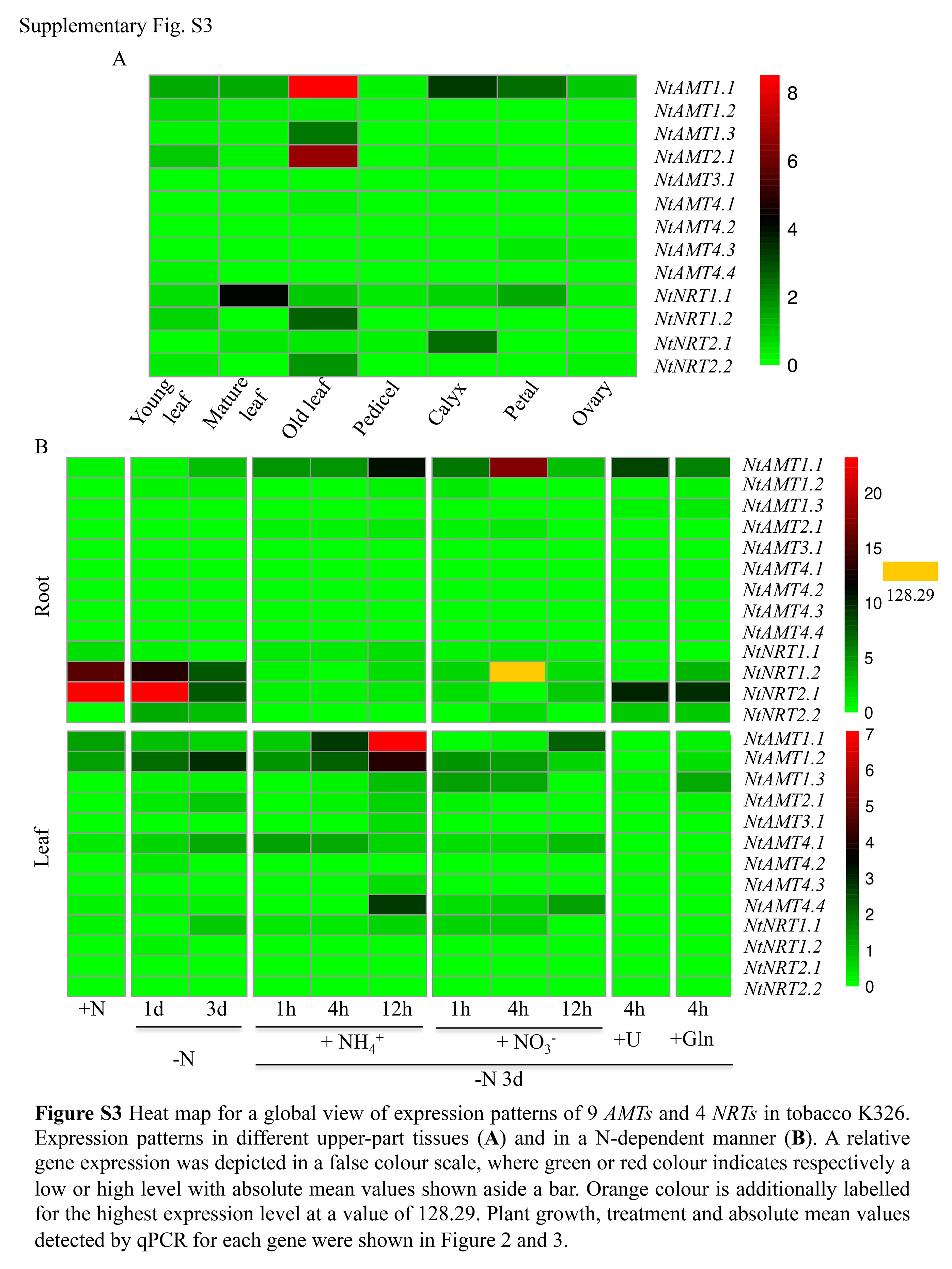

Supplement: Supplementary file 7 [file Image3.JPEG]

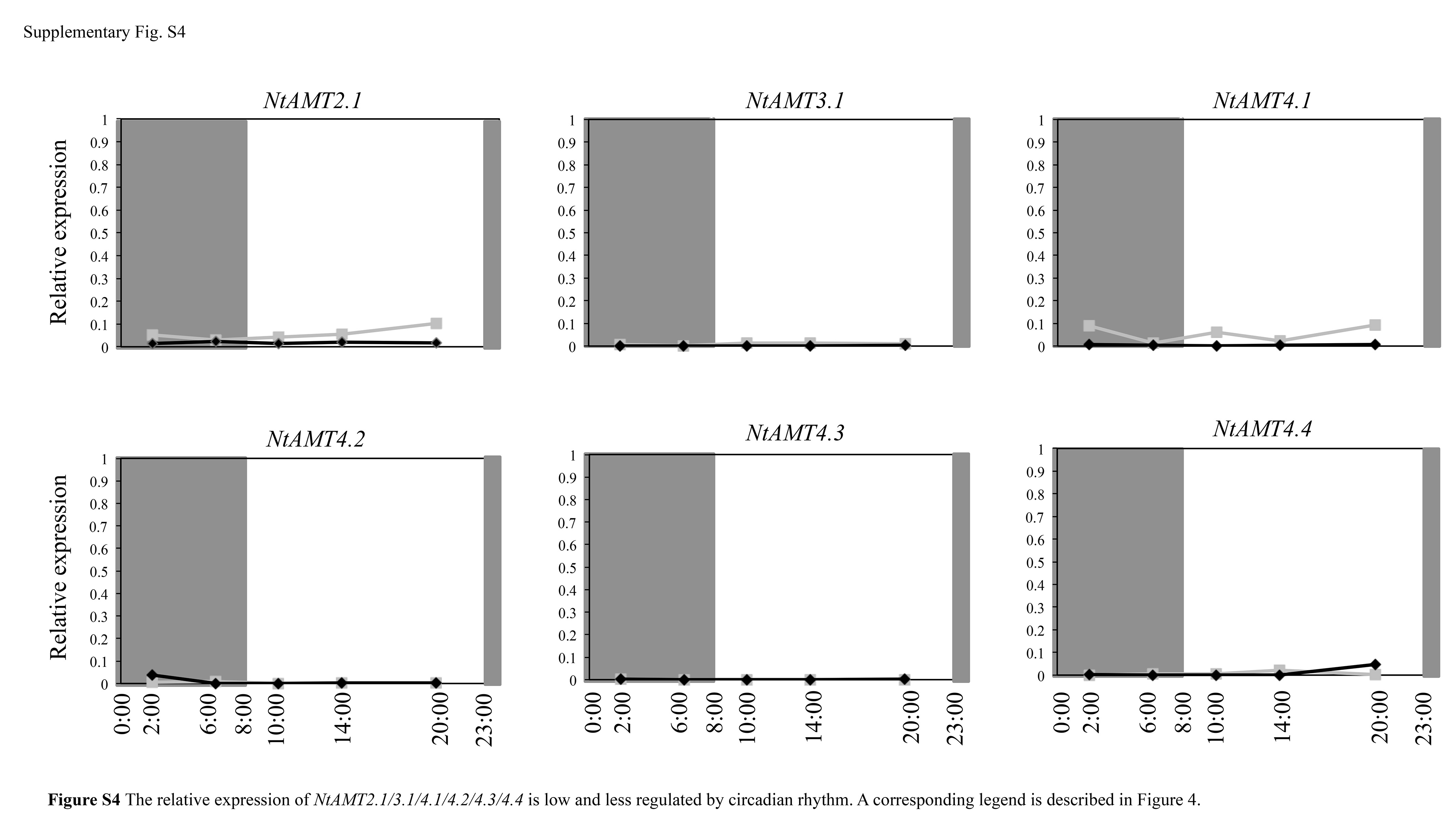

Supplement: Supplementary file 8 [file Image4.JPEG]
